# Engineered ACE2 decoy mitigates lung injury and death induced by SARS-CoV-2 variants

**DOI:** 10.1038/s41589-021-00965-6

**Published:** 2022-01-19

**Authors:** Lianghui Zhang, Soumajit Dutta, Shiqin Xiong, Matthew Chan, Kui K. Chan, Timothy M. Fan, Keith L. Bailey, Matthew Lindeblad, Laura M. Cooper, Lijun Rong, Anthony F. Gugliuzza, Diwakar Shukla, Erik Procko, Jalees Rehman, Asrar B. Malik

**Affiliations:** 1 Department of Pharmacology and Regenerative Medicine and the Center for Lung and Vascular Biology, University of Illinois College of Medicine, Chicago, IL USA; 2 Department of Chemical and Biomolecular Engineering, University of Illinois, Urbana, IL USA; 3 Cyrus Biotechnology, Inc., Seattle, WA USA; 4 Department of Veterinary Clinical Medicine, University of Illinois College of Veterinary Medicine, Urbana, IL USA; 5 Toxicology Research Laboratory, Department of Pharmacology and Regenerative Medicine, University of Illinois College of Medicine, Chicago, IL USA; 6 Department of Microbiology and Immunology, University of Illinois College of Medicine, Chicago, IL USA; 7 Department of Biochemistry, University of Illinois, Urbana, IL USA; 8 Division of Cardiology, Department of Medicine, University of Illinois College of Medicine, Chicago, IL USA

**Keywords:** Biologics, Infectious diseases, Protein design, SARS-CoV-2

## Abstract

Vaccine hesitancy and emergence of severe acute respiratory syndrome coronavirus 2 (SARS-CoV-2) variants of concern (VOCs) escaping vaccine-induced immune responses highlight the urgency for new COVID-19 therapeutics. Engineered angiotensin-converting enzyme 2 (ACE2) proteins with augmented binding affinities for SARS-CoV-2 spike (S) protein may prove to be especially efficacious against multiple variants. Using molecular dynamics simulations and functional assays, we show that three amino acid substitutions in an engineered soluble ACE2 protein markedly augmented the affinity for the S protein of the SARS-CoV-2 WA-1/2020 isolate and multiple VOCs: B.1.1.7 (Alpha), B.1.351 (Beta), P.1 (Gamma) and B.1.617.2 (Delta). In humanized K18-hACE2 mice infected with the SARS-CoV-2 WA-1/2020 or P.1 variant, prophylactic and therapeutic injections of soluble ACE2_2_.v2.4-IgG1 prevented lung vascular injury and edema formation, essential features of CoV-2-induced SARS, and above all improved survival. These studies demonstrate broad efficacy in vivo of an engineered ACE2 decoy against SARS-CoV-2 variants in mice and point to its therapeutic potential.

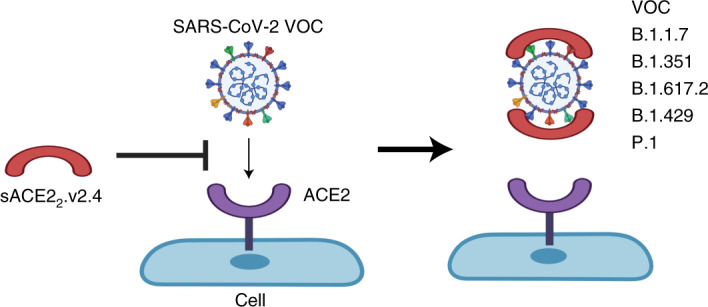

## Abstract (web summary)

Second-generation engineered soluble ACE2 proteins display enhanced binding to the spike protein of SARS-CoV-2 and operate as ‘decoys’ that interfere with viral infection, reduce lung injury and lower mortality in mouse models.

## Main

The coronavirus disease 2019 (COVID-19) pandemic, caused by severe acute respiratory syndrome coronavirus 2 (SARS-CoV-2), resulted in over 8 million deaths worldwide by December 2021 and continues unabated in many regions. Successful development of vaccines inducing immunity against the SARS-CoV-2 spike (S) protein with reported efficacy rates up to 95%^1,2^ has represented a major public health advancement. However, vaccine hesitancy^3^, drastically limited vaccine supply in low-income countries, breakthrough infections and emergence of B.1.1.7 (Alpha), B.1.351 (Beta), P.1 (Gamma) and B.1.617.2 (Delta) SARS-CoV-2 variants of concern (VOCs), carrying S protein mutations with higher affinity for the angiotensin-converting enzyme 2 (ACE2) cell entry receptor that may escape vaccine-induced immunity^4,5^, highlight the urgent need for new targeted therapeutics.

The trimeric S protein on the SARS-CoV-2 surface is a class I fusion protein proteolytically cleaved into soluble S1 and membrane-tethered S2 subunits that are non-covalently associated^6,7^. The S1 subunit contains the receptor-binding domain (RBD), which engages ACE2 as the cell-surface entry receptor^6,7^. The role of the S protein in viral entry has led to the development of monoclonal antibodies (mAbs) binding S epitopes with tight affinity (*K*_D_ < 1 nM)^8,9^,which also harness endogenous host defense mechanisms^10^. However, mAbs also typically show progressively diminished neutralization capacity against some VOCs^11,12^.

An alternative strategy is to use soluble ACE2 (sACE2). To distinguish between monomeric and dimeric ACE2 constructs, we refer to natural, stable sACE2 dimers that include both protease and dimerization domains as sACE2_2_ (refs. ^13,14^). These proteins have the potential to function as decoys for the viral S protein. In a phase 2 trial, wild-type (WT) sACE2_2_ improved oxygenation but did not reduce mortality (ClinicalTrials.gov ID: NCT04335136) and WT sACE2_2_ was ineffective as an Fc fusion protein for virus neutralization in vivo^15^. Thus, we pursued the strategy of identifying and studying the next generation of ACE2 derivatives with the objective that these might prove to be more effective. Deep mutagenesis showed enhanced S affinity after amino acid substitutions within ACE2 (ref. ^14^). A derivative of sACE2_2_ with 3 mutations (T27Y, L79T and N330Y), termed sACE2_2_.v2.4, bound S with 35-fold tighter affinity and neutralized a SARS-CoV-2 isolate on par with high-affinity mAbs^14^. While these data are hopeful, the imperative of defining the efficacy of sACE2_2_.v2.4 in vivo remains.

Standard laboratory mouse strains infected with SARS-CoV-2 do not show disease pathology because murine ACE2 has low affinity for the SARS-CoV-2 S protein^16^. K18-hACE2 transgenic mice express human ACE2 driven by the epithelial K18 promoter and thus develop severe SARS-like inflammatory lung injury and immune dysregulation after SARS-CoV-2 infection^17^. Acute lung injury (ALI) and its progression to fulminant acute respiratory distress syndrome (ARDS) observed in critically ill patients with COVID-19 is characterized by the breakdown of the lung vascular endothelial barrier due to endothelial cell denudation and disruption of the endothelial adherens junction protein VE-cadherin, which together lead to severe lung edema and ultimately respiratory failure^18,19^.

We studied the pharmacokinetics (PK) of sACE2_2_.v2.4-IgG1 in K18-hACE2 mice postadministration via multiple routes and showed tight affinity and persistent binding of the decoy to SARS-CoV-2 and various VOCs. sACE2_2_.v2.4-IgG1 treatment showed exceptional efficacy in mitigating viral entry, lung vascular hyperpermeability and ARDS, and significantly reduced mortality in mice infected with the WA-1/2020 or P.1 VOC.

## Results

### SARS-CoV-2 and VOC induce ARDS in K18-hACE2 transgenic mice

**Fig. 1:**
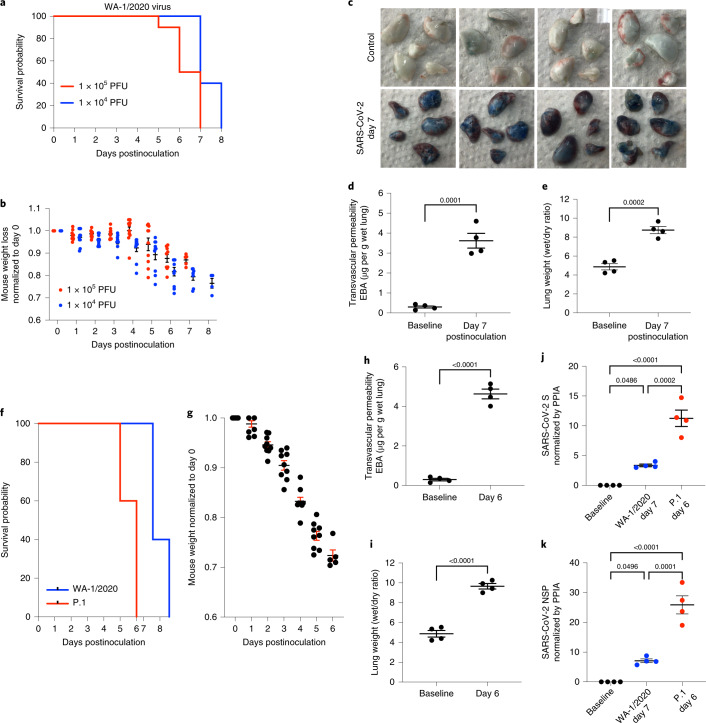
The SARS-CoV-2 isolates WA-1/2020 and P.1 (Brazil) induce severe lung vascular endothelial injury and SARS/ARDS in K18-hACE2 transgenic mice. **a**–**e**, K18-hACE2 transgenic mice were inoculated with SARS-CoV-2 isolate WA-1/2020. **f**–**k**, K18-hACE2 transgenic mice were inoculated with the SARS-CoV-2 isolate P.1. K18-hACE2 transgenic mice not inoculated were used as controls, *n* = 10 mice for each group. **a**,**b**, Mice inoculated with the SARS-CoV-2 isolate WA-1/2020 at 1 × 10^4^ PFU and 1 × 10^5^ PFU, respectively were observed for survival (**a**) and weight (**b**). Only live mice were measured for body weight. **c**–**e**, Cells from mice inoculated with or without SARS-CoV-2 WA-1/2020 at 1 × 10^4^ PFU were collected on day 7. *n* = 4 mice. **c**, Macroscopic images of lungs after EBA tracer injection. **d**,**e**, EBA, as a marker of pulmonary endothelial permeability (**d**), and lung wet/dry ratio, as a measure of lung edema (**e**), were quantified. **f**, Survival curves of mice inoculated with WA-1/2020 or P.1 variant at 1 × 10^4^ PFU. **g**, Weight loss of mice inoculated with P.1. **h**,**i**, Mice were examined on day 6 to evaluate lung vascular leak by EBA assay (**h**) and lung wet/dry ratio (**i**). *n* = 4 mice. RT–PCR was used to measure viral loads in the lungs of mice with the SARS-CoV-2 P.1 variant on day 6 postinoculation and WA-1/2020 strain on day 7 postinoculation. **j**,**k**, mRNA expression of SARS-CoV-2 S (**j**) and SARS-CoV-2 NSP (**k**) is shown. Data are presented as the mean ± s.e.m. PPIA, peptidylprolyl isomerase A. **d**,**e**,**h**,**i**, *P* values were calculated by two-sided Student’s *t*-test. **j**,**k**, *P* values were calculated by one-way ANOVA with Tukey post hoc test. Source data

We inoculated K18-hACE2 mice intranasally with live SARS-CoV-2 WA-1/2020 virus at 1 × 10^4^ plaque-forming units (PFU) (*n* = 10) or 1 × 10^5^ PFU (*n* = 10) and observed progressive body weight loss, a marker of severe viral infection. Higher dosages induced mortality starting at day 5 whereas mice receiving lower dosages died starting on day 7 (Fig. 1a). Weight loss was comparable with both viral doses up to 5 d; however, due to early death of the high-dose group, comparisons were not possible during the later stages (Fig. 1b). We inoculated mice with the lower dose (1 × 10^4^ PFU) of SARS-CoV-2 WA-1/2020 to study lung injury. Lung vascular permeability to protein (a direct quantitative marker of severity of vascular injury) was evaluated at day 7 postinoculation of WA-1/2020 isolate using intravenous infusion of Evans Blue-albumin (EBA) tracer. Representative lung images (Fig. 1c) and quantification of lung EBA, an indicator of lung microvessel permeability of albumin, showed that SARS-CoV-2 induced severe vascular endothelial injury by day 7 (Fig. 1d). Quantification of lung wet/dry ratio (a measure of pulmonary edema and a feature of SARS/ARDS) indicated severe edema (Fig. 1e). Flow cytometry quantification of lung endothelial cells showed that one-third of the cells were lost by day 7 (Supplementary Fig. 1a,b) indicating severe denudation of the endothelium. Quantitative PCR (qPCR) showed that the endothelial adherens junction protein VE-cadherin also decreased by day 7 (Supplementary Fig. 1c), which is indicative of barrier hyperpermeability. Histology demonstrated pulmonary hemorrhage and edema and massive infiltration of immune cells (Supplementary Fig. 1d). Fibrosis staining showed that SARS-CoV-2 induced collagen deposition at day 7 (Supplementary Fig. 1d).

We observed death at days 5 and 6 after intranasal inoculation of K18-hACE2 mice with the P.1 variant (1 × 10^4^ PFU), 2 d earlier than mice infected with the WA-1/2020 isolate (Fig. 1f**)**. P.1 induced significantly greater weight loss (Fig. 1b,g), lung vascular hyperpermeability (Fig. 1h) and edema (Fig. 1i) by day 6. Assessing viral replication by quantifying SARS-CoV-2 S and nonstructural protein (NSP) expression, we found that P.1 viral messenger RNA on day 6 was five times greater than mRNA of WA-1/2020 on day 7 (Fig. 1j,k).

### Molecular basis for tight binding of engineered ACE2 decoy to S protein

The engineered decoy sACE2_2_.v2.4 has three mutations that individually contribute to enhanced ACE2 affinity for S^14^. Modeling and binding free energy calculations predicted that v2.4 mutations enhance S affinity through tighter steric packing and strengthening of existing hydrogen bonds^20^. The basis for affinity enhancement was explored through extensive simulations that considered the dynamics of unbound and bound proteins.

Approximately 400 µs of all-atom molecular dynamics (MD) simulation data were collected for WT and v2.4 ACE2 in the unbound (apo) state. To assess the surface dynamics of interfacial residues, time-lagged independent component analysis (TICA) determined critical residue movements from a higher dimensional dataset of atomic distances (Supplementary Fig. 2a–c). TICA linearly transformed the MD dataset to identify the slowest motions at the interface surface. For both WT and v2.4 ACE2, three time-lagged independent components (TICs) were found with timescales greater than the lag time. The distance features with the highest correlation to the three TICs were the same for both proteins and present near glycosylation sites (Supplementary Fig. 2b–d). Projection of TIC1 and TIC2 showed that these motions displayed qualitatively similar distributions (Supplementary Fig. 2e,f). Furthermore, fluctuations of residues across the simulations were similar (Supplementary Fig. 2g). v2.4 mutations also did not change the folded structure based on residue helicity (Supplementary Fig. 2h). However, ACE2.v2.4 stayed closer in the simulations to the experimentally determined conformation of ACE2 bound to RBD, indicating possible ‘pre-stabilization’ of ACE2.v2.4 in the RBD-bound conformation, which may contribute to the enhanced affinity (Supplementary Fig. 2i,j).

**Fig. 2:**
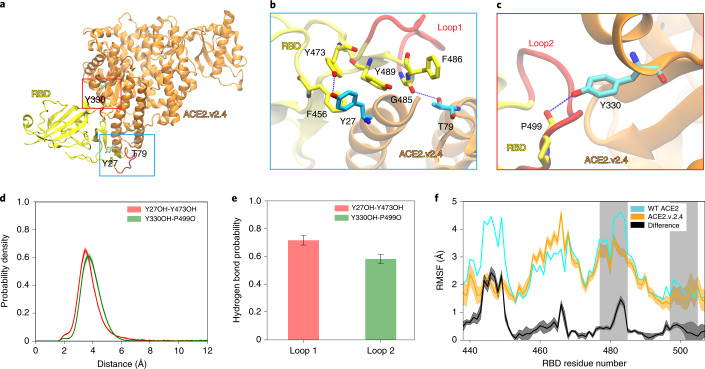
Mutations in ACE2.v2.4 form stabilizing interactions with RBD loops. **a**–**c**, Based on simulations of RBD-bound ACE2, new polar interactions were identified between the ACE2.v2.4 (orange) and RBD (yellow) loops (red) (**a**). ACE2 mutations are presented as sticks colored cyan with nearby RBD residues as sticks colored yellow. New polar interactions between ACE2.v2.4 and RBD loops 1 (**b**) and 2 (**c**) are indicated by the dotted blue lines. **d**, MSM-weighted distance distributions of newly formed hydrogen bonds. For each interacting pair, the ACE2.v2.4 residue is listed first and the RBD residue is listed second. **e**, Probabilities for stable hydrogen bond interactions using a 4-Å distance criterion between accepter and donor. **f**, Root mean square fluctuation (RMSF) of RBD residues when bound to WT (cyan) or v2.4 (orange) ACE2 receptors. The RBD regions that interface with ACE2 are shaded gray. The absolute difference in RMSF between WT and v2.4 proteins is shown in black. Distance distributions, hydrogen bond probabilities and RMSF calculations were based on 40,000 frames from the simulations. Frames were selected based on the MSM stationary probability to represent the entire conformational ensemble. The error bars represent the 95% confidence intervals calculated from 20 bootstrapped samples.

We performed approximately 530 µs of simulation for WT and v2.4 ACE2 proteins bound to RBD. TICA calculations on interface residue distances^21^ showed that the features most correlated with the four slowest components were in RBD loops 1 (residues 477–487) and 2 (residues 497–505) (Supplementary Fig. 3). Trajectory observations showed that new polar interactions were formed by ACE2.v2.4 with the RBD loops: ACE2.v2.4-Y27 to RBD-Y473 and ACE2.v2.4-Y330 hydrogen bonds with the carbonyl backbone of RBD-P499 (Fig. 2a–c). Markov state model (MSM)-weighted distributions demonstrated that the new hydrogen bonds were stable; ensemble distance distributions for interacting atoms were single peaks within approximately 4 Å (Fig. 2d,e). These new interactions by ACE2.v2.4 were associated with reduced fluctuations in RBD loop 1 and to a lesser extent in RBD loop 2 (Fig. 2f), which is consistent with the stabilization of RBD loops at the interface.

### Pharmacokinetics of sACE2_2_.v2.4-IgG1

Before performing in vivo efficacy studies, we determined the PK of the engineered decoy protein. When sACE2_2_.v2.4 (0.5 mg kg^−1^) without a fusion partner was injected intravenously, the protein was cleared with a serum half-life of under 10 min as measured by ACE2 enzyme-linked immunosorbent assay (ELISA) and catalytic activity in serum (Supplementary Fig. 4a,b); this value was less than the half-life of sACE2_2_ of 2–3 h in humans^22^. Toxicity was not seen when sACE2_2_.v2.4 was administered intravenously twice daily at 0.5 mg kg^−1^ for 5 consecutive days (Supplementary Tables 1 and 2).

Soluble receptors are fused to the Fc of IgG1 to increase serum stability and Fc binding improves the efficacy of mAbs targeting SARS-CoV-2 (ref. ^10^). Using an ELISA for human IgG1, both WT sACE2_2_-IgG1 and sACE2_2_.v2.4-IgG1 showed equivalent serum PK after intravenous administration (2.0 mg kg^−1^) in male mice, with protein persisting over 7 d (Supplementary Fig. 4c). Detailed PK assessment in male and female mice demonstrated that the ACE2 moiety was cleared within 24 h with even faster decay in catalytic activity (Supplementary Fig. 5a–c). Immunoblots for human IgG1 confirmed that the fusion protein was proteolyzed to free long-lived IgG1 fragments (Supplementary Fig. 5d). Clearance of sACE2_2_.v2.4-IgG1 was recapitulated in vitro by incubating the protein with normal mouse serum over days but the protein was resistant to degradation in human serum (Supplementary Fig. 5e,f). There was also no evidence of toxicity in mice injected intravenously with sACE2_2_.v2.4-IgG1 at 2.0 mg kg^−1^ (Supplementary Table 1).

We studied the PK of sACE2_2_.v2.4-IgG1 delivered via the intratracheal route, the lungs being the primary and initial site of SARS-CoV-2 infection. After intratracheal delivery, sACE2_2_.v2.4-IgG1 persisted in the lungs for at least 4 h (Supplementary Fig. 5g–i). Any sACE2_2_.v2.4-IgG1 absorbed into the blood was too low for detection. Lung concentrations were high and relatively constant for 2 h after delivery by inhalation (Supplementary Fig. 5j–l). Direct delivery to the lungs resulted in persistence of the peptide for hours but the values did not reach detectable concentrations in plasma, whereas intravenous delivery achieved high but short-lived plasma concentrations. Thus, the delivery route can be individualized and guided by whether the SARS-CoV-2 infection is localized in the respiratory tract or broadly disseminated.

### Prophylactic sACE2_2_.v2.4 prevents SARS-CoV-2 entry and ARDS

**Fig. 3:**
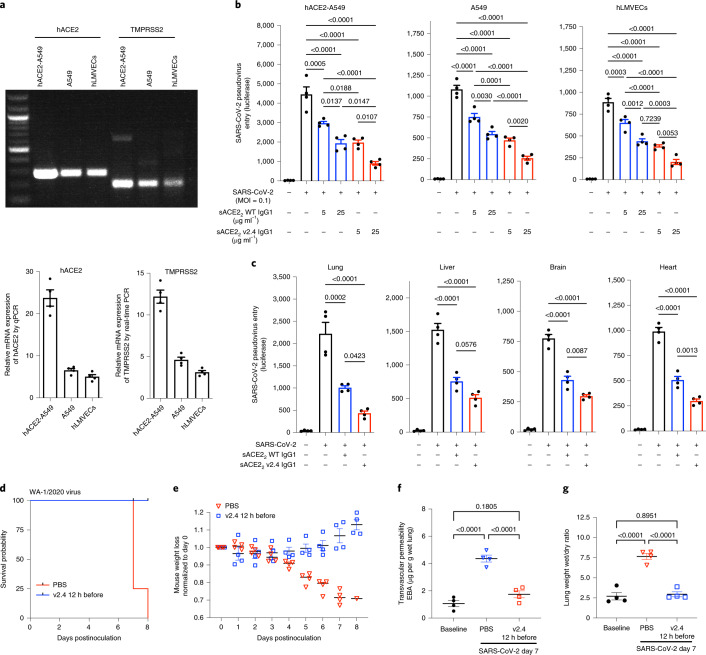
Engineered sACE2_2_.v2.4-IgG1 prevents SARS-CoV-2 pseudovirus cell entry and SARS-CoV-2-induced ARDS. **a**, mRNA expression of ACE2 (NM_001371415.1) and TMPRSS2 (NM_001135099) in human lung epithelial A549 cells. A549 cells stably expressing hACE2 and hLMVECs were analyzed by one-step RT–PCR (top) and real-time PCR (bottom). Relative expression was normalized to glyceraldehyde 3-phosphate dehydrogenase expression. **b**, Cultured hACE2-A549 cells, A549 cells and hLMVECs were preincubated with sACE2_2_-IgG1 or sACE2_2_.v2.4-IgG1 at 5 µg ml^−1^ or 25 µg ml^−1^ for 1 h. SARS-CoV-2 pseudovirus (MOI = 0.1) was added to the cells, which were collected at 24 h. Virus entry was evaluated by luciferase activity. *n* = 4 replicates. **c**, A dose of 10 mg kg^−1^ sACE2_2_-IgG1, sACE2_2_.v2.4-IgG1 or buffer (PBS + 0.2% BSA) was administrated intravenously into K18-hACE2 transgenic mice for 30 min before SARS-CoV-2 pseudovirus (10^6^ PFU) intraperitoneal injection. Tissue lysates were prepared at 24 h and virus entry in the selected organs was evaluated by luciferase activity. Buffer was applied as the control group, *n* = 4. **d**–**g**, K18-hACE2 transgenic mice were inoculated with the SARS-CoV-2 isolate WA-1/2020 at 1 × 10^4^ PFU. Mice received control PBS or sACE2_2_.v2.4-IgG1 10 mg kg^−1^ via intravenous injection 12 h before inoculation. Mice were observed for survival (**d**) and body weight (**e**), *n* = 5. EBA as a marker of pulmonary endothelial permeability (**f**) and lung wet/dry ratio (**g**) as a measure of lung edema were quantified. Data are presented as the mean ± s.e.m., *n* = 4. **b**,**c**,**f**,**g**, *P* values were calculated by one-way ANOVA with Tukey post hoc test. Source data

We next assessed the effects of sACE2_2_.v2.4-IgG1 on viral entry using a replication-deficient pseudovirus expressing the SARS-CoV-2 S protein^11^. Entry is mediated by S engagement of the surface receptor ACE2 and the protease transmembrane protease serine 2 (TMPRSS2) as well as other entry mediators^23^. We investigated the human lung epithelial A549 cell line, primary human lung microvascular endothelial cells (hLMVECs) and A549 cells stably overexpressing human ACE2 (hACE2-A549) to maximize infectability. ACE2 and TMPRSS2 were both expressed in all three cell types but at higher levels in hACE2-A549 cells and at lower levels in hLMVECs (Fig. 3a). After preincubation of cells with sACE2_2_-IgG1 (that is, WT) or sACE2_2_.v2.4-IgG1 at 5 or 25 µg ml^−1^ for 1 h, luciferase-expressing pseudovirus was added (multiplicity of infection (MOI) = 0.1) for 24 h. Pretreatment with sACE2_2_.v2.4-IgG1 showed fivefold greater inhibition of viral entry than WT sACE2_2_-IgG1 in all cell types (Fig. 3b).

Based on the PK results above, 10 mg kg^−1^ sACE2_2_-IgG1 (WT), 10 mg kg^−1^ sACE2_2_.v2.4-IgG1 or control buffer were injected intravenously into K18-hACE2 mice 30 min before pseudovirus (10^6^ PFU intraperitoneally) and mice were assessed at 24 h. Tissue lysates of lungs, heart, brain and liver (Fig. 3c) were used to determine luciferase activity. Viral entry into the lungs was substantially greater than into other organs, which is consistent with preferred viral entry in high hACE2-expressing type II alveolar lung epithelium^21^. Inhibition of pseudovirus entry was significantly greater in sACE2_2_.v2.4-IgG1-pretreated mice (Fig. 3c**)**.

To test the effects of prophylactic delivery of sACE2_2_.v2.4-IgG1 in live SARS-CoV-2 infection, K18-hACE2 transgenic mice were pretreated with a single dose of sACE2_2_.v2.4-IgG1 (10 mg kg^−1^) intravenously 12 h before inoculation with SARS-CoV-2 isolate 1/2020 WA (1 × 10^4^ PFU). PK studies showed that serum concentrations of sACE2_2_.v2.4-IgG1 in mice decreased within hours; a study of ‘long-lived’ mAbs demonstrated measurable efficacy with doses as low as 0.2 mg kg^−1^ administered 24 h before inoculation^24^. Therefore, we determined whether sufficient concentrations of sACE2_2_.v2.4-IgG1 would remain at the delivered dosage to afford protection. All sACE2_2_.v2.4-IgG1-pretreated mice survived with slightly increased or no change in body weight whereas PBS-pretreated mice died by day 8 with 30% loss of body weight (Fig. 3d,e). Furthermore, mice pretreated with sACE2_2_.v2.4-IgG1 showed no significant lung injury or edema and absence of SARS on day 7 postinoculation compared to the marked increases seen in controls (Fig. 3f,g).

### sACE2_2_.v2.4 treatment prevents SARS-CoV-2-induced ARDS

To explore the therapeutic effects of sACE2_2_.v2.4-IgG1 on live SARS-CoV-2 infection, we chose two treatment times: 12 h or 24 h postinoculation based on reports using neutralizing antibodies^24–26^. The times were chosen to begin treatment before the onset of irreversible ARDS and organ damage. Mice inoculated with the higher titer of SARS-CoV-2 (1 × 10^4^ PFU) died within 5–8 d, therefore initiation of treatment at 24 h postinoculation may be equivalent to treating patients at the onset of moderate symptoms.

**Fig. 4:**
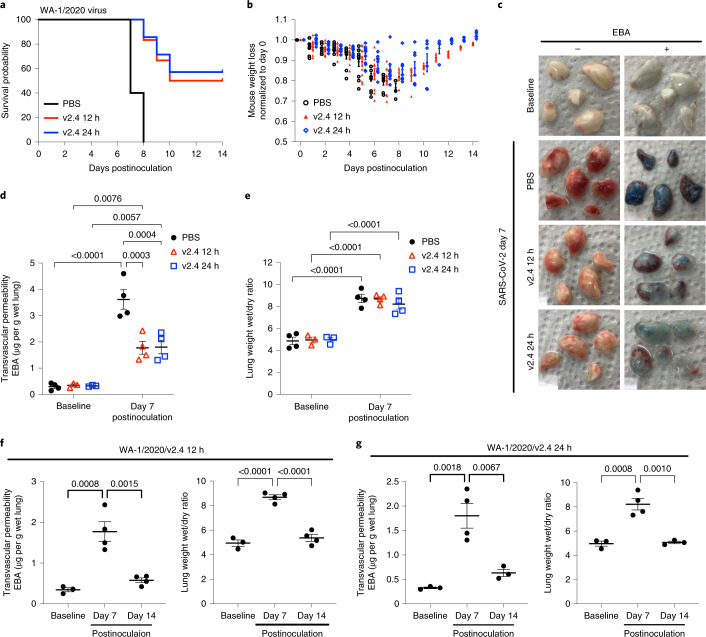
Treatment with sACE2_2_.v2.4-IgG1 mitigates lung vascular endothelial injury and ARDS induced by live SARS-CoV-2 infection and improves survival. K18-hACE2 transgenic mice were inoculated with the SARS-CoV-2 isolate WA-1/2020 at 1 × 10^4^ PFU. Group 1 received control PBS via intravenous injection 24 h postviral inoculation. Group 2 (v2.4 12 h) received sACE2_2_.v2.4-IgG1 10 mg kg^−1^ via intravenous injection 12 h postinoculation and then daily subsequent injections at the same dose. Group 3 (v2.4 24 h) received sACE2_2_.v2.4-IgG1 15 mg kg^−1^ via intravenous injection 24 h postinoculation and then daily subsequent injections at the same dose. **a**,**b**, Survival curves (**a**) and weights (**b**) for *n* = 10 mice for each group. **c**–**e**, Mouse lungs were obtained on day 7 postinoculation for assessment of lung vascular albumin permeability. **c**, Macroscopic images of lungs at baseline and day 7 postviral inoculation in three experimental groups without EBA (left) and with EBA injection (right). **d**, Quantification of EBA lung vascular endothelial permeability in all three experimental groups. **e**, Quantification of lung edema by wet/dry ratio in all three experimental groups at baseline and day 7 postinoculation. **f**,**g**, Time course of lung vascular endothelial permeability and edema formation of groups 2 (v2.4 12 h) (**f**) and 3 (v2.4 24 h) (**g**) as assessed by EBA assay and lung wet/dry ratio. Data are presented as the mean ± s.e.m. **d**,**e**, *P* values were calculated by two-way ANOVA with Tukey post hoc test. **f**,**g**, *P* values were calculated by one-way ANOVA with Tukey post hoc test. Source data

We used a 50% higher protein concentration for the later time (24 h) to increase the likelihood of protection after the prolonged viral replication period in this group. K18-hACE2 mice were inoculated intranasally with SARS-CoV-2 isolate 1/2020 WA (1 × 10^4^ PFU) and then assigned to 3 treatment groups: PBS group (control); sACE2_2_.v2.4-IgG1 (10 mg kg^−1^) treatment group (v2.4 12 h) starting 12 h postinoculation; and sACE2_2_.v2.4-IgG1 (15 mg kg^−1^) treatment group (v2.4 24 h) starting 24 h postinoculation (Supplementary Fig. 6a). Treatment was administered intravenously daily for 7 d. Both the v2.4 12 h and v2.4 24 h treatment groups demonstrated a markedly improved survival of 50–60% at 2 weeks whereas all mice in the control group died with a 30% weight loss (Fig. 4a,b). Treatment with sACE2_2_.v2.4-IgG1 reduced lung vascular albumin hyperpermeability (Fig. 4c,d) and lung edema formation was unchanged at day 7 (Fig. 4e). Viral gene expression was eliminated in lung tissues by day 7 in both sACE2_2_.v2.4-IgG1 treatment groups (Supplementary Fig. 6b,c).

Because of the delay in edema clearance and normalization of vascular permeability, we posited that reduction of edema would be gradual in surviving mice. Therefore, we assessed the time course of lung vascular permeability changes. In surviving mice, lung vascular albumin permeability and edema formation were restored to normal levels by day 14 with either treatment (Fig. 4f,g). Lung histology demonstrated that the v2.4 12 h and 24 h treatments strikingly reduced immune cell infiltration at day 7 compared to the control group (Supplementary Fig. 6d). These results showed profound protection against lung vascular injury, SARS and mortality in mice treated with sACE2_2_.v2.4-IgG1 24 h after the lethal dose of SARS-CoV-2 WA-1/2020.

### High-affinity bindings of sACE2_2_.v2.4-IgG1 to the S proteins of VOCs

**Fig. 5:**
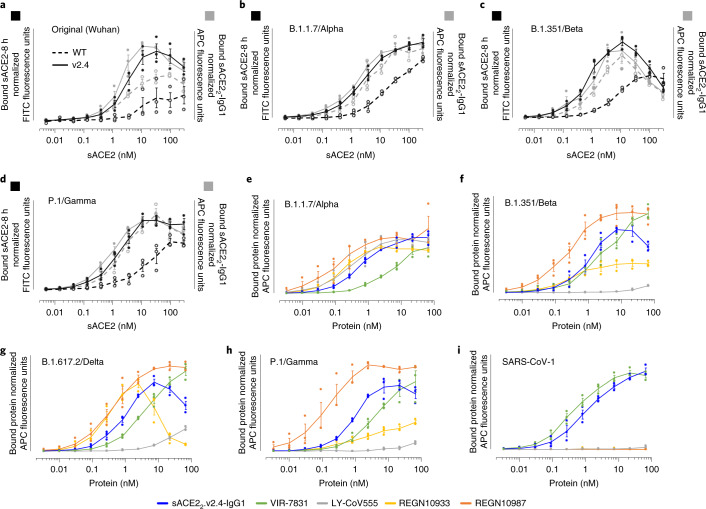
ACE2 decoys carrying the v2.4 mutations bind the S protein from multiple highly transmissible SARS-CoV-2 VOCs and SARS-CoV-1. **a**–**d**, sACE2 carrying the v2.4 mutations has increased S binding compared with WT sACE2. Human Expi293F cells expressing myc-tagged S from 4 SARS-CoV-2 variants (Wuhan (**a**), B.1.1.7/Alpha (**b**), B1.351/Beta (**c**) and P.1/Gamma (**d**)) were incubated with monomeric sACE2-8 h (black) or dimeric sACE2_2_-IgG1 (gray); bound protein was detected by flow cytometry. WT ACE2 proteins are shown as broken lines, v2.4 proteins are shown as solid lines. *n* = 3 independent replicates; data are shown as the mean ± s.e.m. **e**–**i**, Binding of sACE2_2_.v2.4-IgG1 is comparable to clinically effective mAbs. Binding of mAbs versus sACE2_2_.v2.4-IgG1 to the S proteins of SARS-CoV-2 VOCs (B.1.1.7/Alpha (**e**), B1.351/Beta (**f**), B.1.617.2/Delta (**g**) and P.1/Gamma (**h**)) and S protein of SARS-CoV-1 (**i**), as measured by flow cytometry. *n* = 3 independent replicates; data are shown as the mean ± s.e.m. Source data

SARS-CoV-2 variants with increased transmissibility continue to emerge. Virulence due to multiple mutations in S, including changes in immunodominant epitopes that cause partial immune escape^5,11,12^ and substitutions within the RBD that increase ACE2 affinity^27^. Using flow cytometry, we determined the binding of human cells expressing full-length trimeric S to WT sACE2 and sACE2.v2.4 using monomeric sACE2 (ACE2 residues 19–615, a protease domain-only construct) and dimeric sACE2_2_-IgG1 (ACE2 residues 19–732, a region that includes the protease and dimerization domains and forms a stable dimer even before fusion to IgG1 Fc, itself a disulfide-bonded dimer). We assessed monovalent and avid binding, respectively (Fig. 5a–d). Compared to WT, monomeric sACE2.v2.4 showed markedly increased binding to S proteins from original, Alpha, Beta and Gamma variants. Enhanced binding was less when avidity was considered; nonetheless, the engineered derivative remained tightly bound. Indeed, the engineered decoy showed surprisingly increased binding to S from VOCs (Supplementary Fig. 7a), which is consistent with tighter ACE2 affinity. The level of sACE2 bound to S-expressing cells diminished at higher concentrations, suggesting shedding of ACE2-bound S1. Further analysis of sACE2 binding to S from the B.1.429 (Epsilon), B.1.617.2 (Delta) and C.37 (Lambda) lineages again demonstrated tighter binding of sACE2.v2.4 and decreased detection at the cell surface of the N-terminal tag on S, which is consistent with S1 loss (Supplementary Fig. 8).

Monoclonal antibodies have shown mixed results for broad affinity against SARS-CoV-2 variants^24^. Using flow cytometry, binding to S proteins from four VOCs was compared between sACE2_2_.v2.4-IgG1 and mAbs in clinical use (Fig. 5e–h): REGN10933 (casirivimab); REGN10987 (imdevimab); VIR-7831 (sotrovimab); and LY-CoV555 (bamlanivimab). In this assay, sACE2_2_.v2.4-IgG1 consistently showed relatively high binding to the S variants whereas REGN10933 and LY-CoV555 showed widely different binding affinities. REGN10933 and sACE2_2_.v2.4-IgG1 directly engaged the ACE2 interaction motif on S^28^ and both induced S1 shedding at high concentrations. We further noted that only VIR-7831 and sACE2_2_.v2.4-IgG1 bound to the more distantly related S protein of SARS-CoV-1 (Fig. 5i).

Conformational accessibility of epitopes and avidity influence binding to trimeric S at the cell surface. Therefore, we measured monovalent affinities for soluble RBD from the Delta and Gamma VOCs using biolayer interferometry (BLI) (Supplementary Fig. 7b and Supplementary Table 3). VIR-7831 and sACE2_2_.v2.4-IgG1 had the tightest monovalent affinities with excellent breadth. Overall, sACE2_2_.v2.4-IgG1 broadly bound the S proteins of SARS-CoV-2 VOCs with comparable strength to the assessed mAbs, which have shown efficacy in the clinical setting^29^. We also simulated ACE2 carrying the v2.4 mutations bound to RBDs from the Delta and Gamma variants and found that changes in these variants did not eliminate atomic interactions (Supplementary Fig. 9).

### sACE2_2_.v2.4 treatment of SARS-CoV-2 VOCs also mitigates ARDS

**Fig. 6:**
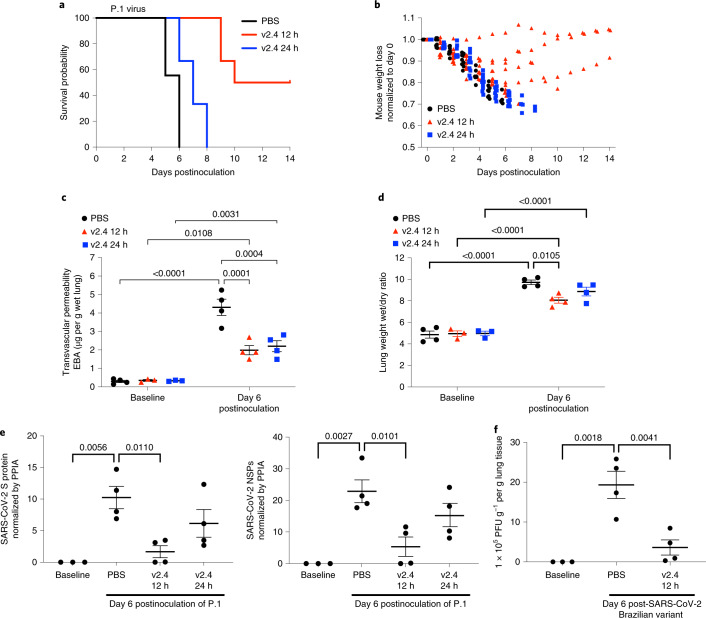
sACE2_2_.v2.4-IgG1 prevents lung vascular endothelial injury and ARDS and improves survival after infection with P.1 VOC. The 3 groups of K18-hACE2 transgenic mice were inoculated with the SARS-CoV-2 variant P.1 (Brazil) at 1 × 10^4^ PFU. Group 1 (PBS): PBS was given by intravenous injection 24 h postinoculation. Group 2 (v2.4 12 h): sACE2_2_.v2.4-IgG1 10 mg kg^−1^ was given by intravenous injection 12 h postinoculation. Group 3 (v2.4 24 h): sACE2_2_.v2.4-IgG1 15 mg kg^−1^ was given by intravenous injection 24 h postinoculation. **a**,**b**, Mice were injected once per day for 7 d. Survival probability was calculated (**a**) and mouse weights were measured (**b**). *n* = 10 mice for each group. **c**,**d**, Mouse lungs were examined on day 6 postinoculation to evaluate lung vascular permeability and lung edema using the EBA assay (**c**) and lung wet/dry ratio (**d**), with baseline mouse lungs as controls. **e**, Viral loads of SARS-CoV-2 in the lungs obtained at baseline and day 6 postinoculation of the SARS-CoV-2 variant P.1 (Brazil) were measured by real-time qPCR for mRNA expression of SARS-CoV-2 S protein and SARS-CoV-2 NSP. **f**, A viral plaque formation assay was performed to measure the viral loads of SARS-CoV-2 in lungs obtained at baseline and on day 6 postinoculation of the SARS-CoV-2 P.1 (Brazil) variant. **c**–**e**,**f**, *n* = 4. Data are presented as the mean ± s.e.m. **c**,**d**, *P* values were calculated by two-way ANOVA with Tukey post hoc test. **e**,**f**, *P* values were calculated by one-way ANOVA with Tukey post hoc test. Source data

Based on our in vitro observations, we addressed whether sACE2_2_.v2.4-IgG1 would also be efficacious in vivo against VOCs. In this study, we tested the P.1 variant inoculated in K18-hACE2 transgenic mice, an aggressive SARS-CoV-2 variant in transmissibility and death in patients^30^. Mice were randomly assigned to three groups: PBS group (control); sACE2_2_.v2.4-IgG1 (10 mg kg^−1^) treatment group (v2.4 12 h) starting 12 h postinoculation; and sACE2_2_.v2.4-IgG1 (15 mg kg^−1^) treatment group (v2.4 24 h) starting 24 h postinoculation. Early treatment with sACE2_2_.v2.4-IgG1 initiated at 12 h postinoculation improved survival to 50–60% whereas delayed treatment initiation (starting at 24 h postinoculation) only delayed death (Fig. 6a). Mice in the v2.4 12 h treatment group showed attenuated body weight loss and survivors gradually recovered weight (Fig. 6b). Both v2.4 12 h and 24 h treatment groups demonstrated markedly reduced lung vascular albumin hyperpermeability (Fig. 6c) and lung edema (Fig. 6d) at day 6, indicating a high level of protection.

Initiating early treatment with sACE2_2_.v2.4-IgG1 caused a greater reduction in viral transcripts in lungs than delayed treatment starting at 24 h (Fig. 6e). Using the viral plaque-forming assay as a measure of viral load in lungs, we found that early sACE2_2_.v2.4-IgG1 treatment reduced the viral plaque-forming capacity on day 6 by 80% (Fig. 6f).

In mice surviving inoculation with the P.1 variant after sACE2_2_.v2.4-IgG1 treatment, lung vascular albumin permeability returned to near-baseline levels by day 14 (Supplementary Fig. 10a,b), indicating full recovery of injured microvessels. Histology demonstrated substantially reduced immune cell infiltration after infection with the P.1 variant at day 7 in the v2.4 12-h and 24-h treatment groups (Supplementary Fig. 10c).

## Discussion

We demonstrated the therapeutic efficacy of sACE2_2_.v2.4-IgG1 in mitigating lung vascular injury, edema formation and mortality after SARS-CoV-2 infection in K18-hACE2 mice, which express human ACE2 in lung alveolar epithelial type II cells^17^. These mice mirror the human setting where most ACE2-expressing cells are alveolar epithelial type II cells^31^. Besides alveolar epithelial cells, other cell types in the lungs, such as macrophages, endothelial cells and dendritic cells also become positive for viral RNA^32^ after the initial infection.

Our study focused on lung endothelial injury because it induces development of lung edema due to vascular leaking of plasma proteins and is a key factor in the progression to respiratory failure and SARS/ARDS. Lung endothelial cells are important in the pathogenesis of lung injury because these cells express higher levels of pro-inflammatory genes compared to the endothelia of other organs^33^. The enrichment of inflammatory genes in lung endothelial cells may therefore increase vulnerability of the lung endothelial barrier cells to the ‘cytokine storm’ elicited by COVID-19 infection^34^. We also observed that SARS-CoV-2 induced marked infiltration of immune cells in mouse lungs, which is consistent with the pathogenic role of macrophages, monocytes, dendritic cells and natural killer cells, which were markedly increased in the lung autopsy samples of patients dying from COVID-19 (ref. ^35^).

The sACE2_2_.v2.4-IgG1 decoy protein prevented infection by blocking binding to ACE2-expressing cells in lungs. The decoy function was superior to native sACE2 due to the higher affinity of the engineered protein for the viral S protein as demonstrated by MD simulations and functional assays. This tight affinity for the S protein explains the high efficacy of the treatment because sACE2_2_.v2.4-IgG1 outcompetes native ACE2 expressed on host cells.

While the emergence of SARS-CoV-2 VOCs has reduced the efficacy of vaccines and neutralizing antibodies^5,36^, our data demonstrate that sACE2_2_.v2.4-IgG1 retains tight affinity for all S variants examined. Furthermore, sACE2_2_.v2.4-IgG1 treatment as late as 12 or 24 h after virus inoculation reduced mortality by 50–60% for both the WA-1/2020 and P.1 viruses. To make valid comparisons among the groups, we studied live mice at the same time point (day 6) for the P.1 variant. Compared to the control group, P.1-infected mice in the 24-h treatment group died 2 d later and half of the mice in the 12-h treatment group survived. By day 6, the 12 and 24 h treatments had prevented the development of SARS-CoV-2-induced severe lung vascular injury and SARS. While treatment at 24 h was too late to fully prevent death from the more virulent P.1 virus, lung vascular leakage, pathology and viral load were nonetheless reduced and survival time was extended. The lack of survival benefits when initiating therapy at later time points may reflect the role of additional pathogenic factors, such as excessive immune cell activation, which may not be adequately reversed when therapy was not initiated at the optimal earlier times.

It is difficult to directly model the time course of human COVID-19 in animal studies; however, our therapeutic regimen matches well those studies using neutralizing mAbs (see Supplementary Tables 4 and 5 for comparisons of prophylaxis and therapy studies). Indeed, the use of a highly lethal infection model in the present studies permitted broader assessment of how engineered protein mitigated lung injury, SARS/ARDS and death. In our model, mice inoculated with a high virus titer (1 × 10^4^ PFU) began to lose weight early by day 2 and all died within 5–7 d. Thus, initiation of treatment at 24 h postviral exposure may correspond to treating human patients at the onset of moderate symptoms.

Although we focused on intravenous delivery for the efficacy studies, PK analysis demonstrated that intratracheally instilled or inhaled protein resulted in stable lung tissue concentrations. Therefore, for future clinical studies, staged therapy where sACE2_2_.v2.4-IgG1 is delivered locally in the lungs via inhalation during the early postexposure period and then systemically via the intravenous route during the later stages after virus dissemination may prove to be most effective.

We also found that sACE2_2_.v2.4-IgG1 markedly reduced SARS/ARDS and mortality in mice infected with the P.1 variant, which is among the most transmissible VOCs with high mortality rates^11,30,37,38^. The protein bound all variant S with similar or even greater affinity than the original SARS-CoV-2 isolate, including S from the B.1.617.2 (Delta) virus, a primary cause of morbidity and mortality worldwide. Based on these data, we surmise that in vivo efficacy will be similar for distinct variants.

Broad affinity of sACE2_2_.v2.4-IgG1 against SARS-CoV-2 variants and other coronaviruses that use ACE2 as an entry receptor^27^ is an advantageous feature of our approach compared to most mAbs where cocktails are used^39^. ACE2-catalyzed turnover of angiotensin and kinin peptide hormones might also directly prevent SARS^40^. These multiple mechanisms of action provide sACE2_2_.v2.4-IgG1 with unique characteristics compared to mAbs in the clinic. While we demonstrated the in vivo efficacy of sACE2_2_.v2.4-IgG1 against two distinct SARS-CoV-2 variants in the K18-hACE2 model, a non-human primate model^41^ would also be useful for studying the effects of varying doses and delivery routes and delivery of the drug at different stages of the disease to identify the optimal combination before initiating human trials.

In summary, we demonstrated that an engineered ACE2 decoy with higher affinity for the SARS-CoV-2 S protein is efficacious in vivo against multiple SARS-CoV-2 variants. The protein prevented viral entry into cells, demonstrated efficacy against a VOC, prevented lung endothelial injury and SARS/ARDS and significantly reduced mortality. The results show the potential of this engineered protein to treat patients with COVID-19 and patients with low antibody titers to protect against emerging, more virulent SARS-CoV-2 variants.

## Methods

### SARS-CoV-2 viruses and cells used for viral replication

The 2019n-CoV/USA_WA1/2020 isolate of SARS-CoV-2 (NR-52281) and the SARS-CoV-2 isolate hCoV-19/Japan/TY7-503/2021 (Brazil P.1) (NR-54982) were obtained from BEI Resources, National Institute of Allergy and Infectious Diseases (NIAID), National Institutes of Health (NIH). Vero E6 (CRL-1586; ATCC) were cultured at 37 °C in DMEM supplemented with 10% FBS, 1 mM sodium pyruvate, 1× nonessential amino acids and 100 U ml^−1^ of penicillin-streptomycin. SARS-CoV-2 was propagated in Vero E6 cells. The supernatant was collected on observation of cytopathic effect. Debris was removed by centrifugation and passage through a 0.22-μm filter. The supernatant was then aliquoted and stored at −80 °C. Virus titers were quantitated by a plaque-forming assay using Vero E6 cells.

### Inoculation of SARS-CoV-2 in K18-hACE2 transgenic mice and administration of sACE2_2_.v2.4

Biosafety level 3 (BSL-3) protocols for animal experiments with live SARS-CoV-2 were performed by personnel equipped with powered air-purifying respirators in strict compliance with NIH guidelines for humane treatment and approved by the University of Illinois Institutional Animal Care & Use Committee (University of Illinois at Chicago protocol no. 18-076). Hemizygous K18-hACE2 mice with a C57BL/6J background (strain no. 034860: B6.Cg-Tg(K18-ACE2)2Prlmn/J) were purchased from The Jackson Laboratory. Animals were housed in groups and fed standard chow. Mice (8–10 weeks old) were anesthetized with ketamine/xylazine (50/5 mg kg^−1^, intraperitoneal). Mice were then inoculated intranasally with 1 × 10^5^ PFU of SARS-CoV-2 isolate WA or 1 × 10^4^ PFU of SARS-CoV-2 isolate WA or P.1 variant suspended in 20 μl of sterile PBS. sACE2_2_.v2.4 was administered intravenously (retro-orbital injection) either 12 h before virus inoculation (single dose) or 12/24 h post virus inoculation (once a day for 7 d) to anesthetized K18-hACE2 transgenic mice. Genotyping of mice was performed by PCR using tail DNA. We conducted an a priori power analysis to calculate the number of animals needed to achieve statistically significant results. We calculated power and sample sizes according to data from pilot experiments; variations within each group of data and variance similarities between groups were compared statistically. Animals with sex- and age-matched littermates were included randomly in the experiments. No animals were excluded due to illness after the experiments. Animal experiments were carried out in a blinded fashion whenever feasible.

### Plaque-forming assay

Vero E6 cells were seeded at a density of 2.5 × 10^5^ cells per well in flat-bottom, 12-well tissue culture plates. The following day, the medium was removed and replaced with 200 μl of tenfold serial dilutions of the material to be titered, diluted in DMEM + 2% FBS. At 1 h, 1 ml of 0.8% agarose overlay was added. Plates were incubated for 72 h, then fixed with 4% paraformaldehyde (PFA, final concentration) in PBS for 30 min. Plates were stained with 0.05% (w/v) Crystal Violet in 20% methanol and washed twice with distilled, deionized water.

### Generation of pseudoviruses

Recombinant vesiculovirus (rVSV) Indiana serotype expressing the SARS-CoV-2 S protein was generated as described previously^42^. HEK 293T cells were grown to 80% confluency before transfection with pCMV3-SARS-CoV-2-spike (Sino Biological) using Lipofectamine 3000 (Invitrogen). Cells were cultured overnight at 37 °C with 5% CO_2_. The next day, the medium was removed and VSV-G pseudotyped ΔG-luciferase (a gift from D. D. Ho, Columbia University) was used to infect the cells in DMEM at an MOI of 3 for 2 h. After infection, cells were washed with PBS twice and new complete DMEM culture medium was added. DMEM supplemented with 2% FBS, 100 IU ml^−1^ penicillin and 100 μg ml^−1^ streptomycin. The supernatant was collected and clarified by centrifugation at 800*g* for 10 min and filtered by 0.45-µm pore size (Merck Millipore) before aliquoting and storing at −80 °C. Before infection of target cells, we incubated viral stock with 20% I1 hybridoma (anti-VSV-G) supernatant (CRL-2700; ATCC) for 1 h at 37 °C to neutralize contaminating G*ΔG rVSV-luciferase particles.

### Virus entry determined by luciferase assay

For the in vitro studies, cultured cells were preincubated with 5 and 25 µg ml^−1^ soluble sACE2_2_-IgG1 (WT) and the sACE2_2_.v2.4-IgG1 variant for 1 h. SARS-CoV-2 pseudovirus (MOI = 0.1) was then added to the cultured cells. Cells were collected for the luciferase assay after 24 h. For the in vivo studies, 10 mg kg^−1^ sACE2_2_.v2.4-IgG1 (WT), sACE2_2_.v2.4-IgG1 and control buffer were administrated intravenously into K18-hACE2 mice for 30 min before SARS-CoV-2 pseudo-entry virus (10^6^ PFU) intraperitoneal injection for 1 d postinoculation. Tissue lysates were prepared and virus entry in the selected organs was evaluated by luciferase activity using the luciferase assay system (Promega Corporation) and a microplate luminometer. Data are shown as the mean ± s.e.m. of *n* = 4 replicates.

### mRNA expression by RT–PCR and real-time qPCR

RNA was extracted from homogenized lung tissues using TRIzol Reagent (catalog no. 15596026; Thermo Fisher Scientific) according to the manufacturer’s protocol. RNA was quantified by NanoDrop 1000 (Thermo Fisher Scientific). One-step PCR with reverse transcription (RT–PCR) amplification was performed using the SuperScript One-Step RT–PCR system with Platinum *Taq* DNA polymerase (catalog no. 10966026; Invitrogen). One microgram total RNA isolated from human cells was used as a template for subsequent one-step RT–PCR. (The primer information is included in Supplementary Table 6.) The one-step RT–PCR products were analyzed by electrophoresis on 1.2% agarose gels containing ethidium bromide. For real-time qPCR, RNA was reversely transcribed into complementary DNA using the High-Capacity cDNA Reverse Transcription Kit (catalog no. 4368814; Thermo Fisher Scientific). FastStart Universal SYBR Green Master (catalog no. 4913850001; Sigma-Aldrich) was used for the relative quantification of cDNA on the ViiA 7 Real-Time PCR System (Thermo Fisher Scientific). (The primer information is included in Supplementary Table 6.)

### Measurement of viral burden

Tissues were weighed and homogenized in 1 ml TRIzol solution. Tissue homogenates were clarified by centrifugation at 10,000 r.p.m. for 5 min and stored at −80 °C. RNA was extracted according to the TRIzol protocol. RNA was reverse-transcribed with Superscript III Reverse Transcriptase (Invitrogen) using random primers. Synthesized cDNA samples were amplified in the ABI PRISM 7000 Sequence Detection System (Applied Biosystems) thermocycler with SYBR Green using primers designed to target a highly conserved region of the S and NSP genes. (The primer information is included in Supplementary Table 6.)

### Reagents

Polyclonal anti-SARS-CoV-2 S glycoprotein was obtained from BEI Resources. The luciferase assay system (E1500) was purchased from the Promega Corporation. Enhanced chemiluminescence (ECL) western blotting detection reagents and nitrocellulose membranes (Hybond ECL) were obtained from Amersham Biosciences Corp. The Lipofectamine 3000 transfection reagents were sourced from Invitrogen. Antibodies for flow cytometry are described elsewhere in the Methods.

### Lung vascular permeability measurement in mice

EBA pulmonary transvascular flux measurements were performed to measure lung vascular leakage. Briefly, 200 μl EBA (1% Evans blue dye (EBD) and 4% albumin in PBS) was administered intravenously (retro-orbital injection) into anesthetized mice and allowed to circulate for 30 min. Mice were euthanized and lungs were perfused by 10 ml PBS. Lung tissues were then excised, weighed, homogenized in 1 ml PBS and extracted overnight in 2 ml formamide at 60 °C. Samples were centrifuged at 10,000*g* for 5 min. The Evans blue concentration in the supernatants of lung homogenates was quantified spectrophotometrically at an absorbance of 620 nm. Tissue EBD content (μg EBD g^−1^ fresh lung tissue) was calculated by comparing tissue supernatant OD_620_ readings with EBD standard curve. The concentration of EBD was determined in micrograms per gram of wet lung tissue. The ratio of wet to dry lung weight for edema measurement was calculated.

### Histology and imaging

Animals were euthanized before the collection and fixation of tissues. Lung lobes were fixed with 4% PFA for 48 h before further processing. Tissues were embedded in paraffin and sections were stained with hematoxylin and eosin, trichrome blue and Sirius Red for collagen deposition. Images were taken with a ZEISS microscope and analyzed with the Zen software version 2 (blue edition, ZEISS).

### Mouse cell isolation

Mouse cells were isolated as described previously^18^. Briefly, flushed mouse lung tissue was minced and digested with 3 ml Collagenase A (catalog no. 10103586001, Roche; 1 mg ml^−1^ in PBS) in a 37 °C water bath for 1 h. Mixtures were titrated with no. 18-G needles and then pipetted through a 40-μm disposable cell strainer. After centrifuging at 300*g* for 5 min and washing with PBS, isolated cells were treated with red blood cell lysis buffer (catalog no. 00-4300-54; eBioscience) for 5 min on ice to lyse red blood cells.

### Flow cytometry

After isolation, cells were incubated with anti-mouse CD16/CD32 (1:50 dilution, catalog no. 553142; BD Biosciences) to block endogenous Fc for 10 min on ice. Cells were stained with antibodies including CD45-EF450 (1:200 dilution, catalog no. 48-0451-82; eBioscience) and CD31-allophycocyanin (APC) (1:100 dilution, catalog no. 17-0311-82; eBioscience) for 45 min at 4 °C. After washing, cells were resuspended in 500 μl buffer and analyzed on an LSRFortessa (BD Biosciences) cell cytometer. Data were analyzed with the Kaluza software version 2.1 (Beckman Coulter). The gating information for the population of mouse lung vascular endothelial cells is shown in Supplementary Fig. 1e–g.

### Cell culture

The 2019n-CoV/USA_WA1/2019 isolate and P.1 variant of SARS-CoV-2 and ACE2-expressing A549 cell lines were obtained from BEI Resources. Vero E6, Vero (catalog no. CCL81; ATCC), ACE2-expressing A549 cells and standard A549 cells were cultured at 37 °C in DMEM supplemented with 10% FBS, 10 mM HEPES (pH 7.3), 1 mM sodium pyruvate, 1× nonessential amino acids and penicillin-streptomycin. Primary hLMVECs from Lonza were cultured in endothelial basal medium-2 supplemented with 10% endotoxin-free FBS (Omega Scientific). Infectious stocks were grown by inoculating Vero cells and collecting the supernatant on observation of cytopathic effect. Debris was removed by centrifugation and passing through a 0.22-μm filter. Supernatant was then aliquoted and stored at −80 °C.

Expi293F cells (Thermo Fisher Scientific) were cultured in Expi293 Expression Medium (Thermo Fisher Scientific) at 37 °C, 125 r.p.m. and 8% CO_2_. Expi293F cells were transfected at a density of 2 × 10^6^ per ml with 400 (pCEP4-myc-ACE2 plasmids) or 500 ng (pCEP4-myc-S and pcDNA3.1-SARS-spike plasmids) per ml of culture using ExpiFectamine 293 (Thermo Fisher Scientific) according to the manufacturer’s instructions. For in vitro binding assays using flow cytometry, transfection enhancers were excluded and cells were collected 24–30 h post-transfection. To produce monomeric sACE2-8 h or RBD-8 h, ExpiFectamine 293 transfection enhancers (Thermo Fisher Scientific) were added approximately 20 h post-transfection and the medium was collected for protein purification 4–6 d later.

Dimeric sACE2_2_-IgG1 proteins were expressed in ExpiCHO-S cells (Thermo Fisher Scientific) cultured in ExpiCHO Expression Medium (Thermo Fisher Scientific) at 37 °C, 125 r.p.m. and 8% CO_2_. Cells were transfected with ExpiFectamine CHO (Thermo Fisher Scientific) according to the manufacturer’s instructions, using 500–1,000 ng plasmid per ml of culture. ExpiFectamine CHO Enhancer (6 μl per ml of culture) and ExpiCHO Feed (240 μl per ml of culture) (Thermo Fisher Scientific) were added 18–22 h post-transfection and the temperature was decreased to 33 °C. At day 5 post-transfection, ExpiCHO Feed (240 μl per ml of culture) was again added. On days 9 and 11, CO_2_ was stepped down to 7% and 6%, respectively. The medium was collected on days 12–14 for protein purification.

### Plasmids

Residue numbers for proteins begin with amino acid 1 as the start methionine. Plasmids for the expression of myc-tagged human ACE2 (pCEP4-myc-ACE2, plasmid no. 141185; Addgene), 8 histidine-tagged monomeric sACE2 (ACE2 amino acids 1–615; pcDNA3-sACE2(WT)-8his, plasmid no. 149268 and pcDNA3-sACE2v2.4-8his, plasmid no. 149664; Addgene), SARS-CoV-1 S protein with C-terminal C9 tag (pcDNA3.1-SARS-spike, plasmid no. 145031; Addgene), 8his-tagged RBD (pcDNA3-SARS-CoV-2-S-RBD-8his, plasmid no. 145145; Addgene) and human IgG1-Fc fused dimeric sACE2_2_ (ACE2 amino acids 1–732; pcDNA3-sACE2-WT(732)-IgG1, plasmid no. 154104 and pcDNA3-sACE2v2.4(732)-IgG1, plasmid no. 154106; Addgene) have been described previously. We used the nG1m1 isoallotype for the IgG1 Fc sequence, which is less immunogenic in humans since it shares an epitope at a polymorphic residue pair (E356/M358) common to other IgG subclasses. Human codon-optimized S (GenBank ID YP_009724390.1) was subcloned into pCEP4 (Invitrogen) from pUC57-2019-nCoV-S(Human); distributed by Molecular Cloud on behalf of H. Yu, Chinese Academy of Medical Sciences) with a N-terminal hemagglutinin leader (MKTIIALSYIFCLVFA), myc-tag (EQKLISEEDL) and linker (GSPGGA) upstream of the mature polypeptide (amino acids V16–T1273). All mutations were made by extension overlap PCR and confirmed by Sanger sequencing (ACGT). Compared to the SARS-CoV-2 S protein reference sequence (GenBank ID YP_009724390.1), the variant S and RBD proteins cloned in this study have the following mutations: P.1 (L18F, T20N, P26S, D138Y, R190S, K417T, E484K, N501Y, D614G, H655Y, T1027I); B.1.351 (L18F, D80A, D215G, ΔL242-L244, K417N, E484K, N501Y, D614G, A701V); B.1.1.7 (ΔH69-V70, ΔY144, N501Y, A570D, D614G, P681H, T716I, S982A, D1118H); B.1.429 (W152C, L452R, D614G); and B.1.617.2 (T19R, G142D, A222V, L452R, T478K, D614G, P681R).

### Purification of sACE2_2_-IgG1

The ExpiCHO-S expression medium was centrifuged (800*g*, 4 °C, 10 min) to remove cells. Tris base (1 M) was added to the supernatant until the pH reached approximately 7.5. The medium was centrifuged again (15,000*g*, 4 °C, 20 min). KanCapA resin (equilibrated in PBS; Kaneka Corporation) was incubated with the supernatant (2 ml resin per 100 ml medium) for 2 h while the sample was rotated at 4 °C. The resin was collected by passing the sample through an empty chromatography column and was washed with 10 column volumes of Dulbecco’s PBS. Protein was eluted with 4 column volumes 60 mM sodium acetate (pH 3.7) into a vessel containing 2 column volumes 1 M Tris (pH 8.0), yielding a final pH of approximately 6. The pH was raised to 7 by the addition of 1 M Tris base (1–2 column volumes). The eluate was concentrated with a 50-kD molecular weight cutoff centrifugal filtration unit (Merck Millipore) and injected on a HiLoad Superdex 200 pg 16/600 gel filtration column (GE Healthcare) with PBS as the running buffer. Peak fractions at the expected molecular weight of a dimer were pooled, concentrated (>30 mg ml^−1^) and aliquots were frozen in liquid nitrogen and stored at −80 °C. Purity was routinely >98% based on SDS–polyacrylamide gel electrophoresis (SDS–PAGE). Concentrations were determined by absorbance at 280 nm using the calculated molar extinction coefficient for the mature, monomeric polypeptide. For the mouse studies, protein aliquots were thawed, diluted to 5 mg ml^−1^ with PBS and filter-sterilized (0.2 μm) ready for use.

### Purification of sACE2-8 h and RBD-8 h

The Expi293F expression medium was centrifuged to remove cells (800*g*, 4 °C, 10 min) and smaller particulates (15,000*g*, 4 °C, 20 min). HisPur Ni-NTA Resin (Thermo Fisher Scientific) equilibrated in PBS was added (0.5 ml resin per 100 ml medium). The sample was rotated at 4 °C for 90 min. The resin was collected by passing through an empty chromatography column and then washed with >20 column volume PBS. Proteins were eluted using a step gradient of PBS containing 20 mM, 50 mM and 250 mM imidazole (pH 8.0, 12 column volumes for each fraction). Imidazole fractions containing pure protein based on Coomassie-stained SDS–PAGE were pooled and concentrated using 10 (RBD-8 h) or 30 kD (sACE2-8 h) molecular weight cutoff centrifugal filtration units (Merck Millipore). Proteins were separated on a Superdex 200 Increase 10/300 GL column (GE Healthcare) with PBS as the running buffer. Peak fractions at the expected molecular weight were pooled, concentrated (approximately 5 mg ml^−1^) and aliquots were snap-frozen and stored at −80 °C. Concentrations were based on ultraviolet (280 nm) absorbance using the molar extinction coefficient for the mature, monomeric polypeptides.

### Toxicology

Animal experimental procedures were reviewed and approved by the University of Illinois Institutional Animal Care and Use Committee (University of Illinois at Chicago protocol no. 20127, 18-076/21-055). The toxicity of each protein was assessed in five female and five male CD-1 IGS mice (8 weeks old). For sACE2_2_.v2.4 without any tags or fusion partner (corresponding to ACE2 residues 19–732; protein provided by Orthogonal Biologics), mice were administered (tail vein, 0.5 mg kg^−1^) protein intravenously twice daily for 5 consecutive days (days 0–4) and euthanized on day 7. Multiple doses were administered due to the anticipated short serum half-life. To assess the toxicity of sACE2_2_.v2.4-IgG1, a single protein dose was administered intravenously (tail vein, 2.0 mg kg^−1^) and mice were euthanized on day 7. Toxicity was assessed by body weight, complete blood count, serum chemistry (Charles River Laboratories) and necropsy/histopathology. No differences were observed compared to vehicle-treated mice.

### PK

Eight-week-old CD-1 IGS mice (three females and three males per time point) were administered protein solutions intravenously. Mice were euthanized and blood was drawn for serum analysis at the designated times. sACE2_2_.v2.4 (without any tags or fusion partner) and sACE2_2_.v2.4-IgG1 were injected via the tail vein at doses of 0.5 mg kg^−1^ and 2.0 mg kg^−1^, respectively. Catalytic activity in serum was measured using the Fluorometric ACE2 Activity Assay Kit (BioVision), sACE2 concentrations were measured by Human ACE-2 ELISA Kit (RayBiotech) and concentrations of the IgG1 Fc moiety were measured by Human IgG ELISA Kit (Immunology Consultants Laboratory). To characterize different routes side by side, proteins were administered to C57BL/6 mice, three males per time point. Animals weighed 25–35 g and were 8–10 weeks old. Administration was via tail vein injection (2.0 mg protein kg^−1^), intubation-mediated intratracheal instillation (1.0 mg protein kg^−1^) or inhalation, where protein solution was nebulized into the animal-holding chamber. Protein solutions were provided sterile-filtered in PBS at 0.4 mg ml^−1^ (intravenous), 1.0 mg ml (intratracheal) or 50 mg ml^−1^ (inhalation). For intratracheal delivery, mice were anesthetized with 2–3% isoflurane/oxygen and protein solution (50 µl of air (150 µl)) was delivered with a blunt 20-G catheter. At each time point, whole blood was collected by terminal bleeds from the orbital sinus into K2EDTA microtainers for plasma preparation. Lung tissue (50–100 mg) was extracted with T-PER Tissue Protein Extraction Reagent (Thermo Fisher Scientific) at 1/20 (weight/volume of reagent) with protease inhibitor. Lung tissue samples were homogenized using the FastPrep-24 5G Instrument, centrifuged (10,000*g*, 5 min) and the supernatants were analyzed. For immunoblot analysis of protein degradation, samples were prepared in reducing (for the ACE2 blots) or nonreducing (for the human IgG1 blots) SDS load dye and separated by PAGE, transferred to polyvinylidene fluoride membrane, blocked with 5% skimmed milk and stained with 1:2,000 horseradish peroxidase (HRP)-conjugated antihuman ACE2 (OTI1D2 clone; Origene) or 1:5,000 HRP-conjugated donkey antihuman IgG (Jackson ImmunoResearch) in Tris-buffered saline containing 0.1% Tween 20 and 1% skimmed milk. Blots were developed with the Clarity ECL Substrate (Bio-Rad Laboratories) and imaged on a ChemiDoc XRS^+^ system (Bio-Rad Laboratories).

### In vitro binding assays to S variants

Expi293F cells were transfected with pCEP4-myc-S plasmids or with pcDNA3.1-SARS-spike as described above. Four volumes of culture were centrifuged (600*g*, 4 °C, 120 s), the pellet was washed with 0.8 volumes of ice-cold PBS-BSA and then cells were resuspended in 1 volume of PBS-BSA. Serial dilutions of purified sACE2 or mAb proteins were prepared in round bottom 96-well trays using PBS-BSA as the diluent. Equal volumes of cells and protein solution were then mixed and incubated at 4 °C on a rotator for 30 min. Plates were centrifuged (600*g*, 4 °C, 90 s) and the supernatant was removed. Cells were washed twice with ice-cold PBS-BSA, then resuspended in PBS-BSA containing 1:100 anti-His fluorescein isothiocyanate (FITC) (chicken polyclonal; Immunology Consultants Laboratory) plus 1:250 anti-myc Alexa 647 (9B11 clone; Cell Signaling Technology) to detect bound sACE2-8 h proteins or 1:250 antihuman IgG-APC (HP6017 clone; BioLegend) plus 1:150 anti-myc-FITC (chicken polyclonal; Immunology Consultants Laboratory) to detect bound sACE2_2_-IgG1 or mAbs. Plates were incubated for another 30 min on a rotator at 4 °C, centrifuged, cell pellets were washed twice and finally cells were resuspended in PBS-BSA for analysis on a BD Accuri C6 flow cytometer. Data were analyzed using CFlow version 1.0.264.15. In all analyses, cells were gated by side scatter-forward scatter to remove debris. To assess protein binding, cells positive for the myc tag on S (excluding cells expressing S from SARS-CoV-1, which was untagged at the extracellular N terminus; in this case there was no further gating) were gated and the mean fluorescence signal for bound sACE2 within the myc-positive gate was measured (Supplementary Fig. 8j–l). To assess surface levels of S, the mean fluorescence signal for the myc tag was measured for the entire cell population. The background fluorescence of negative controls was subtracted to calculate the Δ mean fluorescence. All replicates were from independent, distinct samples and data were normalized based on the total measured fluorescence in each independent experiment.

### MD simulations

For apo and RBD-bound complexes, protein structures were obtained from the Protein Data Bank ID 6M17 (ref. ^43^). Extracellular ACE2 residues 21–730 were considered in the simulations. For the apo simulations, the RBD was removed from the system. Mutations were introduced using PyMOL 2.2.3 (https://pymol.org/2/) and the systems were solvated using transferable intermolecular potential 3P water and 150 mM NaCl using PACKMOL 18.169^44^. Protein residues and glycosylation sites were parameterized using AMBER ff14SB^45^ and GLYCAM06 (ref. ^46^) force fields. The parameters for the Zn^2+^ center of ACE2 were collected using the cationic dummy atom method^47^.

The systems were first minimized and equilibrated before the production runs. Minimization was performed with the steepest descent and conjugate gradient algorithm for 15,000 steps. The minimized systems were heated in NVT ensemble with backbone atoms restrained with a restraining force (*k*(*x*)^2^) and a spring constant of 10 kcal/(mol-Å^2^). The temperature was increased to 300 K in two steps (0–10 K, 10–300 K). Each step was performed for 2 ns. To increase the pressure of the system to 1 bar, the heated systems were simulated in NPT ensemble keeping the restraint on the backbone for another 2 ns. After this step, the restraints were removed to equilibrate the system at 300 K and 1 bar for 44 ns. To maintain the temperature of the systems, Langevin dynamics were used with 2 ps^−1^ collision frequency. A Berendsen barostat was used to maintain the pressure; a 2-fs time step was used for the simulation. To avoid instability of the bonds involving hydrogen atoms, the SHAKE algorithm^48^ was used. To consider nonbonded interactions, a 10-Å cutoff distance was selected. Long-range electrostatic interactions were considered using the particle mesh Ewald method. Minimization and equilibration were done using the AMBER18 engine.

After equilibration, production runs were performed with adaptive sampling to utilize parallel computing power. Adaptive sampling^49^ is a parallel iterative process to sample the protein conformational space by running many short-length simulations. In adaptive sampling, the protein conformational space is characterized by important features, such as distances, angles and root mean square deviation (RMSD), and the feature space is clustered into microstates. The starting points for the next round of simulation are selected from the least populated states. The first five rounds of adaptive sampling were performed using the AMBER engine at the Blue Waters supercomputing facility. The features selected for the adaptive sampling were backbone RMSD. Data collected over 5 rounds of adaptive sampling were clustered into 500 clusters, from which 500 structures were selected to submit to Folding@Home^50^. Each structure was given 20 different starting velocities to begin 20 different trajectories. Simulations were run in the OpenMM^51^ engine. For each apo system, approximately 400 µs of data were collected; for each RBD-bound (original/Wuhan variant) complex, approximately 530 µs of data were gathered. For the Delta and Gamma RBD-bound systems, approximately 10 µs of data were collected for each system.

An MSM was built on MD data to obtain unbiased estimates of the canonical ensemble of interface movements. The MSM calculates the canonical ensemble distribution from the eigenvector space of the transition probability matrix. To estimate the transition probability matrix, we featurized the MD simulation data with interresidue distances between the human and viral protein interfaces. The featured space was linearly transformed into TICs. The TIC space was clustered into microstates using the *k*-means algorithm. The probability of a jump between one microstate (*i*) to another (*j*) with a particular lag time ($\tau$) corresponds to the *T*_*ij*_ element of the transition probability matrix, *T*. Feature calculations were performed with MDTraj 1.9.3. The TIC calculation, clustering and MSM building steps were done using PyEMMA 2.5.6^52^. The lag time for MSM was selected by logarithmic convergence of implied timescales. To build the optimal MSM, VAMP2 scores of the MSMs were calculated using different hyperparameters (TIC variance and numbers of clusters). Feature calculations and analysis of the trajectories were performed using MDTraj^53^ and CPPTRAJ 18.01^54^. For trajectory visualization, the VMD 1.9.3 software was used^55^.

Error analysis in the MD data was performed with bootstrapping. In each bootstrapped sample, 80% of the total trajectories were used to make MSMs with the same clustering levels. Twenty bootstrapped samples were calculated for each system with the same MSM hyperparameters.

### Anti-SARS-CoV-2 mAbs

The sequences for mAbs that had received emergency use authorization from the U.S. Food and Drug Administration were pulled from the Kyoto Encyclopedia of Genes and Genomes (accession nos. REGN10933, D11938; REGN10987, D11939; VIR-7831, D12014; LY-CoV555, D11936). H and L chains were cloned with CD5 leader sequences into pcDNA3.1(+) and expressed in Expi293F cells. Proteins were purified using the KanCapA resin (Kaneka Corporation) and size exclusion chromatography with PBS as the running buffer. Peak fractions at the expected molecular weight of approximately 150 kD were concentrated, frozen in liquid nitrogen and stored at −80 °C. Concentrations were determined using absorbance at 280 nm and the calculated extinction coefficient for mature protein.

### BLI

BLI kinetics were collected on an Octet RED96a and analyzed with a 1:1 binding model (global fit) using instrument software (Molecular Devices). mAbs and sACE2_2_.v2.4 were immobilized at 100 nM for 60 s to antihuman IgG Fc capture biosensors (Molecular Devices). The assay buffer was 10 mM HEPES (pH 7.6), 150 mM NaCl, 3 mM EDTA, 0.05% polysorbate 20 and 0.5% nonfat dry milk (Bio-Rad Laboratories). Loaded sensors were equilibrated for 30 s in buffer, then dipped in RBD-8 h solution for 60 s to measure association and transferred back to the buffer to measure dissociation over 300 s.

### Statistics and reproducibility

Quantification of replicate experiments is presented as the mean ± s.e.m. A Student’s *t*-test, one-way analysis of variance (ANOVA) and two-way ANOVA with Tukey post hoc test were used to determine statistical significance, with a *P* value threshold <0.05. Significance levels are indicated in the figures as *P < 0.05, **P < 0.01 and ***P < 0.001. Based on our experience, we expected changes in the gene/protein expression and function measurements to be detected with 4 mice per group, so the effect size was determined as *n* = 4 independent mice. The variance between groups that were being statistically compared was similar.

### Study approval

All aspects of this study were approved by the office of Environmental Health and Safety at the University of Illinois at Chicago before the start of this study. Working with SARS-CoV-2 was performed in a BSL-3 laboratory by personnel equipped with powered air-purifying respirators.

### Reporting Summary

Further information on research design is available in the Nature Research Reporting Summary linked to this article.

## Online content

Any methods, additional references, Nature Research reporting summaries, source data, extended data, supplementary information, acknowledgements, peer review information; details of author contributions and competing interests; and statements of data and code availability are available at 10.1038/s41589-021-00965-6.

## Supplementary information

Supplementary Information Supplementary Figs. 1–10 and Tables 1–6.

Reporting Summary

Supplementary Data Uncropped images and numerical data for Supplementary Fig. 1.

Supplementary Data Numerical data for Supplementary Fig. 4.

Supplementary Data Numerical data for Supplementary Fig. 5.

Supplementary Data Uncropped images and numerical data for Supplementary Fig. 6.

Supplementary Data Numerical data for Supplementary Fig. 7.

Supplementary Data Numerical data for Supplementary Fig. 8.

Supplementary Data Uncropped images and numerical data for Supplementary Fig. 10.

## Data Availability

MD data with features for MSM building and an embedded Box link are available from GitHub (https://github.com/ShuklaGroup/ACE-RBD_simulation_data_script.git). Other data are included with the paper. Source data are provided with this paper.

## Source data

Source Data Fig. 1 Uncropped images and numerical data for Fig. 1.
Source Data Fig. 3 Uncropped images and numerical data for Fig. 3.
Source Data Fig. 4 Uncropped images and numerical data for Fig. 4.
Source Data Fig. 5 Numerical data for Fig. 5.
Source Data Fig. 6 Numerical data for Fig. 6.
