# Supplementary material for: Engineered ACE2 decoy mitigates lung injury and death induced by SARS-CoV-2 variants
Source: Nat Chem Biol. 2022 Jan 19;18(3):342–51. doi: 10.1038/s41589-021-00965-6 (PMC8885411; doi:10.1038/s41589-021-00965-6)
Supplement: Supplementary file 1 — Supplementary Figs. 1–10 and Tables 1–6. [file 41589_2021_965_MOESM1_ESM.pdf]

nature portfolio

https://doi.org/10.1038/s41589-021-00965-6

Supplementary information

Engineered ACE2 decoy mitigates lung injury and death induced by SARS-CoV-2 variants

In the format provided by the authors and unedited

nature portfolio

https://doi.org/10.1038/s41589-021-00965-6

Supplementary information

Engineered ACE2 decoy mitigates murine lung injury and death induced by SARS-CoV-2 variants

In the format provided by the authors and unedited

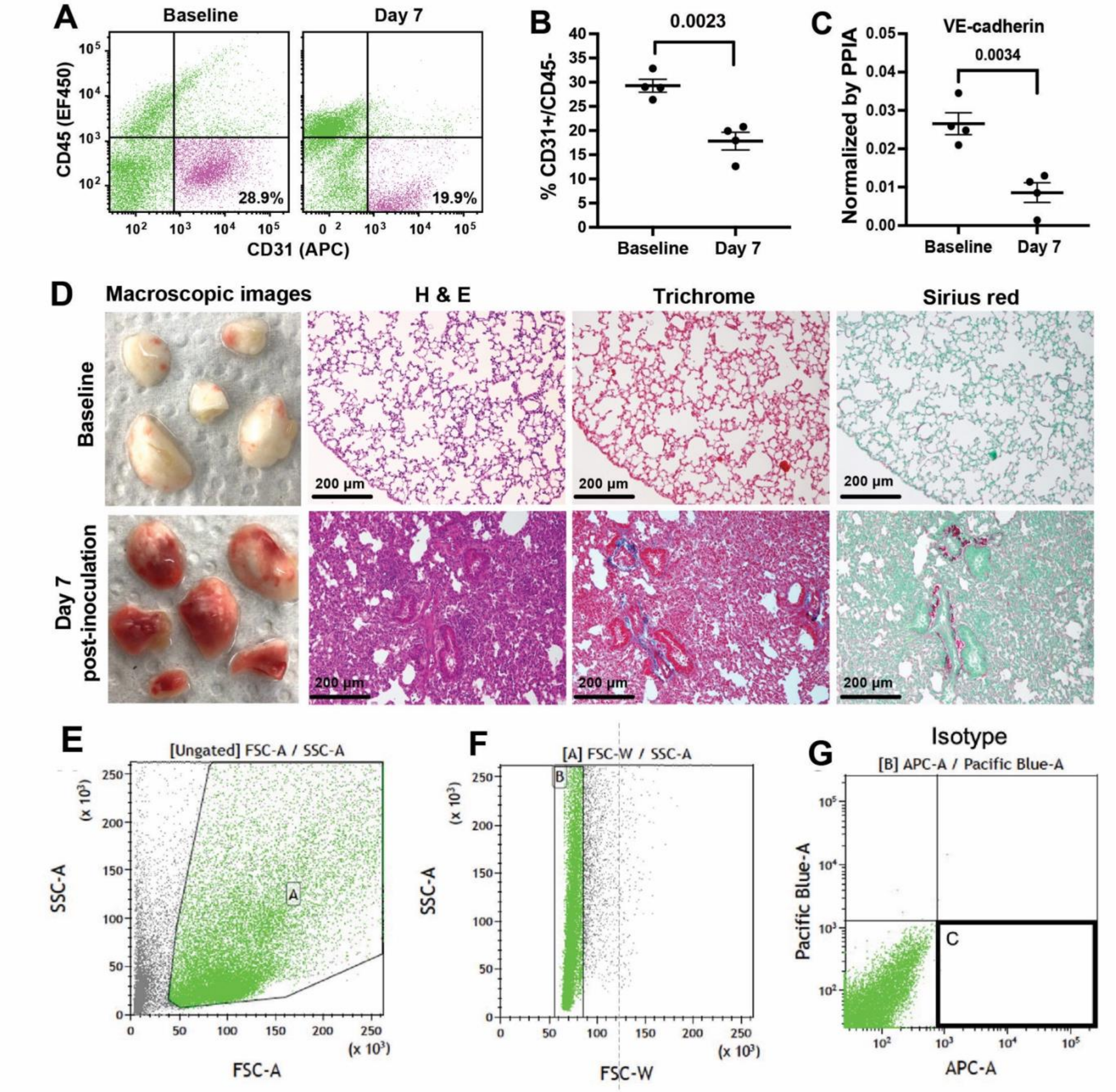

Supplementary Figure 1. SARS-CoV-2 isolate WA-1/2020 induces severe lung vascular endothelial injury and ARDS in K18-hACE2 transgenic mice (Extended figures from Figure 1).
K18-hACE2 transgenic mice were inoculated with SARS-CoV-2 isolate WA-1/2020. (**A-B**) Representative flow cytometry plots (**A**) and quantitative analysis (**B**) of CD31+/CD45- lung ECs as a percentage of total lung cells at baseline and day 7 post-inoculation. (**C**) Quantification of Cdh5 mRNA expression normalized by the housekeeping gene PPIA (peptidylpropyl isomerase A). N=4. Data are mean ± SEM and P values are shown in **B** & **C** by Student's t- test. (**D**) Representative macroscopic lung images (1st column), histology images of lung sections with H & E staining (2nd column), and Masson Trichrome (3rd column) and Sirius Red (4th column) staining for collagen deposition at baseline (1st row) and day 7 post-inoculation (2nd row). The lungs from 4 independent mice were sectioned, stained, and imaged. (**E-G**) Gating for measuring the population of mouse lung endothelial cells by flow cytometry. Whole lung cells were gated to exclude debris (**E**) and clusters (**F**). Cells were stained with APC anti-CD31 and Pacific Blue anti-CD45 and gated as shown (**G**); lower-right quadrant C represents lung vascular endothelial cells (CD31+/CD45-).

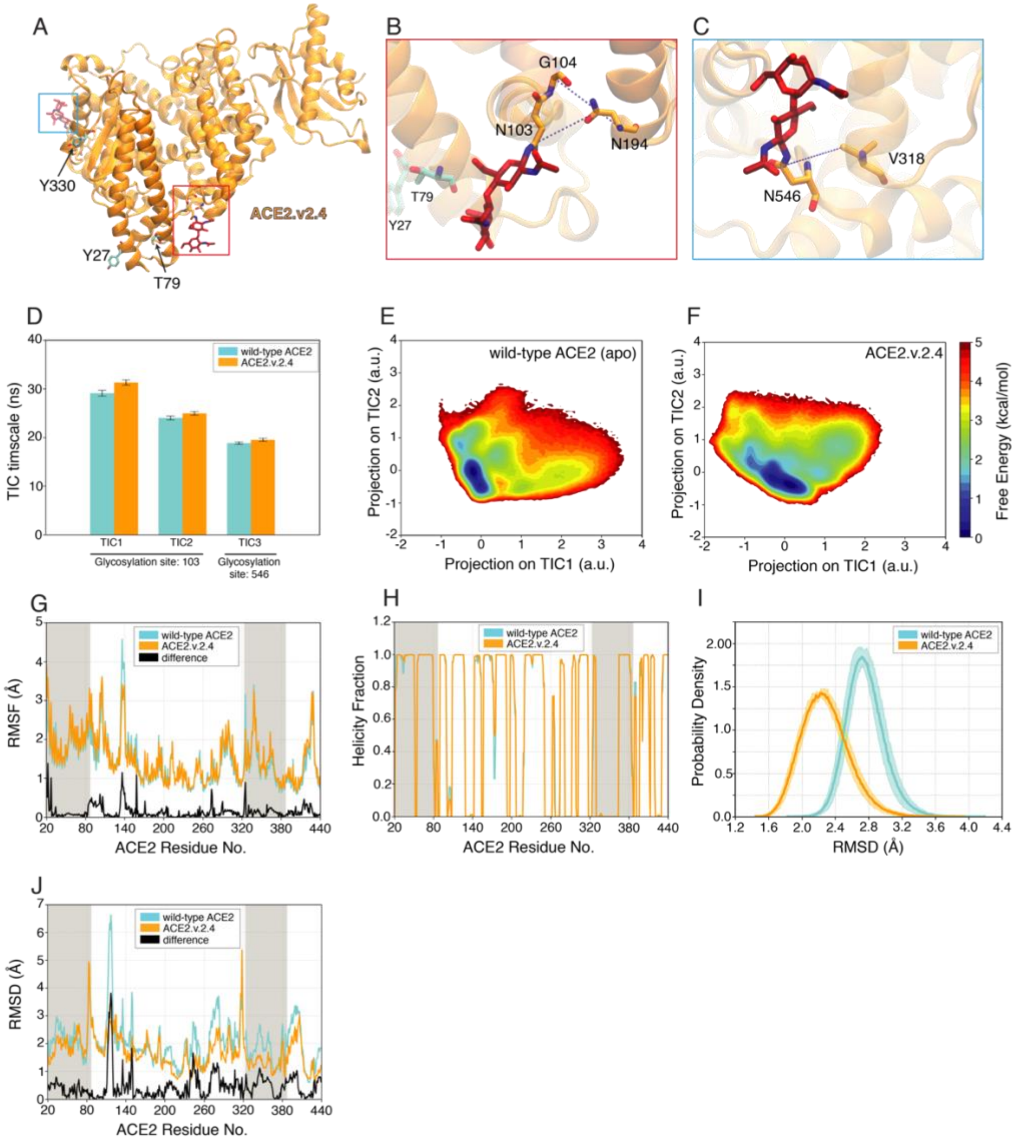

Supplementary Figure 2. Simulated dynamics of unbound (apo) wild type ACE2 and ACE2.v2.4 are similar. (**A**) ACE2.v2.4 (orange ribbon) with sites of mutations and glycans shown as cyan and red sticks, respectively. (**B and C**) For ACE2 residues at the interface with RBD, backbone and sidechain distances were calculated with respect to all other residues within 0.8 nm. A total of 74 distances were used for TICA calculations. Highest correlated features to the slowest TICs are represented as sticks. Correlated distance features are shown with dotted blue lines. Distance features with highest correlation to TIC 1 and TIC 2 components are near the N103 glycan (red box, **B**), while those with highest correlation to the TIC 3 component are near the N546 glycan (blue box, **C**). (**D**) TIC timescales for the three slowest TIC components. WT, cyan; v2.4, orange. (**E** & **F**) Free energy landscapes for the projections of TIC 1 and TIC 2 for (**E**) WT and (**F**) v2.4 ACE2. (**G**) Root mean square fluctuation (RMSF), (**H**) helicity fraction, and (**I**) root mean square deviation (RMSD, calculated with respect to the RBD-bound ACE2 structure; PDB 6M17) of WT (cyan) and v2.4 (orange) proteins based on apo simulations. ACE2 regions near the RBD interface are shaded grey. (**J**) RMSD to PDB 6M17 is plotted across the ACE2 sequence, with absolute difference between the two simulated systems in black. In the apo simulations, ACE2.v2.4 stays closer to the RBD-bound conformation at multiple sites. RMSF, helicity fraction, and RMSD are calculated based on 40,000 simulation frames selected based on the MSM stationary probability to represent the entire conformational ensemble. Errors indicate 95% confidence intervals calculated from 20 bootstrapped samples.

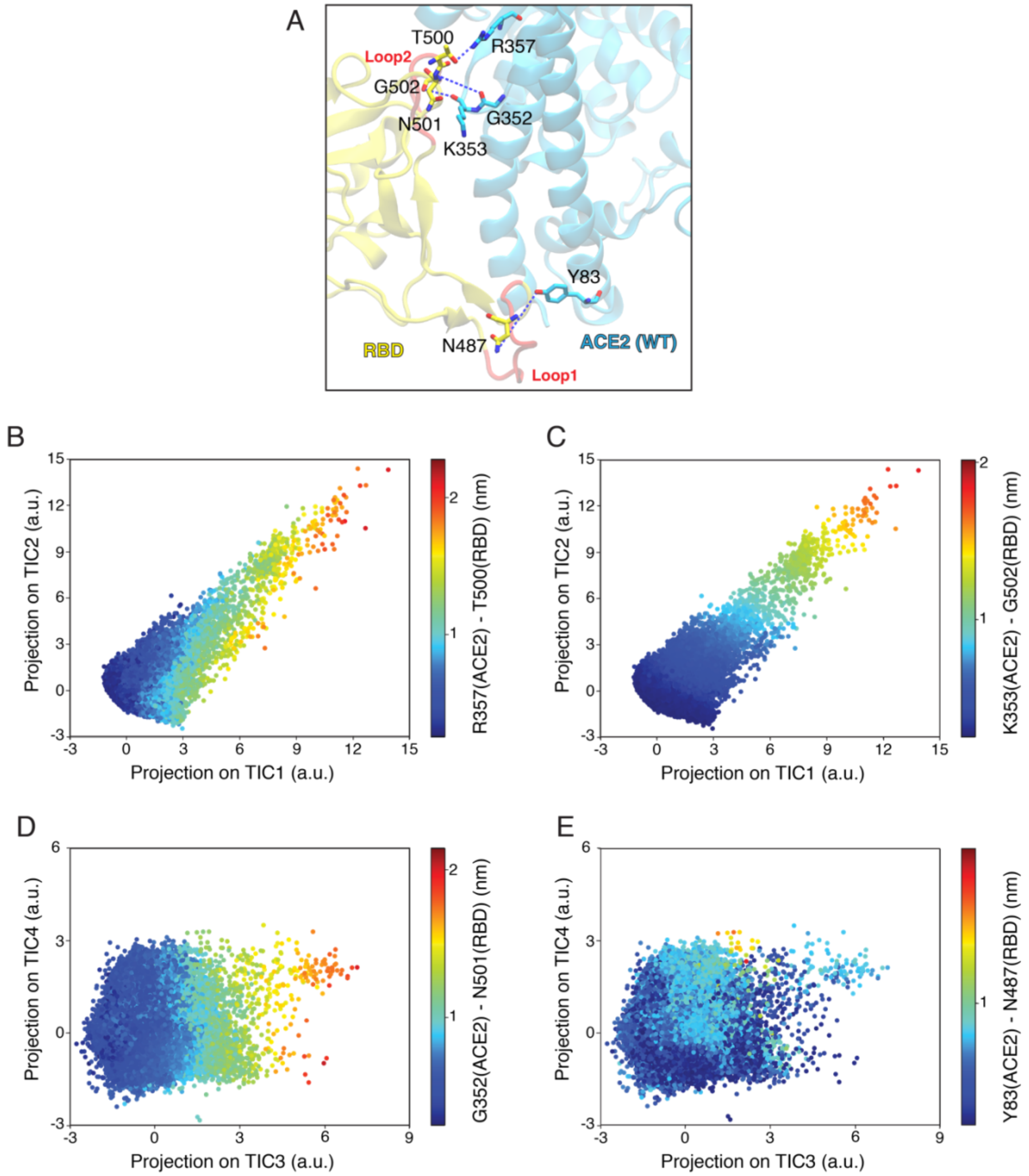

Supplementary Figure 3. TIC correlations with interface residue distances. (**A**) For the complex of wild type ACE2 (blue) with RBD (yellow), distance features correlated with the slowest TICs are represented. RBD loops 1 and 2 are red. (**B, C**) Projection of TIC 1 coordinate with respect to TIC 2. Color scales are defined based on interaction distances with highest correlation to TIC 1 (**B**) or TIC 2 (**C**). (**D, E**) Projection of TIC 3 coordinate with respect to TIC 4. Color scales are defined based on interaction distances with highest correlation to TIC 3 (**D**) or TIC 4 (**E**).

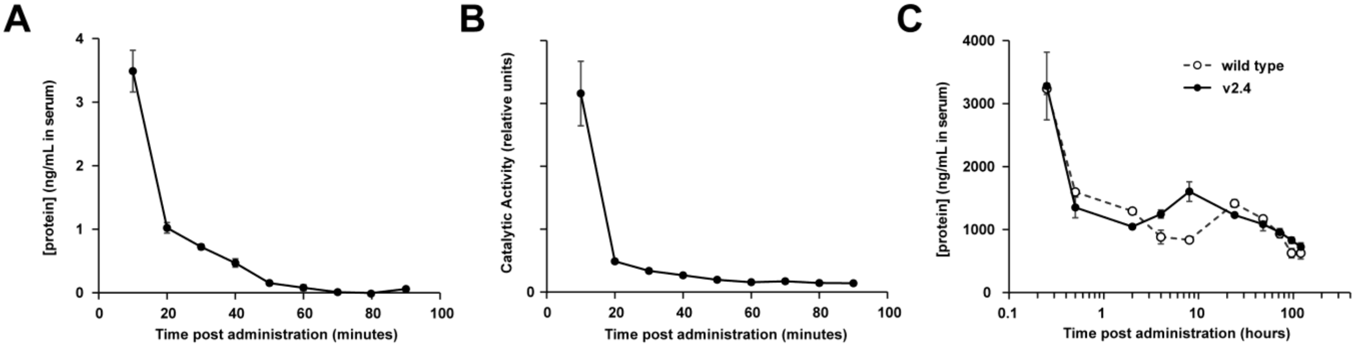

Supplementary Figure 4. Serum PK of IV administered sACE2$_2$.v2.4 and IgG1-fusion proteins. (**A** & **B**) Unfused sACE2$_2$.v2.4 was injected in the tail veins of mice (3 male and 3 female per time point; 0.5 mg/kg). Serum was analyzed (**A**) by ACE2 ELISA and (**B**) for proteolytic activity towards a fluorogenic substrate. (**C**) IV administration of 2.0 mg/kg wild type sACE2$_2$-IgG1 (white circles) or sACE2$_2$.v2.4-IgG1 (black circles) in 3 male mice per time point. Protein in serum was quantified by human IgG1 ELISA. Data are mean ± SEM.

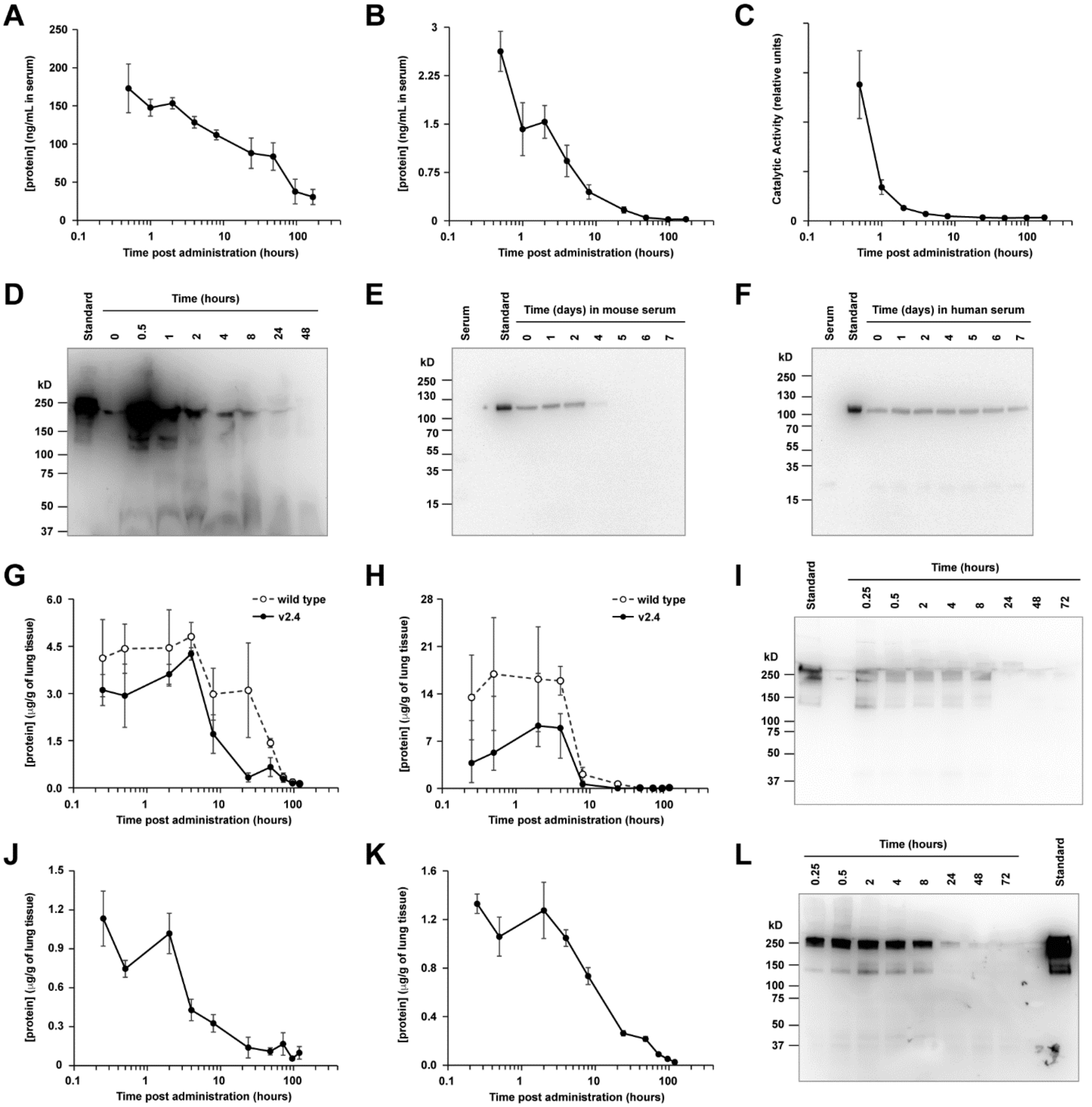

Supplementary Figure 5. Pharmacokinetics of sACE2$_2$.v2.4-IgG1 protein. (**A**-**C**) sACE2$_2$.v2.4-IgG1 was IV administered to mice (N=6 per time point; 2.0 mg/kg). Serum was collected and analyzed by human IgG1 ELISA (**A**), by ACE2 ELISA (**B**), and for ACE2 catalytic activity (**C**). (**D**) Serum samples from representative male mice were separated on a non-reducing SDS electrophoretic gel and probed with anti-human IgG1 using 10 ng of purified sACE2$_2$.v2.4-IgG1 as a standard. Predicted molecular weight (MW; excluding glycans) of dimer is 216 kD. (**E**-**F**) sACE2$_2$.v2.4-IgG1 was incubated in vitro at 37 °C with normal mouse (**E**) and human (**F**) serum. Samples were separated on a reducing SDS gel and immunoblotted with anti-human ACE2. MW of monomer (excluding glycans) is 108 kD. Shown are representative blots from two experiments. (**G**-**H**) Wild type sACE2$_2$-IgG1 (white circles) and sACE2$_2$.v2.4-IgG1 (black circles) were administered IT at 1.0 mg/kg. Proteins were extracted from lung tissues and analyzed by (**G**) human IgG1 ELISA and (**H**) ACE2 ELISA. N=3 males per time point. (**I**) Lung extracts from representative mice IT administered sACE2$_2$.v2.4-IgG1 were analyzed under non-reducing conditions by anti-human IgG1 immunoblot. (**J**-**K**) Mice inhaled nebulized sACE2$_2$.v2.4-IgG1 for 30 minutes. Lung tissue extracts were analyzed by (**J**) ACE2 ELISA and (**K**) human IgG1 ELISA. N=3 males per time point. (**L**) Lung tissue extracts of mice receiving nebulized sACE2$_2$.v2.4-IgG1 were analyzed by anti-human IgG1 immunoblot. Data are presented as mean ± SEM.

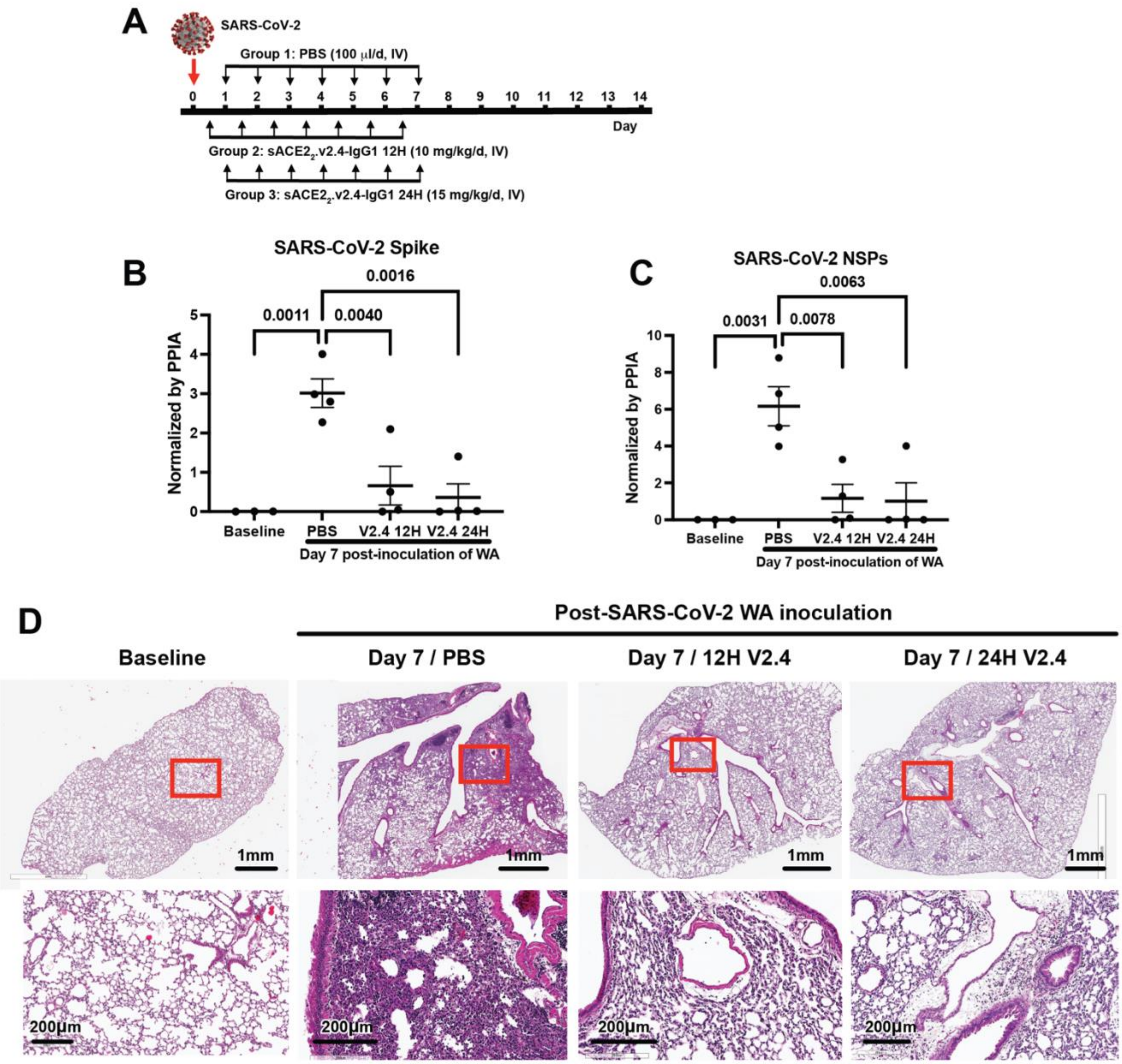

Supplementary Figure 6. Treatment with sACE2$_2$.v2.4 IgG1 mitigates lung vascular endothelial injury and ARDS induced by live SARS-CoV-2 infection (Extended figures from Figure 4).
(**A**) Experimental design to test therapeutic efficacy. K18 hACE2 mice were inoculated by SARS-CoV-2 isolate WA-1/2020 at 1×10^4 PFU. Group 1 received control PBS via IV injection 24 h post viral inoculation. Group 2 (V2.4 12H) received sACE2$_2$.v2.4-IgG1 10mg/kg via IV injection 12 h post inoculation, and then daily subsequent injections at the same dose. Group 3 (V2.4 24H) received sACE2$_2$.v2.4-IgG1 15mg/kg via IV injection 24 h post inoculation and then daily at the same dose. (**B-C**) Viral load in the lungs harvested at Day 7 measured by real-time quantitative PCR for the mRNA expression of SARS-CoV-2 Spike and SARS-CoV-2 NSPs. N=4, Data are mean ± SEM and P values are shown in **B** & **C** by one-way ANOVA. (**D**) Representative H&E staining of lung sections at baseline (1st column), control PBS group at day 7 post-inoculation (2nd column), sACE2.V2.4-IgG 12H treatment group at Day 7 (3rd column), and sACE2.V2.4-IgG 24H treatment group at Day 7 (4th column) post-inoculation. The images in the first row are low magnifications. Rectangle areas (Red) are shown in higher magnification in the second row. The lungs from 4 independent mice were sectioned, stained, and imaged. The lungs from 4 independent mice were sectioned, stained, and imaged.

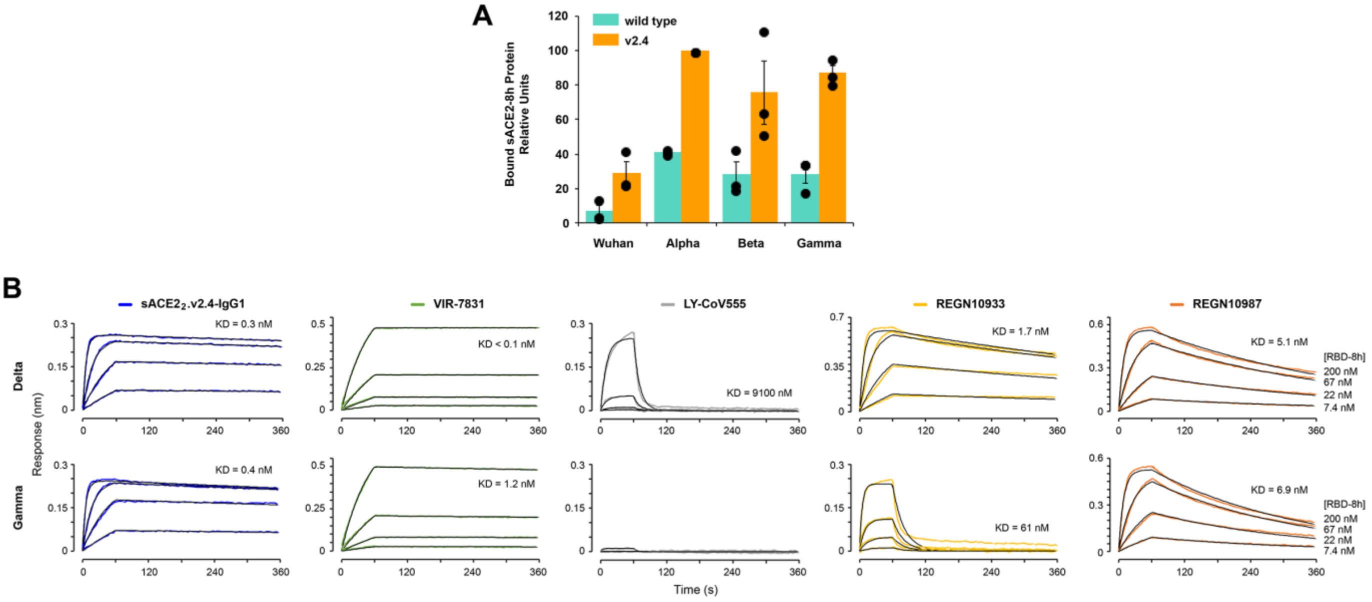

Supplementary Figure 7. Tight binding of soluble ACE2 decoys to S from highly transmissible SARS-CoV-2 variants.

(**A**) Expi293F cells expressing full length S proteins from the Wuhan, Alpha, Beta, and Gamma SARS-CoV-2 variants were incubated with 11 nM monomeric sACE2-8h (cyan) and sACE2.v2.4-8h (orange) and bound protein was detected by flow cytometry. Fluorescence signals for bound proteins are shown relative to sACE2.v2.4-8h binding Alpha, which had the highest signals. N=3 independent replicates, mean ± SEM. (**B**) BLI binding kinetics of delta and gamma RBDs for $sACE2_2$.v2.4-IgG1 versus monoclonal antibodies. Sensors displaying immobilized $sACE2_2$.v2.4-IgG1 or mAbs were transferred to solutions of monomeric RBD (time = 0 to 60 s) to measure association and then returned to buffer to measure dissociation (time = 60 to 360 s). RBD concentrations are indicated on the rightmost sensorgrams. Upper panels show binding to delta RBD and lower panels show binding to gamma RBD. Measured responses are colored while fitted curves (1:1 binding model) are black. See Table S3 for full statistics.

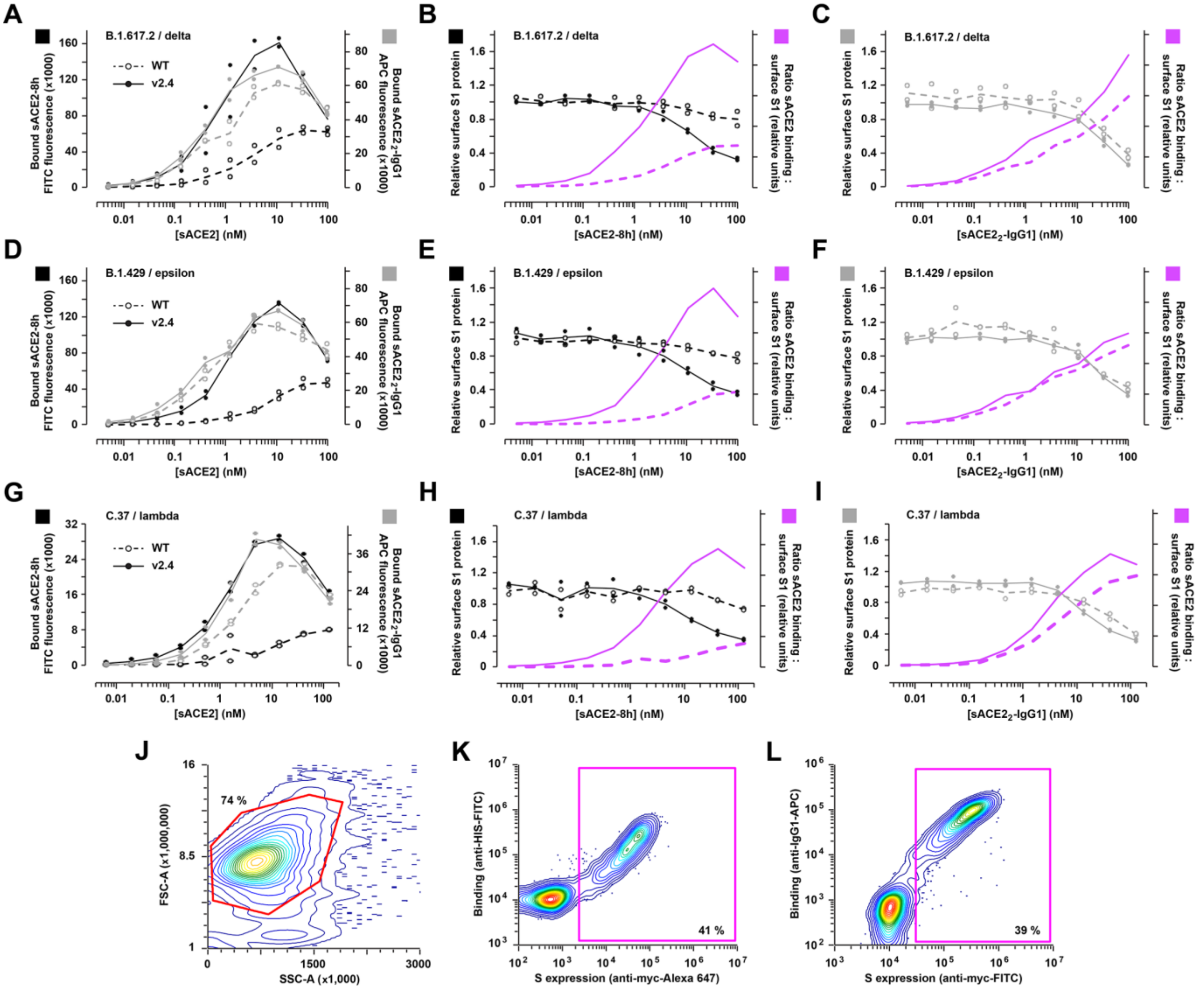

Supplementary Figure 8. Decoy receptors diminish S1 at a membrane surface.

Human Expi293F cells expressing myc-S from the B.1.617.2 (**A-C**), B.1.427/B.1.429 (**D-F**), or C.37 (**G-I**) lineages were incubated with monomeric sACE2-8h (black) or dimeric sACE2$_2$-IgG1 (grey), washed, and then stained for flow cytometry (N = 2). (**A, D, G**) Bound wild type (dashed lines, open circles) and v2.4 (solid lines, filled circles) sACE2 proteins. (**B-C, E-F, H-I**) Relative surface S1 protein based on detection of the myc tag, after incubating cells with sACE2-8h (**B, E, H**) or sACE2$_2$-IgG1 (**C, F, I**). The ratio of bound decoy receptor to surface S1 is shown in magenta. (**J-L**) Gating strategies for measuring ACE2-S binding by flow cytometry. (**J**) Human Expi293F cells transfected with myc-tagged S were washed, incubated with soluble ACE2 and mAbs, and stained with fluorescent antibodies to detect surface S expression and bound soluble proteins. Cells were analyzed by flow cytometry and in all cases the main population was gated (red) first by forward scatter (FSC) and side scatter (SSC). (**K**) To measure binding of monomeric sACE2-8h proteins to myc-S expressing cells, mean fluorescence for bound protein is reported within the myc-positive gate (magenta). Surface S expression was measured by recording the mean anti-myc-Alexa 647 fluorescence signal for the entire population. (**L**) To measure binding of dimeric sACE2$_2$-IgG1 or anti-SARS-CoV-2 mAbs to myc-S expressing cells, mean fluorescence for bound protein is reported for the myc-positive gate (magenta). For all panels, representative samples are shown with percentages of cells in the gates indicated.

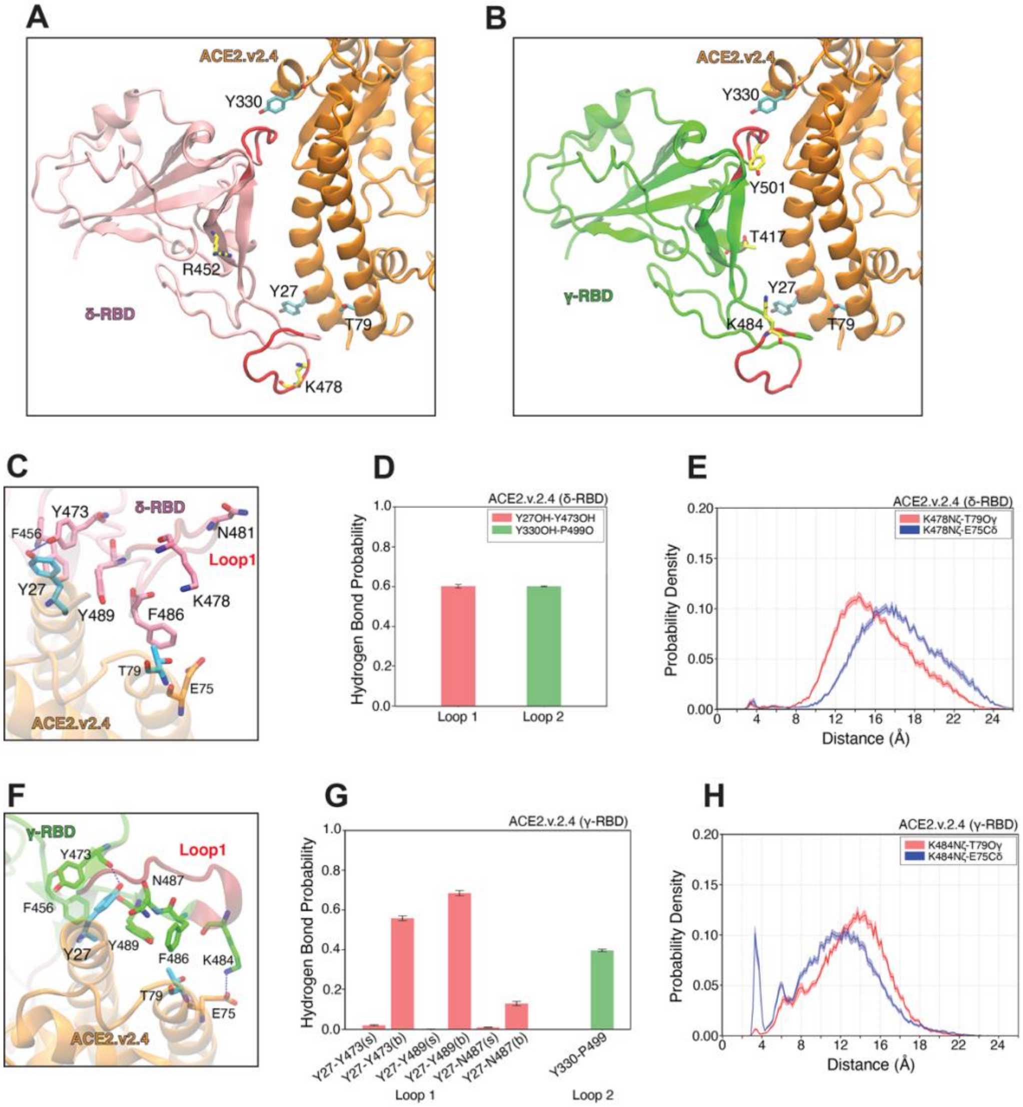

Supplementary Figure 9. Mutations in the RBDs of delta and gamma variants do not discriminate against the engineered decoy receptor. (**A-B**) ACE2.v2.4 bound to RBDs from delta and gamma VOCs were simulated (10 µs MD simulation data for each). Sites of mutations in the RBDs of (**A**) delta and (**B**) gamma compared to the original virus sequence from Wuhan, China. L452R and T478K in the delta RBD (pink ribbon) and K417T, E484K, and N501Y in the gamma RBD (green ribbon) are shown in yellow sticks. RBD loops 1 and 2 are red and ACE2.v2.4 (orange ribbon) mutations are represented as cyan sticks. (**C**) The contact region between ACE2.v2.4 (orange) and loop 1 (red) of the delta RBD (pink). (**D**) Hydrogen bond probabilities for substituted residues in v2.4 remain relatively unchanged in the complex with delta RBD (compare to Figure 2E). (**E**) Interface distances for ACE2.v2.4-T79 and -E75 with T478K in delta RBD loop 1 (this is the closest mutation in the delta RBD to the ACE2 interface). K478 in the delta VOC is relatively far away from the interface and consequently interactions made by the v2.4 mutations in ACE2 are unchanged. (**F**) The contact region between ACE2.v2.4 (orange) and loop 1 (red) of the gamma RBD (green). E484K mutation in the gamma RBD makes a salt bridge to ACE2.v2.4-E75 and facilitates changes in RBD loop 1. (**G**) New hydrogen bonds between RBD loop 1 and Y27 of ACE2.v2.4. For contacting pairs, the ACE2.v2.4 residue is listed first and the RBD residue is second. Letters ‘s’ and ‘b’ indicate side chain and backbone. (**H**) Interface distance for K484 on RBD loop 1 with ACE2.v2.4 T79 and E75. Distance distributions and hydrogen bond probabilities are from 40,000 frames selected based on the MSM stationary probability to represent the entire ensemble. Error bars represent 95% confidence intervals calculated from 20 bootstrapped samples.

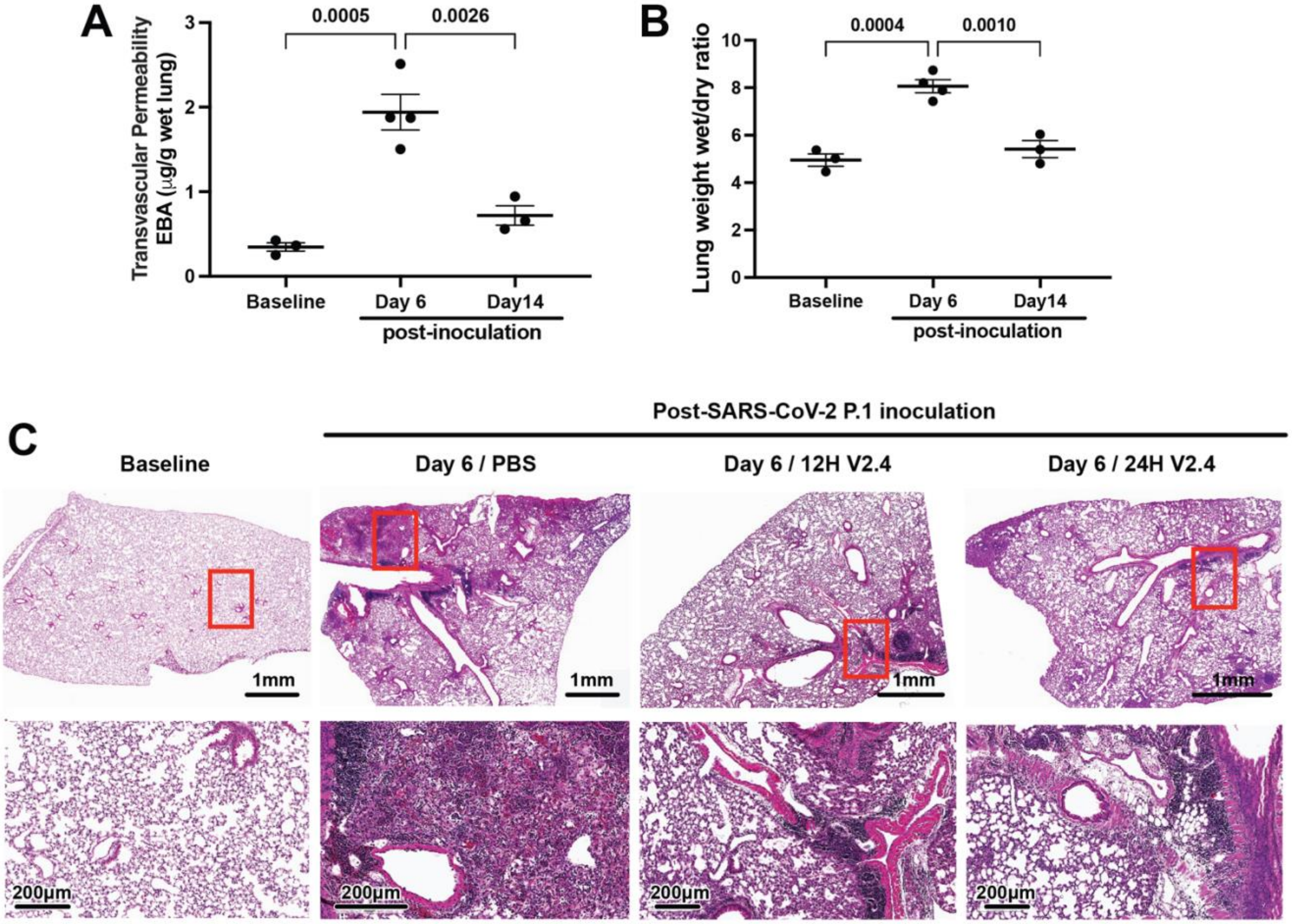

Supplementary Figure 10. sACE2$_2$.v2.4 IgG1 prevents lung vascular endothelial injury and ARDS following infection with P.1 variant of concern (Extended figures from Figure 6). (**A**-**B**) Time course of lung transvascular permeability of V2.4 12H treatment group. The EBA assay (**A**) and Wet/dry ratio (**B**) were measured at baseline, Day 6 and Day 14 post-inoculation. N=4. Data are presented by mean ± SEM and P values are shown in **A** & **B** by one-way ANOVA corrected by Tukey post-hoc test. (**C**) Representative H&E staining of lung sections at baseline (1st column), control PBS group at day 7 post-inoculation with the P.1 variant (2nd column), sACE2.V2.4-IgG 12H treatment group at day 7 (3rd column), and sACE2.V2.4-IgG 24H treatment group at day 7 (4th column) post-inoculation with the P.1 variant. The images in the first row are low magnifications. Highlighted areas (Red) are shown in higher magnification in the second row. The lungs from 4 independent mice were sectioned, stained, and imaged.

Supplementary Table 1. Toxicology: Blood cell counts and chemistry				
**sACE2$_2$.v2.4 (no fusion) (IV administered 0.5 mg/kg twice daily d0-d4, blood analyzed d7)**				
	**Vehicle (4/5 females)**	**Treated (5 females)**	**Vehicle (5 males)**	**Treated (5 males)**
Hematocrit (%)	45.8 ± 1.0 (1, 2)	46.9 ± 1.9	47.0 ± 0.9	47.0 ± 0.9
WBC (× 1000/µL)	8.4 ± 0.3	8.2 ± 1.5	8.8 ± 0.5 *	5.8 ± 0.6 *
Lymphocytes (× 1000/µL)	6.7 ± 0.2	6.5 ± 1.2	6.5 ± 0.5 *	4.1 ± 0.5 *
Neutrophils (× 1000/µL)	0.83 ± 0.06	1.1 ± 0.4	1.6 ± 0.4	1.3 ± 0.2
BUN (mg/dL)	17.2 ± 1.5	19.8 ± 0.6	19.0 ± 1.1	20.2 ± 1.0
Creatinine (mg/dL)	0.2 ± 0.0	0.2 ± 0.0	0.2 ± 0.0	0.2 ± 0.0
ALT (U/L)	24.2 ± 0.9	31.2 ± 4.3	30.6 ± 2.2	29.6 ± 3.4
**sACE2$_2$.v2.4-IgG1 (IV administered 2.0 mg/kg d0, blood analyzed d7)**				
	**Vehicle (5 females)**	**Treated (5 females)**	**Vehicle (5 males)**	**Treated (5 males)**
Hematocrit (%)	44.5 ± 1.0	45.3 ± 1.2	45.6 ± 1.5	45.3 ± 0.7
WBC (× 1000/µL)	8.4 ± 1.1	9.9 ± 0.9	8.0 ± 1.1	10.5 ± 2.1
Lymphocytes (× 1000/µL)	7.1 ± 0.8	7.7 ± 0.5	6.0 ± 1.0	7.4 ± 1.2
Neutrophils (× 1000/µL)	0.67 ± 0.12	1.4 ± 0.4	1.4 ± 0.2	2.9 ± 1.1
BUN (mg/dL)	20.2 ± 1.6	21.4 ± 1.2	21.8 ± 1.2	20.6 ± 1.6
Creatinine (mg/dL)	0.2 ± 0.0	0.2 ± 0.0	0.2 ± 0.0	0.2 ± 0.0
ALT (U/L)	31.8 ± 5.0	27.6 ± 1.4	37.6 ± 5.8	41.8 ± 4.4

(1) Statistical significance between control and treated animals was determined by independent two-tailed t-test. * indicates P < 0.05.

(2) Data are mean ± SEM.

Supplementary Tables 2. Toxicology: **Necropsy/histopathology of CD-1 mice IV administered sACE2_2.v2.4 (0.5 mg/kg twice daily d0-d4, analyzed d7)**

TREATED											
**Tissue**	**Microscopic Observation**	**Animal Identification (5 males M1-M5, 5 females F1-F5)**									
		**M1**	**M2**	**M3**	**M4**	**M5**	**F1**	**F2**	**F3**	**F4**	**F5**
Heart		0	0	0	0	0	0	0	0	0	0
Lung	Increased eosinophils, intravascular and interstitial	0	0	0	0	0	0	0	0	0	1M
	Intracellular pigment accumulation (hemosiderin)	0	1M	0	0	0	0	2M	0	1M	0
	Mixed-cell infiltrates, interstitial (histiocytes and eosinophils)	2M	0	0	0	0	1F	0	2M	1M	2M
	Mixed-cell infiltrates, interstitial with intralesional foreign material (hair)	0	2M	2M	2M	2M	0	0	0	0	0
Liver	Infiltrate, mixed-cell (histiocytes and eosinophils)	0	1F	0	0	0	0	2M	0	0	0
	Infiltrate, mixed-cell (histiocytes and eosinophils) with hepatocellular degeneration	0	0	0	0	0	2F	1M	0	0	0
	Infiltrate, mononuclear cell	1M	0	0	1M	0	1M	2M	0	1F	0
Spleen		0	0	0	0	0	0	0	0	0	0
Kidney	Infiltrate, mononuclear cell	0	0	1F	0	0	0	0	1F	0	0
	Tubular basophilia, cortical	0	0	0	0	0	0	0	0	2F	2F
Stomach		0	0	0	0	0	0	0	0	0	0
Small intestine	Mixed-cell infiltrates, mesentery (lymphocytes, histiocytes and eosinophils)	2F	0	0	0	0	0	0	0	0	0
Colon		NP	0	0	0	0	0	0	0	0	0

CONTROLS											
**Tissue**	**Microscopic Observation**	**Animal Identification (5 males M6-M10, 5 females F6-F10)**									
		**M6**	**M7**	**M8**	**M9**	**10**	**F6**	**F7**	**F8**	**F9**	**F10**
Heart		0	0	0	0	0	0	0	0	0	0
Lung	Increased eosinophils, intravascular and interstitial	0	0	0	0	0	0	0	0	0	0
	Intracellular pigment accumulation (hemosiderin)	0	0	0	0	0	0	0	0	0	0
	Mixed-cell infiltrates, interstitial (histiocytes and eosinophils)	1M	1M	1M	1M	1M	1M	1F	1M	2M	2M
	Mixed-cell infiltrates, interstitial with intralesional foreign material (hair)	1F	0	2M	0	1F	0	0	2M	1F	1F
	Osseous metaplasia	0	0	0	1F	0	0	0	0	0	0
Liver	Infiltrate, mixed-cell (histiocytes and eosinophils)	0	0	1M	0	0	1F	0	2M	2M	2M
	Infiltrate, mixed-cell (histiocytes and eosinophils) with hepatocellular degeneration	0	0	0	0	0	0	0	0	0	0
	Infiltrate, mononuclear cell	0	0	0	0	0	1F	0	0	1F	0
Spleen		0	0	0	0	0	0	0	0	0	0
Kidney	Infiltrate, mononuclear cell	0	0	1F	0	0	0	0	0	0	0
	Tubular basophilia, cortical	0	0	0	2F	0	0	0	0	0	0
Stomach		0	0	0	0	0	0	0	0	0	0
Small intestine	Mixed-cell infiltrates, mesentery (lymphocytes, histiocytes and eosinophils)	0	0	0	0	0	0	0	0	0	0
Colon		0	0	0	0	0	0	0	0	0	0

0 = histologically unremarkable; 1 = minimal; 2 = mild; 3 = moderate; 4 = marked; 5 = severe

F = foral; M = multifocal

NP = not present

**Supplementary Table 3. BLI kinetics of monovalent interactions between RBD and anti-RBD biologics (1)**						
**RBD/VOC**	**anti-RBD**	**KD (nM)**	**k_{on} ($M^{-1}s^{-1}$)**	**k_{off} (s^{-1})**	**χ^2 (2)**	**R^2 (2)**
Delta	v2.4-IgG1	0.3	8.4×10^5	2.6×10^{-4}	0.007	0.999
	VIR-7831	< 0.1	1.0×10^5	$< 1 \times 10^{-7}$	0.014	0.999
	LY-CoV555	9100	1.0×10^4	9.4×10^{-2}	0.044	0.985
	REGN10933	1.7	6.8×10^5	1.2×10^{-3}	0.290	0.993
	REGN10987	5.1	5.2×10^5	2.6×10^{-3}	0.101	0.997
Gamma	v2.4-IgG1	0.4	8.6×10^5	3.5×10^{-4}	0.013	0.998
	VIR-7831	1.2	9.7×10^4	1.2×10^{-5}	0.018	0.999
	LY-CoV555	ND (3)	ND	ND	ND	ND
	REGN10933	61	8.2×10^5	5.0×10^{-2}	0.258	0.925
	REGN10987	6.9	5.3×10^5	3.7×10^{-3}	0.125	0.995

(1) Dimeric IgG proteins were immobilized to an anti-human IgG sensor. Sensors were transferred to monomeric RBD-8h at 200, 66.7, 22.2 and 7.41 nM.

(2) χ^2 and R^2 were determined from curves fitted to the entire data.

(3) ND, not determined due to no binding.

Supplementary Table 4. Prophylaxis studies of monoclonal antibodies and ACE2 decoys.								
**Reference (DOI)**	**Manuscript Title**	**Administered Proteins**	**Animal Model**	**SAR-CoV2 Variant**	**Dose of Virus**	**Administration of Test Article [1]**	**Assessment**	**Conclusions**
This Study	Engineered High-Affinity ACE2 Peptide Mitigates ARDS and Death Induced by Multiple SARS-CoV-2 Variants	Engineered ACE2 decoy: sACE22.v2.4-IgG1	K18-hACE2 transgenic mice	USA-WA1/2020	10^4 PFU intranasally	10 mg/kg i.v. 12 h prior to infection	Daily weight loss, daily survival, lung vascular leakage and edema at d 7	Animals were fully protected.
10.1101/2020.06.15.152157	Novel ACE2-IgG1 fusions with improved in vitro and in vivo activity against SARS-CoV2	Wild type or catalytically inactive sACE2-IgG1(LALA)	AdV-hACE2 transduced mice	Unspecified. Virus isolate is from early 2020.	5 × 10^4 to 2 × 10^5 PFU intranasally	15 mg/kg i.v. 4 h prior to infection	Viral load in lungs on d 3	Wild type sACE2-IgG1 had no effect. Catalytically inactive sACE2-IgG1 with slightly increased SARS-CoV-2 affinity caused moderate decrease in virus load.
10.1371/journal.ppat.1009544	Intranasal gene therapy to prevent infection by SARS-CoV-2 variants	Engineered ACE2 decoy: CDY14HL-Fc4	K18-hACE2 transgenic mice	USA-WA1/2020	2.8 × 10^2 PFU intranasally	Gene therapy: i.n. delivery of AAV expressing CDY14HL-Fc4 7 days prior to infection	Daily weight loss, viral RNA and histopathology on days 4 and 7	Small to moderate reductions in weight loss, viral RNA levels, and lung pathology.
10.1038/s41586-021-03720-y	In vivo monoclonal antibody efficacy against SARS-CoV-2 variant strains	mAbs: 2B04+47D11, S309+S2E12, COV2-2130+COV2-2196, REGN10933+REGN10987, or LY-CoV555	K18-hACE2 transgenic mice	WA1/2020 N501Y/D614G, B.1.1.7, Wash-B.1.351 and Wash-B.1.1.28	10^3 FFU intranasally	0.2 mg/kg and 2 mg/kg i.p. 24 h prior to infection	Daily weight loss, viral loads in tissues at day 3 or 6, cytokine analysis	Limited, variant-specific protection by 2B04+47D11 and LY-CoV555. Reductions in viral RNA were diminished for B.1.351 virus, especially for REGN10933+REGN10987 and COV2-2130+COV2-2196. Overall, cocktails were protective.
10.1038/s41586-021-03720-y	In vivo monoclonal antibody efficacy against SARS-CoV-2 variant strains	mAbs: COV2-2130+COV2-2196	Syrian hamster	Wash-B.1.351 and WA1/2020 D614G	5 × 10^5 FFU intranasally	4 mg/kg and 10 mg/kg i.p. 24 h prior to infection	Daily weight loss, viral loads in tissues at day 4, cytokine analysis	Animals were protected. Viral RNA remained high in nasal washes.
10.1084/jem.20201993	Antibody potency, effector function and combinations in protection from SARS-CoV-2 infection in vivo	mAbs: C002, C104, C105, C110, C119, C121-LS, C135-LS, and C144-LS	Wild type mice	SARS-CoV-2 MA	10^5 PFU intranasally	8mg/kg i.p. 12 h prior to infection of individual mAbs, and 16 / 5.3 / 1.8 mg/kg i.p. for cocktails (C135-LS/C121-LS and C135-LS/C144-LS)	Viral load in lungs at day 2. This is a non-lethal infection model.	Prophylactic efficacy in vivo did not correlate well with in vitro neutralization and required effector functions. Cocktail can out-perform monotherapy at a lower dose (5.3 mg/kg). Protection was partial at the lowest dose of 1.8 mg/kg.
10.1084/jem.20201993	Antibody potency, effector function and combinations in protection from SARS-CoV-2 infection in vivo	mAbs: C135-LS+C144-LS	Syrian hamster	Unspecified	2.6 × 10^4 PFU intranasally	20 / 6 / 2 mg/kg i.p. 24 h prior to infection	Viral load in lungs at d 3.	All doses substantially reduced viral load at d 3.
10.1038/s41586-020-2548-6	Potently neutralizing and protective human antibodies against SARS-CoV-2	mAbs: COV2-2196, COV2-2130, and COV2-2196+COV2-2130	AdV-hACE2 transduced mice	USA_WA1/2020	4 × 10^5 PFU intranasally	10 mg/kg i.p. 24 prior to infection	Daily weight loss, histopathology at day 7, viral RNA at day 7, inflammatory cytokines at day 7	Partial protection from weight loss and infection.
10.1038/s41586-020-2548-6	Potently neutralizing and protective human antibodies against SARS-CoV-2	mAbs: COV2-2196, COV2-2130, and COV2-2196+COV2-2130	Wild type mice	SARS-CoV-2 MA	10^5 PFU intranasally	10 mg/kg i.p. 6 h prior to infection	Viral RNA in lungs at d 2	Reduction in virus replication
10.1038/s41586-020-2548-6	Potently neutralizing and protective human antibodies against SARS-CoV-2	mAbs: COV2-2196 or COV2-2130	Rhesus macaque	USA_WA1/2020	1.1 × 10^4 PFU intranasally and intratracheally	50 mg/kg i.v. 3 days prior to infection	Viral RNA in bronchoalveolar lavage and nasal swabs	Reduction in virus replication
10.1038/s41586-020-2381-y	A human neutralizing antibody targets the receptor-binding site of SARS-CoV-2	mAb: CB6	Rhesus macaques	BetaCoV/Wuhan/IVDC-HB-envF13/2020	10^5 TCID50 intratracheally	50 mg/kg i.v. 24 h prior to infection	Viral RNA in throat swabs, histopathology at day 5	Blocked virus replication
10.1101/2021.03.09.434607	The dual function monoclonal antibodies VIR-7831 and VIR-7832 demonstrate potent in vitro and in vivo activity against SARS-CoV-2	mAb: VIR-7831	Syrian hamster	USA-WA1/2020	7.4 × 10^4 TCID50 intranasally	30 / 5 / 0.5 / 0.05 mg/kg 24 h or 15 / 5 / 0.5 / 0.05 mg/kg 48 h i.p. prior to infection	Lung viral load at day 4, daily body weight change	VIR-7831 decreased viral load and ameliorated weight loss. Efficacy dropped substantially at doses less than 5 mg/kg.
10.4049/jimmunol.2000583	A Potently Neutralizing Antibody Protects Mice against SARS-CoV-2 Infection	Murine mAb: 2B04	AdV-hACE2 transduced mice	USA-WA1/2020	4 × 10^5 FFU intranasally	10 mg/kg 2B04 i.p. 24 h prior to infection	Daily weight, histopathology and viral load on day 4	Ameliorated weight loss and reduced viral load
10.1126/scitranslmed.abf1906	The neutralizing antibody, LY-CoV555, protects against SARS-CoV-2 infection in nonhuman primates	mAb: LY-CoV555	Rhesus macaque	USA-WA1/2020	1.1 × 10^5 PFU intranasally and intratracheally	1 / 2.5 / 15 / 50 mg/kg i.v. 24 h prior to infection	Viral RNA in nasal and throat swabs and in lungs	All doses reduced viral RNA, especially at 2.5 mg/kg and higher.
10.1016/j.cell.2020.05.025	Potent Neutralizing Antibodies against SARS-CoV-2 Identified by High-Throughput Single-Cell Sequencing of Convalescent Patients' B Cells	mAb: BD-368-2	hACE2 transgenic mice	Wuhan/AMMS01/2020	10^5 TCID50 intranasally	20 mg/kg i.p. 24 h prior to infection	Daily weight loss, viral RNA in lungs on day 5	Ameliorated weight loss and blocked virus replication
10.1126/science.abe2402	REGN-COV2 antibodies prevent and treat SARS-CoV-2 infection in rhesus macaques and hamsters	mAb cocktail: REGN10933+REGN10987	Rhesus macaque	USA-WA1/2020	10^5 or 1.05 × 10^6 PFU intranasally and intratracheally	0.3 / 50 mg/kg i.v. 72 h prior to infection	Viral RNA in nasal and throat swabs and BAL over 8 days, histopathology	50 mg/kg dose was effective, 0.3 mg/kg was not.
10.1126/science.abe2402	REGN-COV2 antibodies prevent and treat SARS-CoV-2 infection in rhesus macaques and hamsters	mAb cocktail: REGN10933+REGN10987	Syrian hamster	USA-WA1/2020	2.3 × 10^4 PFU intranasally	50 / 5 / 0.5 mg/kg i.v. 48 h prior to infection	Daily weight loss, viral RNA in lungs and histopathology on day 7	All doses prevented weight loss, reduced viral load and reduced severity of lung damage
10.1126/science.abf4830	Broad and potent activity against SARS-like viruses by an engineered human monoclonal antibody	mAb: ADG-2	Wild type mice	SARS-CoV-MA15 or SARS2-CoV-2-MA10	10^3 PFU intranasally	10 mg/kg i.p. 12 h prior to infection	Daily weight loss, lung function and injury, lung viral load at days 2 and 4	Prophylaxis completely protected mice

[1] In cases where mice were administered a defined mass of test article, mouse weight is approximated to be 20 g for calculating a dose in mg/kg.

Supplementary Table 5. Therapeutic studies of monoclonal antibodies and ACE2 decoys.								
**Reference (DOI)**	**Manuscript Title**	**Administered Proteins**	**Animal Model**	**SAR-CoV2 Variant**	**Dose of Virus**	**Administration of Test Article** [1]	**Assessment**	**Conclusions**
This Study	Engineered High-Affinity ACE2 Peptide Mitigates ARDS and Death Induced by Multiple SARS-CoV-2 Variants	Engineered ACE2 decoy: sACE22.v2.4-IgG1	K18-hACE2 transgenic mice	USA-WA1/2020 and Japan/TY7-503/2021 (P.1 variant)	10^4 PFU intranasally	10 mg/kg i.v. daily for 7 days beginning 12 h post-infection, or 15 mg/kg i.v. daily for 7 days beginning 24 h post infection,	Daily weight loss, daily survival, lung vascular leakage, edema and histopathology at days 6 or 7 and 14, viral load in lungs on day 6 or 7	Animals were partially protected from weight loss, death, vascular leakage, and lung pathology. Viral burden substantially reduced. Animals fully recovered in 14 days. P.1 virus had a faster disease course and required treatment to begin at 12 h for survival, although later treatment still reduced lung vascular leakage, edema, and viral burden.
10.1038/s41467-021-24013-y	Engineered ACE2 receptor therapy overcomes mutational escape of SARS-CoV-2	Engineered ACE2 decoy: 3N39v2-Fc	Syrian hamster	Japan/TY/WK-521/2020	10^6 PFU intranasally	20 mg/kg i.p. 2 h post infection, smaller study where administered 48 hours post infection	Daily body weight, on day 5 assessment of viral load, inflammatory gene expression, and histopathology	Treated (2 h post-infection) animals recovered in body weight by day 5 and had reduced inflammatory gene expression and viral load. Treatment 2 days post-infection gave smaller decreases in viral load and inflammatory gene expression, no data provided on weight loss.
10.1038/s41586-021-03720-y	In vivo monoclonal antibody efficacy against SARS-CoV-2 variant strains	mAbs: 2B04+47D11, S309+S2E12, COV2-2130+COV2-2196, REGN10933+REGN10987, LY-CoV555+LY-CoV016, or LY-CoV555	K18-hACE2 transgenic mice	WA1/2020 N501Y/D614G and Wash-B.1.351	10^3 FFU intranasally	10 mg/kg i.p. 24 h post infection	Daily weight loss, viral loads in tissues at day 6, histopathology	Except for treatment with LY-CoV555 or LY-CoV555+LY-CoV016 in B.1.351 infected mice, animals were protected. Unlike prophylaxis, in vitro neutralization efficacy did not correlate well with therapeutic efficacy, indicating the importance of other effector functions.
10.1084/jem.20201993	Antibody potency, effector function and combinations in protection from SARS-CoV-2 infection in vivo	mAbs: C135-LS+C144-LS	Syrian hamster	Unspecified	2.6 × 10^4 PFU intranasally	40 / 12 / 4 mg/kg i.p. 12 h post infection	Viral load in lungs at d 3.	All doses substantially reduced viral load at d 3.
10.1038/s41586-020-2548-6	Potently neutralizing and protective human antibodies against SARS-CoV-2	mAbs: COV2-2196+COV2-2130	AdV-hACE2 transduced mice	USA_WA1/2020	4 × 10^5 PFU intranasally	20 mg/kg i.p. 12 h post infection	Viral RNA in lungs at day 2, inflammatory cytokines	Reduction in virus replication and inflammatory cytokines
10.1038/s41586-020-2548-6	Potently neutralizing and protective human antibodies against SARS-CoV-2	mAbs: COV2-2196, COV2-2130, and COV2-2196+COV2-2130	Wild type mice	SARS-CoV-2 MA	10^5 PFU intranasally	20 mg/kg i.p. 12 h post infection	Viral RNA in lungs at d 2	Reduction in virus replication
10.1038/s41467-020-20602-5	A therapeutic neutralizing antibody targeting receptor binding domain of SARS-CoV-2 spike protein	mAb: CT-P59	Ferret	NMC-nCoV02	10^5.5 - 10^5.8 TCID50 intranasally and intratracheally	3 / 30 mg/kg i.v. 24 h post infection	Viral load in nasal washes, histopathology at days 3 and 7	30 mg/kg dose reduced viral load. Lower dose much less efficacious.
10.1038/s41467-020-20602-5	A therapeutic neutralizing antibody targeting receptor binding domain of SARS-CoV-2 spike protein	mAb: CT-P59	Syrian hamster	NMC-nCoV02	6.4 × 10^4 PFU intranasally	15 / 30 / 60 / 90 mg/kg i.p. 24 h post infection	Viral load at days 3 and 5	Reduced virus replication. Less efficacious at lowest dose (15 mg/kg)
10.1038/s41467-020-20602-5	A therapeutic neutralizing antibody targeting receptor binding domain of SARS-CoV-2 spike protein	mAb: CT-P59	Rhesus macaque	NMC-nCoV02	6.4 × 10^4 PFU intranasally	45 / 90 mg/kg i.v. 24 h post infection	Viral load in nasal and throat swabs and in lungs	Reduced virus replication in upper respiratory tract but no change in viral RNA in lungs.
10.1038/s41586-020-2381-y	A human neutralizing antibody targets the receptor-binding site of SARS-CoV-2	mAb: CB6	Rhesus macaques	BetaCoV/Wuhan/IVDC-HB-envF13/2020	10^5 TCID50 intratracheally	50 mg/kg i.v. administered twice (days 1 and 3) post infection	Viral RNA in throat swabs, histopathology at day 5	Reduced virus replication
10.1016/j.cell.2020.05.025	Potent Neutralizing Antibodies against SARS-CoV-2 Identified by High-Throughput Single-Cell Sequencing of Convalescent Patients' B Cells	mAb: BD-368-2	hACE2 transgenic mice	Wuhan/AMMS01/2020	10^5 TCID50 intranasally	20 mg/kg i.p. 2 h post infection	Daily weight loss, viral RNA in lungs on day 5	Ameliorated weight loss and reduced virus replication
10.1126/science.abe2402	REGN-COV2 antibodies prevent and treat SARS-CoV-2 infection in rhesus macaques and hamsters	mAb cocktail: REGN10933+REGN10987	Rhesus macaque	USA-WA1/2020	10^6 PFU intranasally and intratracheally	25 / 150 mg/kg i.v. 24 h post infection	Viral RNA in nasal and throat swabs and BAL over 7 days, histopathology	Partially effective at reducing viral load.
10.1126/science.abe2402	REGN-COV2 antibodies prevent and treat SARS-CoV-2 infection in rhesus macaques and hamsters	mAb cocktail: REGN10933+REGN10987	Syrian hamster	USA-WA1/2020	2.3 × 10^4 PFU intranasally	50 / 5 / 0.5 mg/kg i.v. 24 h post infection	Daily weight loss	5 and 50 mg/kg doses ameliorated weight loss
10.1126/science.abf4830	Broad and potent activity against SARS-like viruses by an engineered human monoclonal antibody	mAb: ADG-2	Wild type mice	SARS2-CoV-2-MA10	10^3 PFU intranasally	10 mg/kg i.p. 12 h post infection	Daily weight loss, lung function and injury, lung viral load at days 2 and 4	Partial amelioration of pathology of varying significance, virus levels reduced at day 4

[1] In cases where mice were administered a defined mass of test article, mouse weight is approximated to be 20 g for calculating a dose in mg/kg.

Supplementary Table 6. Primer sequences			
**qPCR primer sequences**			
Target gene	Forward primer	Reverse primer	Size
SARS- CoV-2 Spike	GCTGGTGCTGCAGCTTATTA	AGGGTCAAGTGCACAGTCTA	105
SARS- CoV-2 NSP	CAATGCTGCAATCGTGCTAC	GTTGCGACTACGTGATGAGG	110
Cdh5	GTCGATGCTAACACAGGGAATG	AATACCTGGTGCGAAAACACA	185
PPIA	GGCAAATGCTGGACCAAACAC	TTCCTGGACCCAAAACGCTC	147
Human TMPRSS2	AATCGGTGTGTTCGCCTCTAC	CGTAGTTCTCGTTCCAGTCGT	106
Human ACE-2	GGGATCAGAGATCGGAAGAAGAAA	AGGAGGTCTGAACATCATCAGTG	124
**RT-PCR primer sequences**			
Human ACE-2	ACAGTCCACACTTGCCCAAA	ATCTCCAGCTTTCCCAAGCC	284
Human TMPRSS2	TGAAAGCGGGTGTGAGGAGC	GGGTCAAGGTGATGCACAGT	189
