# Supplementary material for: Engineered ACE2 decoy mitigates lung injury and death induced by SARS-CoV-2 variants
Source: Nat Chem Biol. 2022 Jan 19;18(3):342–51. doi: 10.1038/s41589-021-00965-6 (PMC8885411; doi:10.1038/s41589-021-00965-6)
Supplement: Supplementary file 2 — Reporting Summary [file 41589_2021_965_MOESM2_ESM.pdf]

nature research

Corresponding author(s): Asrar B. Malik; Jalees Rehman, Erik Procko, et al.

Last updated by author(s): 08/30/2021

Reporting Summary

Nature Research wishes to improve the reproducibility of the work that we publish. This form provides structure for consistency and transparency in reporting. For further information on Nature Research policies, see our Editorial Policies and the Editorial Policy Checklist.

Statistics

For all statistical analyses, confirm that the following items are present in the figure legend, table legend, main text, or Methods section.

n/a	Confirmed	
☐	☒	The exact sample size (*n*) for each experimental group/condition, given as a discrete number and unit of measurement
☐	☒	A statement on whether measurements were taken from distinct samples or whether the same sample was measured repeatedly
☐	☒	The statistical test(s) used AND whether they are one- or two-sided<br>*Only common tests should be described solely by name; describe more complex techniques in the Methods section.*
☒	☐	A description of all covariates tested
☐	☒	A description of any assumptions or corrections, such as tests of normality and adjustment for multiple comparisons
☐	☒	A full description of the statistical parameters including central tendency (e.g. means) or other basic estimates (e.g. regression coefficient) AND variation (e.g. standard deviation) or associated estimates of uncertainty (e.g. confidence intervals)
☐	☒	For null hypothesis testing, the test statistic (e.g. *F*, *t*, *r*) with confidence intervals, effect sizes, degrees of freedom and *P* value noted<br>*Give P values as exact values whenever suitable.*
☒	☐	For Bayesian analysis, information on the choice of priors and Markov chain Monte Carlo settings
☒	☐	For hierarchical and complex designs, identification of the appropriate level for tests and full reporting of outcomes
☐	☒	Estimates of effect sizes (e.g. Cohen's *d*, Pearson's *r*), indicating how they were calculated

Our web collection on statistics for biologists contains articles on many of the points above.

Software and code

Policy information about availability of computer code

Data collection	For flow cytometry assays to determine binding between ACE2 and S, data were collected on a BD Accuri using the instrument software. Data were analyzed (i.e. gates were drawn and mean fluorescence determined) using the instrument software or using FCS Express version 6 (De Novo Software).
Data analysis	Flow cytometry data were analyzed using the BD Accuri instrument software. ELISA absorbance and ACE2 fluorescent substrate measurements were exported for analysis in Excel version 16.50 (Microsoft). Source code for TLmutation is available in GitHub (https://github.com/ShuklaGroup/TLMutation). Citations are provided for the variant effect predictors EVcoupling, Envision and SNAP2. Web servers for variant effect predictors were accessed between 10/2020 and 3/2021. All software for MD simulations and analysis and described in Methods. Prism 9 was used to analyze the data from mouse experiments.

For manuscripts utilizing custom algorithms or software that are central to the research but not yet described in published literature, software must be made available to editors and reviewers. We strongly encourage code deposition in a community repository (e.g. GitHub). See the Nature Research guidelines for submitting code & software for further information.

Data

Policy information about availability of data

All manuscripts must include a data availability statement. This statement should provide the following information, where applicable:

- Accession codes, unique identifiers, or web links for publicly available datasets
- A list of figures that have associated raw data
- A description of any restrictions on data availability

This research did not generate new sequence data or experimental structure coordinate data. Molecular dynamics data with features for MSM building and an

embedded Box link are deposited on GitHub (https://github.com/ShuklaGroup). Other data generated during the current study are available from the corresponding authors on reasonable request.

Field-specific reporting

Please select the one below that is the best fit for your research. If you are not sure, read the appropriate sections before making your selection.

☒ Life sciences ☐ Behavioural & social sciences ☐ Ecological, evolutionary & environmental sciences

For a reference copy of the document with all sections, see nature.com/documents/nr-reporting-summary-flat.pdf

Life sciences study design

All studies must disclose on these points even when the disclosure is negative.

Sample size	Sample sizes were chosen based on standards in the field for equivalent experiments.<br>For mouse experiments, the sample sizes were determined according to our previous published experiments. A 2-sided power analysis based on the MGH Dept of Statistics power calculator, http://hedwig.mgh.harvard.edu/sample_size/quan_measur/para_quant.html, based on the following: an alpha level of 0.05,and power of 0.8, assuming a minimal detectable difference between means of 2 x the standard deviation.
Data exclusions	No data were excluded.
Replication	Experiments were replicated as described in the manuscript.
Randomization	For mouse experiments, the same number of male and female mice were allocated into each experimental groups randomly.
Blinding	Blinding was not possible in the BSL3 facility. Animal research under BSL3 containment is tightly regulated and limited to specially trained researchers. The mice experiments were age and sex-controlled. The cell culture experiments were passage-controlled for primary cells. The mouse housing and cell culturing were controlled for the same conditions for each experiment groups.

Reporting for specific materials, systems and methods

We require information from authors about some types of materials, experimental systems and methods used in many studies. Here, indicate whether each material, system or method listed is relevant to your study. If you are not sure if a list item applies to your research, read the appropriate section before selecting a response.

Materials & experimental systems

n/a	Involved in the study	
☐	☒	Antibodies
☐	☒	Eukaryotic cell lines
☒	☐	Palaeontology and archaeology
☐	☒	Animals and other organisms
☒	☐	Human research participants
☒	☐	Clinical data
☒	☐	Dual use research of concern

Methods

n/a	Involved in the study	
☒	☐	ChIP-seq
☐	☒	Flow cytometry
☒	☐	MRI-based neuroimaging

Antibodies

Antibodies used	Peroxidase AffiniPure Donkey Anti-Human IgG, Fcγ fragment specific (Jackson Immuno Research, Cat. 709-035-098)<br>anti-human ACE2 Mouse Monoclonal (HRP conjugated) (clone OTI1D2, Origene, Cat. TA803842BM)<br>anti-myc Alexa 647 (clone 9B11, Cell Signaling Technology, Cat. 2233S)<br>anti-HIS-FITC (chicken polyclonal, Immunology Consultants Laboratory, Cat. CHIS-45F)<br>anti-human IgG-APC (clone M1310G05, BioLegend, Cat. 410712)<br>anti-CD45-EF450 (eBioscience #48-0451-82);<br>anti-CD31-APC (eBioscience #17-0311-82)<br>anti-MYC-FITC (chicken polyclonal, Immunology Consultants Laboratory, Cat. CMYC-45F)<br>REGN10933 (prepared in the Procko lab)<br>REGN10987 (prepared in the Procko lab)<br>VIR-7831 (prepared in the Procko lab)<br>LY-CoV555 (prepared in the Procko lab)
Validation	All antibodies are commercially supplied and validated by the vendors, with the exception of monoclonal antibodies with EUA for clinical use in early COVID. For these anti-SARS-CoV-2 mAbs, sequences were pulled from the KEGG database (Accession No. REGN10933, D11938; REGN10987, D11939; VIR-7831, D12014; LY-CoV555, D11936). H and L chains were synthesized with CD5 leader sequences as synthetic, human codon-optimized genes and cloned into pcDNA3.1(+) for expression and purification from Expi293F culture. Protein concentrations were determined by UV (280 nm) absorbance using calculated extinction coefficients

(excluding glycans). The mAbs were highly active for binding SARS-CoV-2 spikes as shown in Figure 6B. Relative binding of the mAbs to the different Spike variants correlates with rank order in neutralization experiments as detailed in the S-1 public filing by Adagio Therapeutics, Inc: see page 128 in document https://sec.report/Document/0001193125-21-217506. The activity of the purified mAbs therefore matches public reports and expectations.

Eukaryotic cell lines

Policy information about cell lines

Cell line source(s)	Expi293F (ThermoFisher), ExpiCHO-S (ThermoFisher),Vero E6 (ATCC, CRL-1586); Vero CCL81 (ATCC); ACE-2 expressing A549 cells and standard A549 cells (ATCC, CRM-CCL-185), Primary human lung microvascular endothelial cells (HLMECs, Lonza).
Authentication	Cell lines were purchased from ThermoFisher, ATCC, or Lonza which routinely authenticate their cell lines. We also authenticated expression of ACE2 expression on ACE-2 A549 cells.
Mycoplasma contamination	Expi293F, Vero E6, Vero CCL81, ACE-2-A549, A549, cells and HLMECs were tested for mycoplasma and were negative. ExpiCHO-S cells were not tested.
Commonly misidentified lines (See ICLAC register)	*Name any commonly misidentified cell lines used in the study and provide a rationale for their use.*

Animals and other organisms

Policy information about studies involving animals; ARRIVE guidelines recommended for reporting animal research

Laboratory animals	For SARS-CoV-2 mouse experiments, the K18-hACE2 transgenic mice (The Jackson Laboratory, Stock No: 034860) express human ACE2 driven by the K18 promoter and is required for cellular entry of SAR-CoV-2.<br>For pharmacokinetics studies, soluble ACE2 proteins were administered as described in the Methods to 8-week-old CD-1 IGS mice and 8-10 week old C57BL/6 mice.
Wild animals	No wild animals were used.
Field-collected samples	No field-collected samples were used.
Ethics oversight	For SARS-CoV-2 mouse experiments, all aspects were approved by the office of Environmental Health and Safety at University of Illinois at Chicago prior to the initiation of this study. Working with SARS-CoV-2 was performed in a BSL-3 laboratory by personnel equipped with powered air purifying respirators.<br>PK studies at the UIC Toxicology Research Laboratory (UIC/TRL Study No. 845) were approved by the UIC Animal Care Committee. PK and toxicology studies at UIUC Department of Veterinary Clinical Medicine were approved by UIUC Institutional Animal Care and Use Committee (UIUC protocol 20127).

Note that full information on the approval of the study protocol must also be provided in the manuscript.

Flow Cytometry

Plots

Confirm that:

- [x] The axis labels state the marker and fluorochrome used (e.g. CD4-FITC).
- [x] The axis scales are clearly visible. Include numbers along axes only for bottom left plot of group (a 'group' is an analysis of identical markers).
- [x] All plots are contour plots with outliers or pseudocolor plots.
- [x] A numerical value for number of cells or percentage (with statistics) is provided.

Methodology

Sample preparation	For isolating mouse lung cells, the mouse lung tissue was minced and digested with 3 ml Collagenase A (Roche, Cat: 10103586001, 1 mg/ml in PBS) at 37ºC water bath for 1 hour. Mixtures were titrated with #18 gauge needles and then pipetted through a 40 µm disposable cell strainer. After centrifuging 300 g for 5 minutes and washing with PBS, isolated cells were treated with red blood cell lysis buffer (eBioscience, Cat:00-4300-54) for 5 minutes to lyse red blood cells. After isolation, remaining cells were incubated with anti-mouse CD16/CD32 (1:50, BD Pharmingen#553142) to block endogenous Fc for 10 minutes on ice. After this, cells were stained with antibodies including CD45-EF450 (1:200, eBioscience #48-0451-82) and CD31-APC (1:100, eBioscience #17-0311-82) for 45 minutes at 4°C. After wash, the cells were resuspended in 500 µl buffer.<br>To analyze Spike/ACE2 interactions, Expi293F cells were transfected with full-length, transmembrane Spike or ACE2, washed post-transfection with PBS containing 0.2% BSA, and then stained with soluble ACE2, SARS-CoV-2 RBD, or monoclonal antibodies as described fully in Methods.
Instrument	BD Accuri was applied for cultured cell experiments. LSR Fortessa (BD Pharmingen, SN: H6477940000024) cell cytometer was applied for mouse lung cell experiments.

Software	BD Accuri instrument software was used to analyze cultured cell experiments. Kaluza software from Beckman Coulter was used to analyze mouse lung cell experiments
Cell population abundance	Transfected Expi293F cells expressing either ACE2 or SARS-CoV-2 Spike were analyzed (a single cell population constituting the entire sample) but not sorted.
Gating strategy	For analysis of transfected Expi293F cells, the main cell population was gated by FSC/SSC to remove debris. To assess the binding to Spike of soluble ACE2 or monoclonal antibodies, cells positive for the myc tag on S after staining with fluorescent anti-myc antibody were gated based on a negative control (the positive gate was set to <0.5% of mock transfected cells); mean fluorescence for bound sACE2 or monoclonal antibodies within the myc-positive gate were then determined. To assess surface levels of S on transfected Expi293F, the mean fluorescence signal for the myc tag was measured for the entire FSC/SSC-gated cell population.<br>The gating strategy used for the mouse lung cells involved removing cell debris and doublets or clumps.

☒ Tick this box to confirm that a figure exemplifying the gating strategy is provided in the Supplementary Information.
