# Supplementary figures and images for: Engineered ACE2 decoy mitigates lung injury and death induced by SARS-CoV-2 variants
Source: Nat Chem Biol. 2022 Jan 19;18(3):342–51. doi: 10.1038/s41589-021-00965-6 (PMC8885411; doi:10.1038/s41589-021-00965-6)

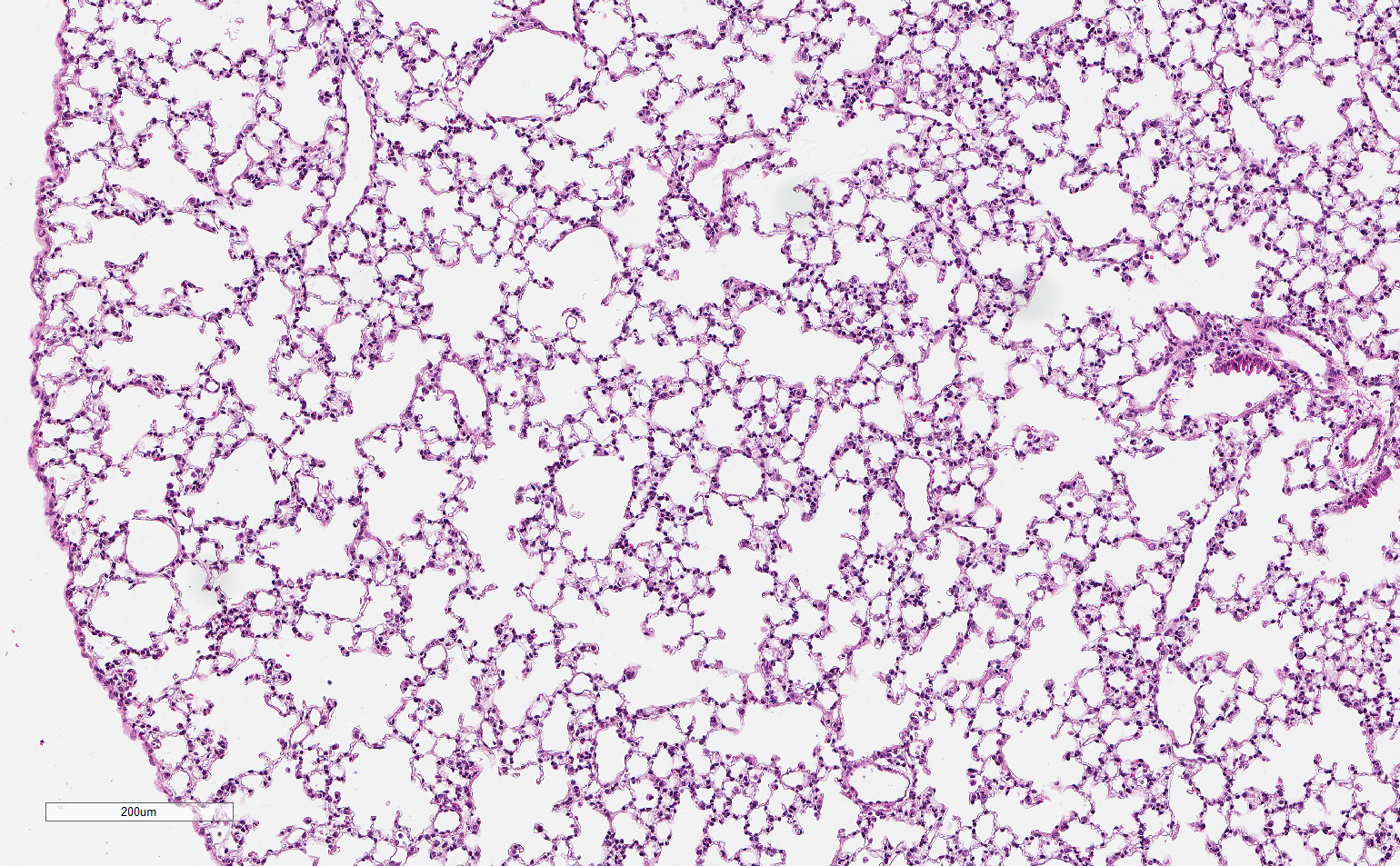

Supplement: Supplementary file 3 — Uncropped images and numerical data for Supplementary Fig. 1. [file 41589_2021_965_MOESM3_ESM.zip › Source_data_for_Supplementary_Figure_1/Uncropped images for Supplemental Figure 1D/Supplementary Figure 1D -r1c2.tif]

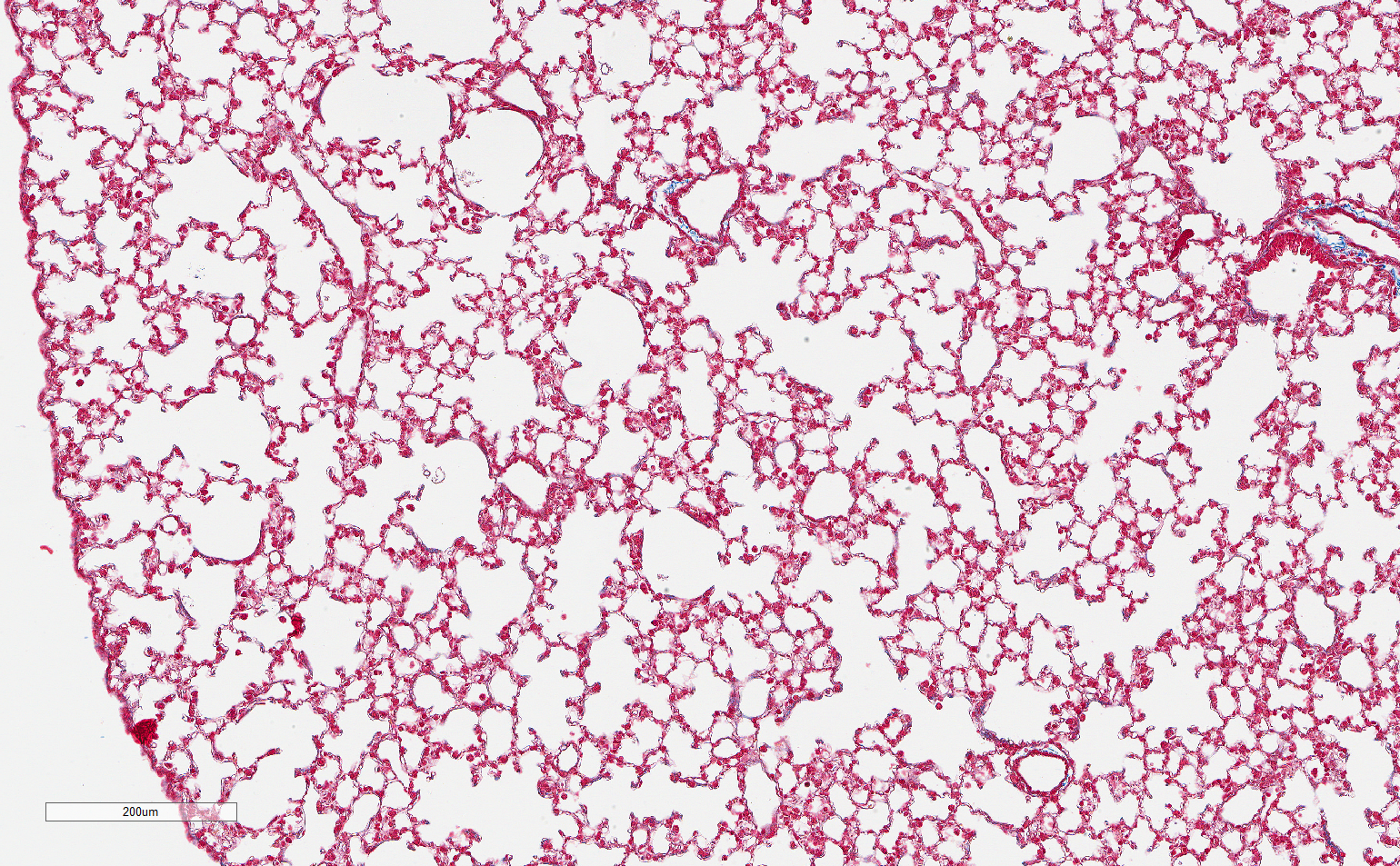

Supplement: Supplementary file 3 — Uncropped images and numerical data for Supplementary Fig. 1. [file 41589_2021_965_MOESM3_ESM.zip › Source_data_for_Supplementary_Figure_1/Uncropped images for Supplemental Figure 1D/Supplementary Figure 1D -r1c3.tif]

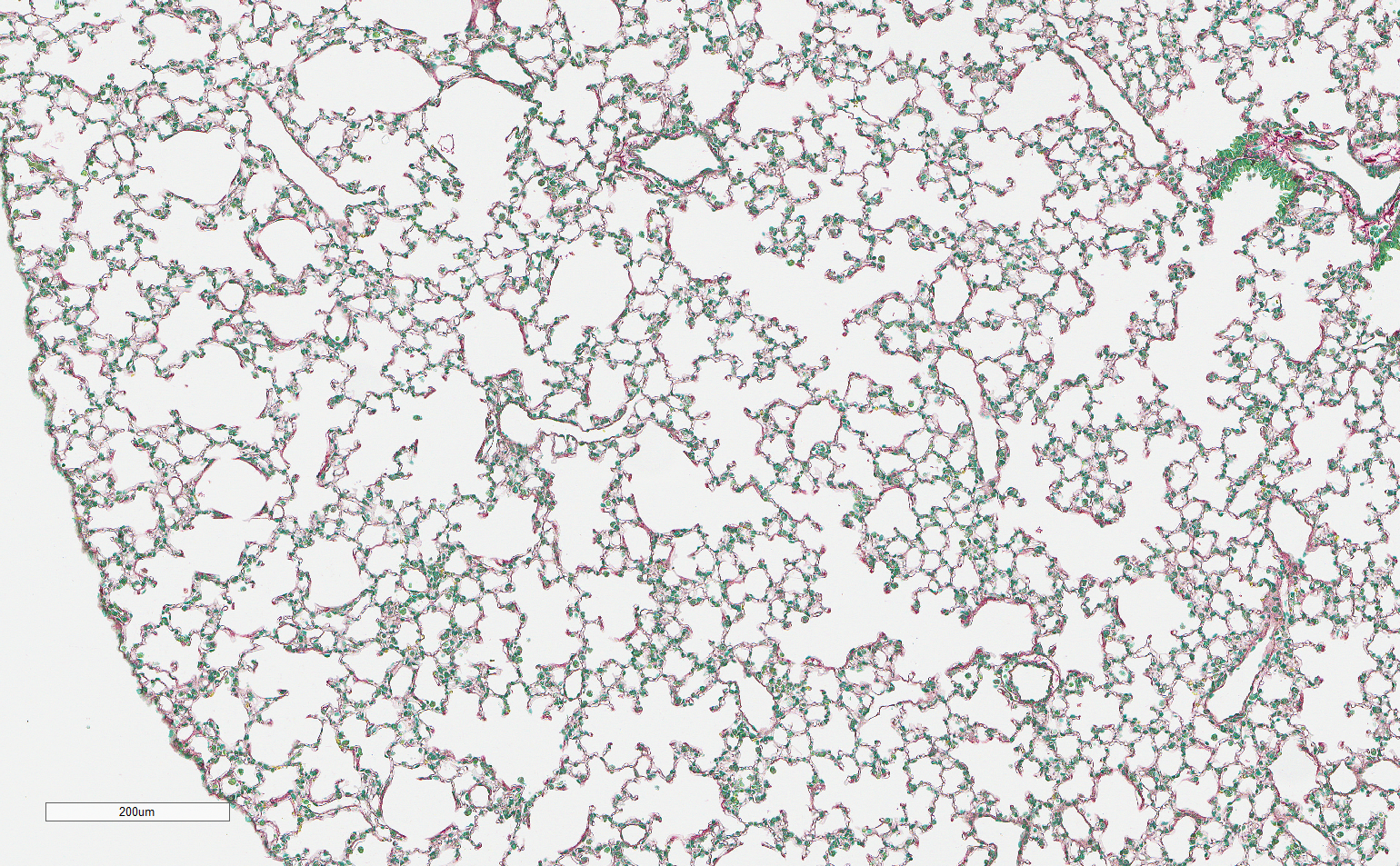

Supplement: Supplementary file 3 — Uncropped images and numerical data for Supplementary Fig. 1. [file 41589_2021_965_MOESM3_ESM.zip › Source_data_for_Supplementary_Figure_1/Uncropped images for Supplemental Figure 1D/Supplementary Figure 1D -r1c4.tif]

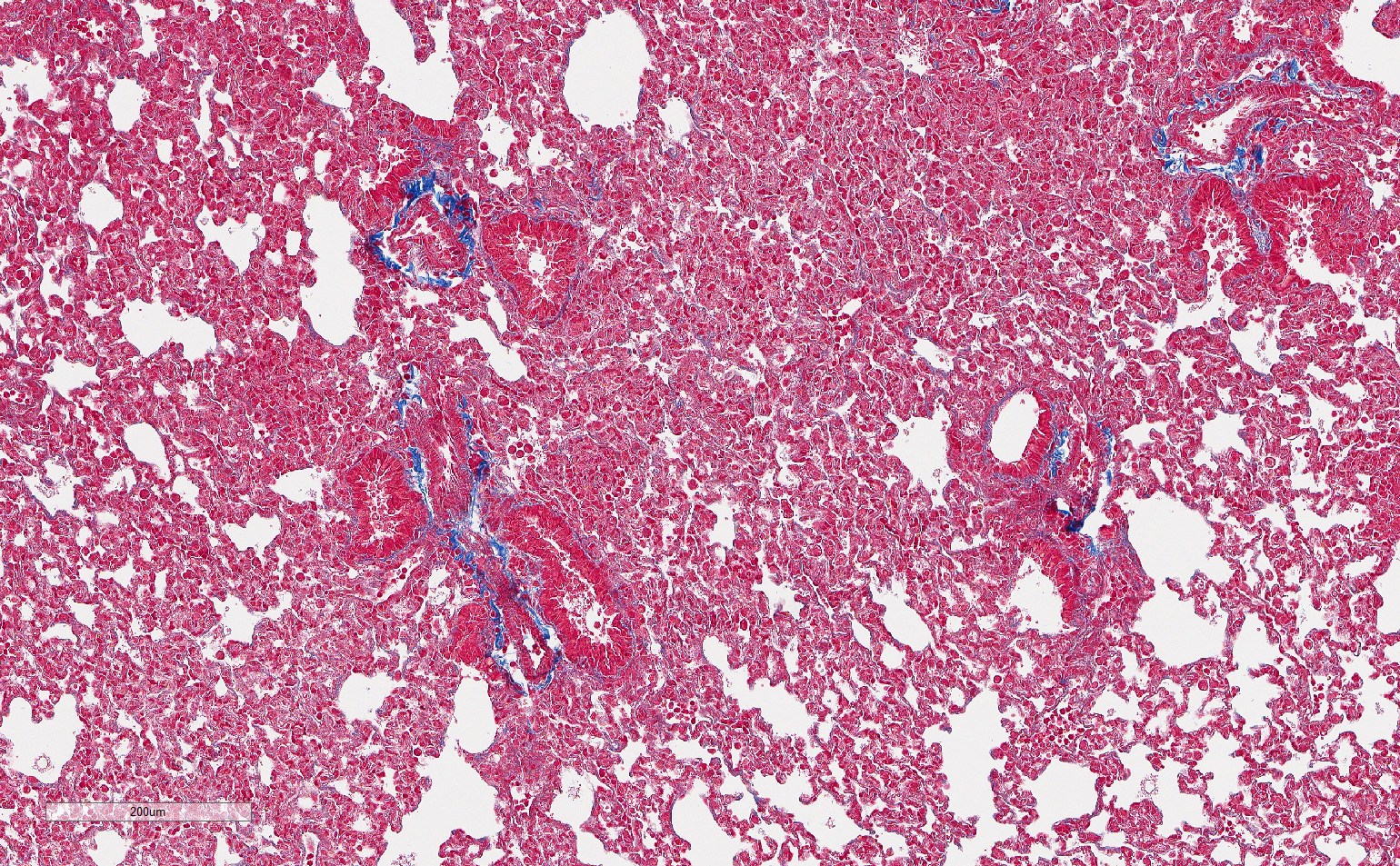

Supplement: Supplementary file 3 — Uncropped images and numerical data for Supplementary Fig. 1. [file 41589_2021_965_MOESM3_ESM.zip › Source_data_for_Supplementary_Figure_1/Uncropped images for Supplemental Figure 1D/Supplementary Figure 1D -r2c3.tif]

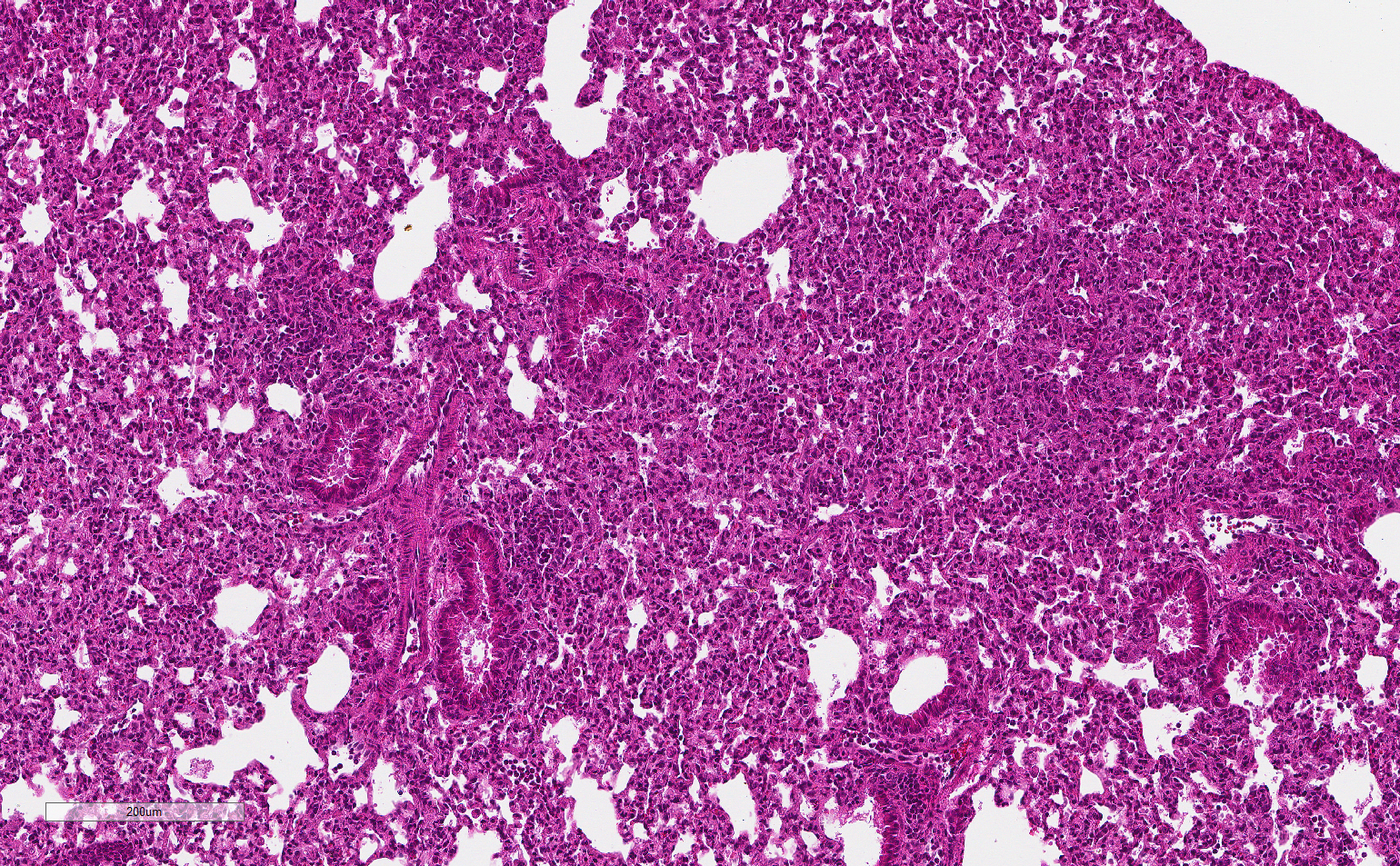

Supplement: Supplementary file 3 — Uncropped images and numerical data for Supplementary Fig. 1. [file 41589_2021_965_MOESM3_ESM.zip › Source_data_for_Supplementary_Figure_1/Uncropped images for Supplemental Figure 1D/Supplementary Figure 1D -r2c2.tif]

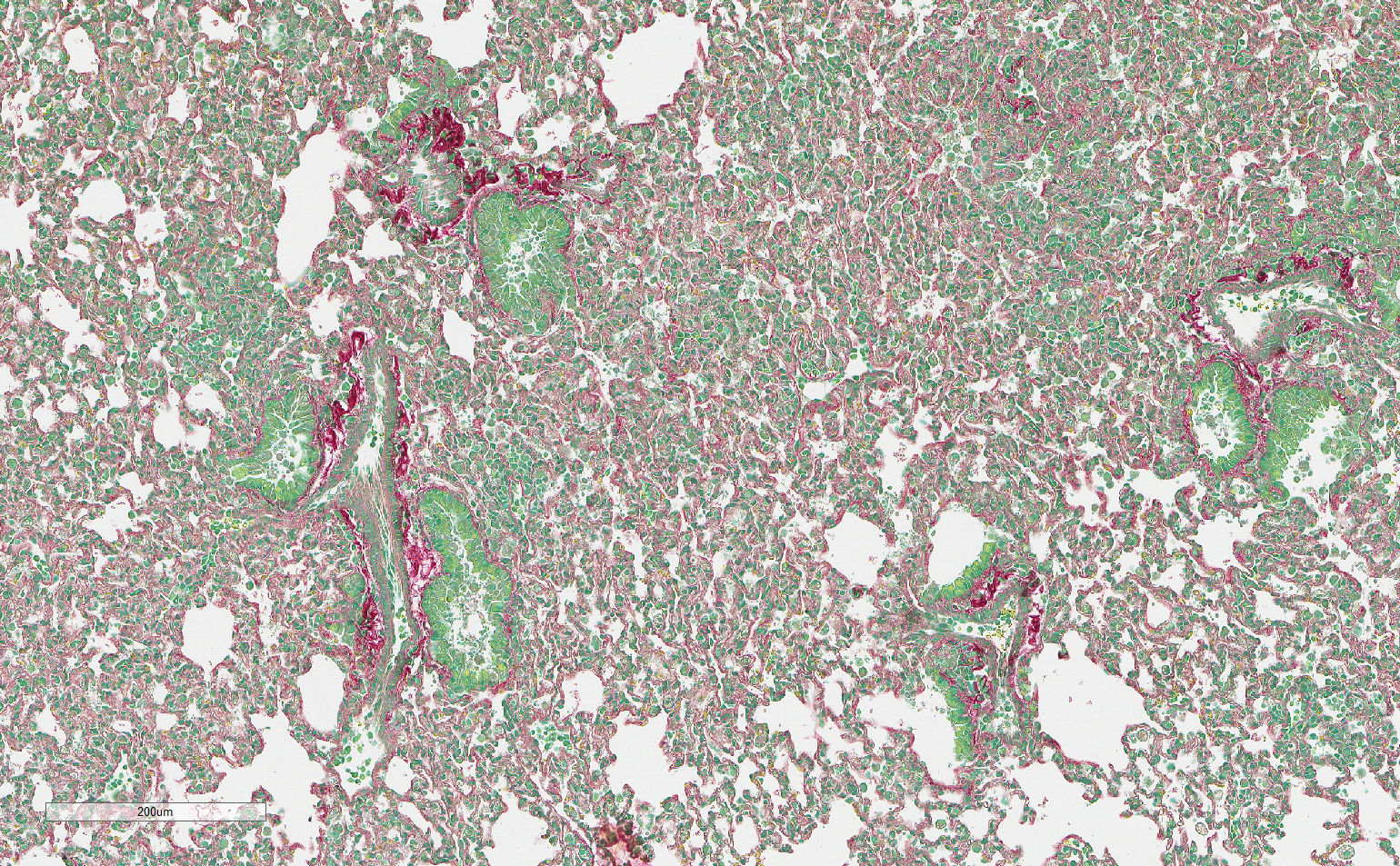

Supplement: Supplementary file 3 — Uncropped images and numerical data for Supplementary Fig. 1. [file 41589_2021_965_MOESM3_ESM.zip › Source_data_for_Supplementary_Figure_1/Uncropped images for Supplemental Figure 1D/Supplementary Figure 1D -r2c4.tif]

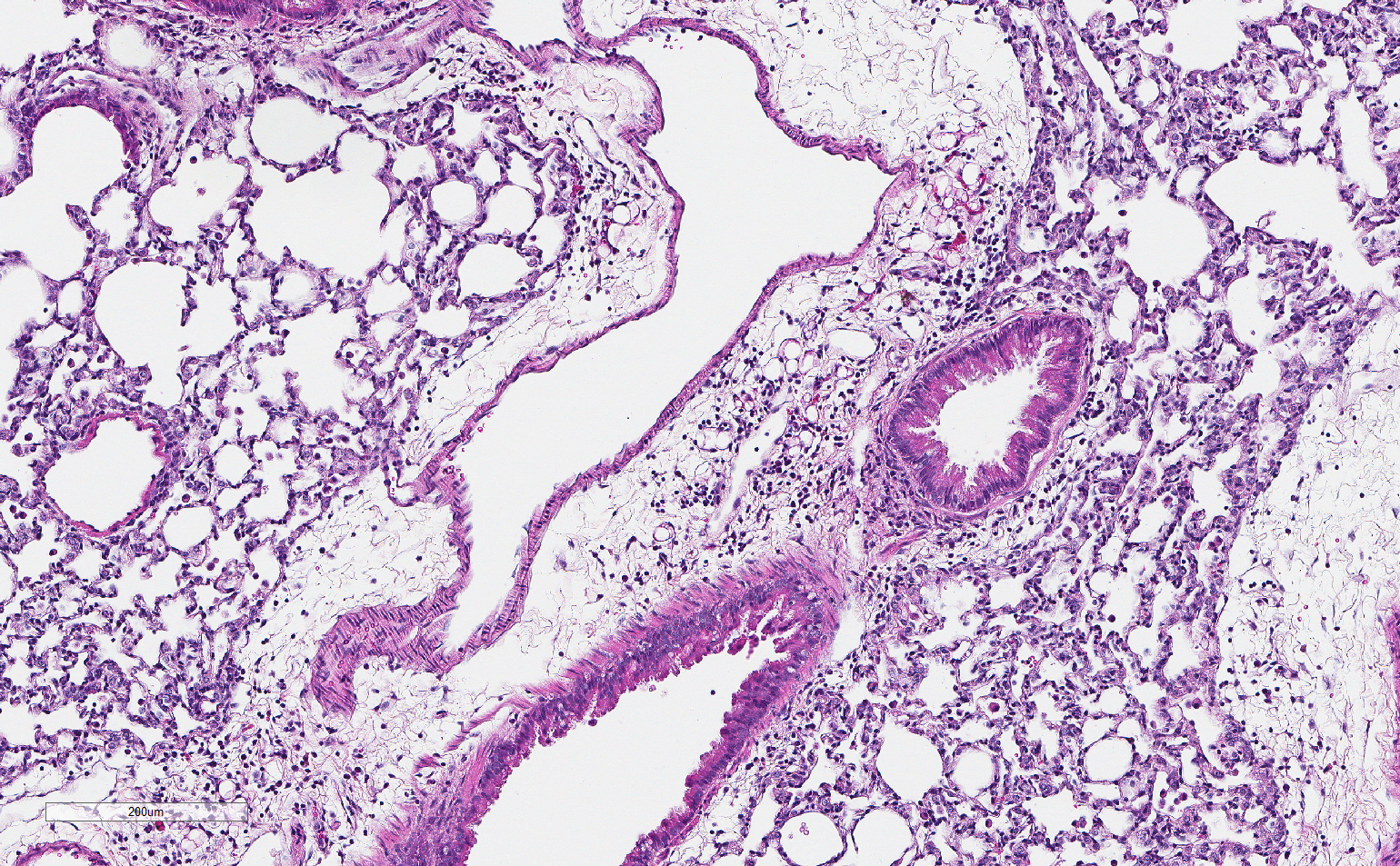

Supplement: Supplementary file 6 — Uncropped images and numerical data for Supplementary Fig. 6. [file 41589_2021_965_MOESM6_ESM.zip › Source_data_for_Supplementary_Figure_6/Uncropped images for Supplemental Figure 6D/Supplementary Figure 6D -r2c4.tif]

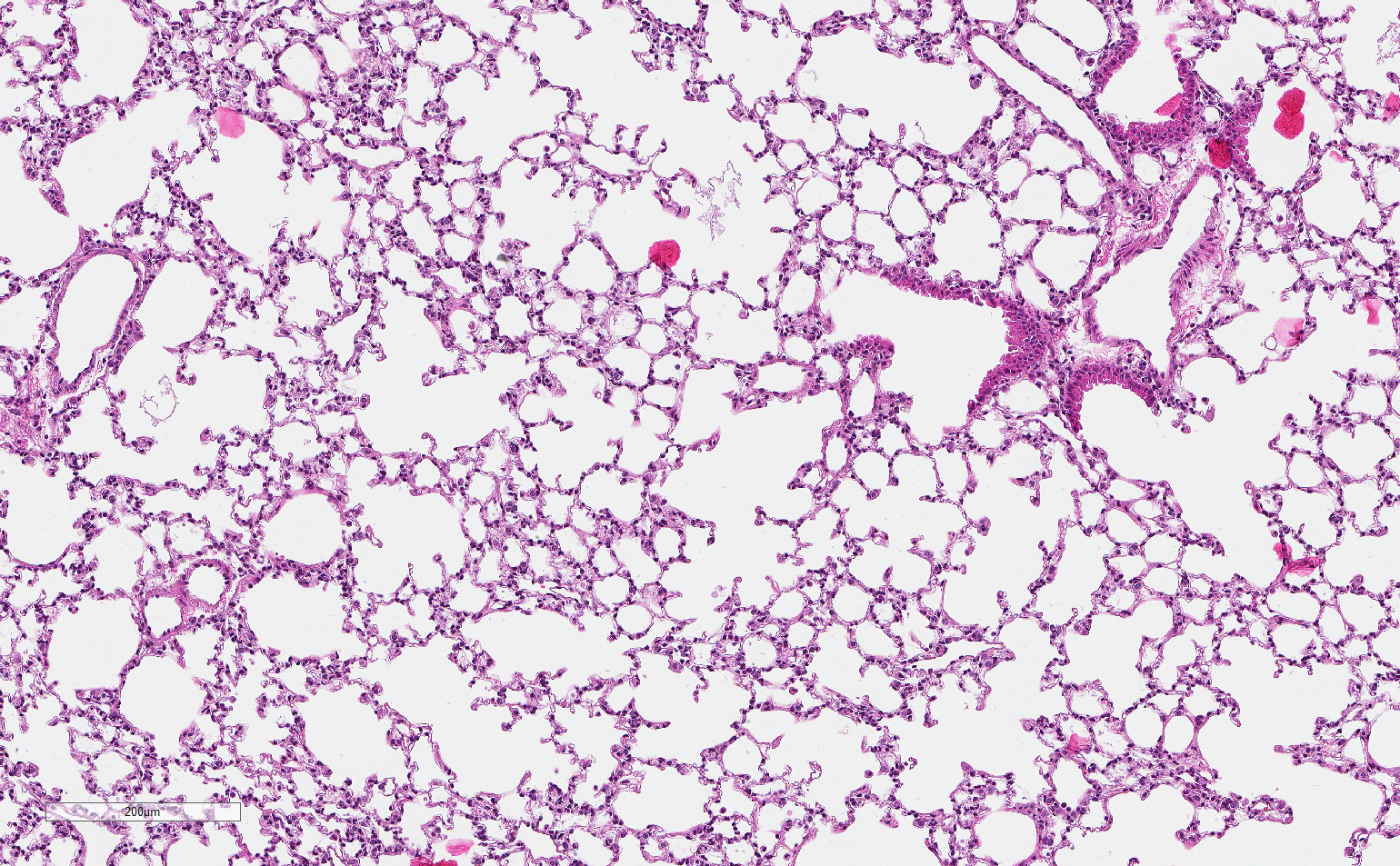

Supplement: Supplementary file 6 — Uncropped images and numerical data for Supplementary Fig. 6. [file 41589_2021_965_MOESM6_ESM.zip › Source_data_for_Supplementary_Figure_6/Uncropped images for Supplemental Figure 6D/Supplementary Figure 6D -r2c1.tif]

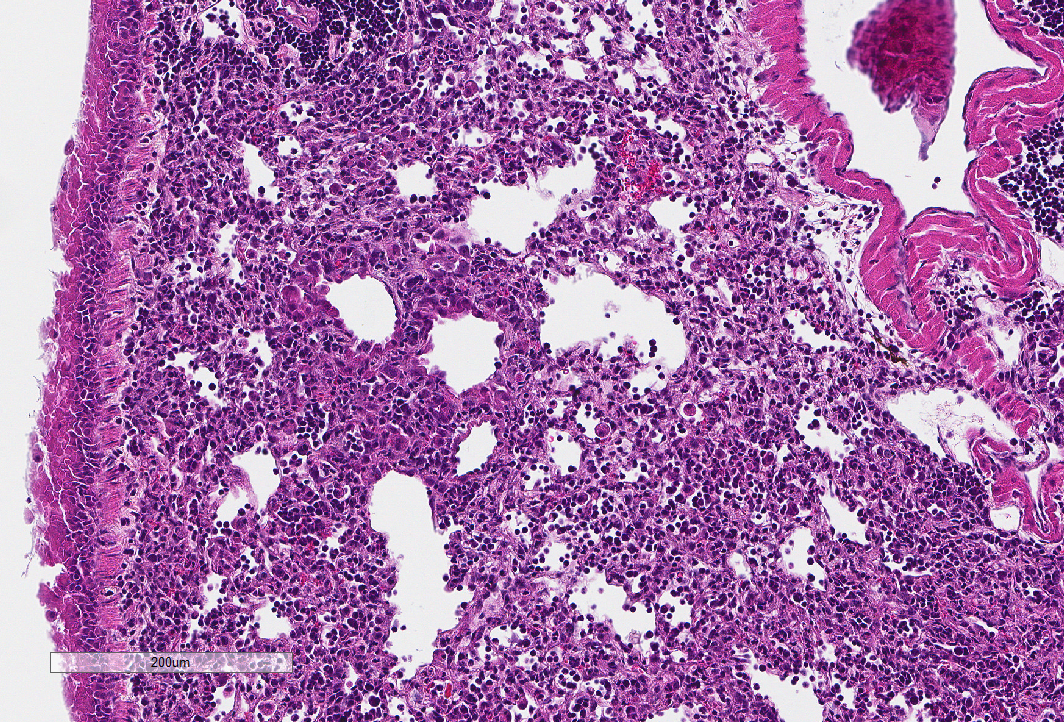

Supplement: Supplementary file 6 — Uncropped images and numerical data for Supplementary Fig. 6. [file 41589_2021_965_MOESM6_ESM.zip › Source_data_for_Supplementary_Figure_6/Uncropped images for Supplemental Figure 6D/Supplementary Figure 6D -r2c2.tif]

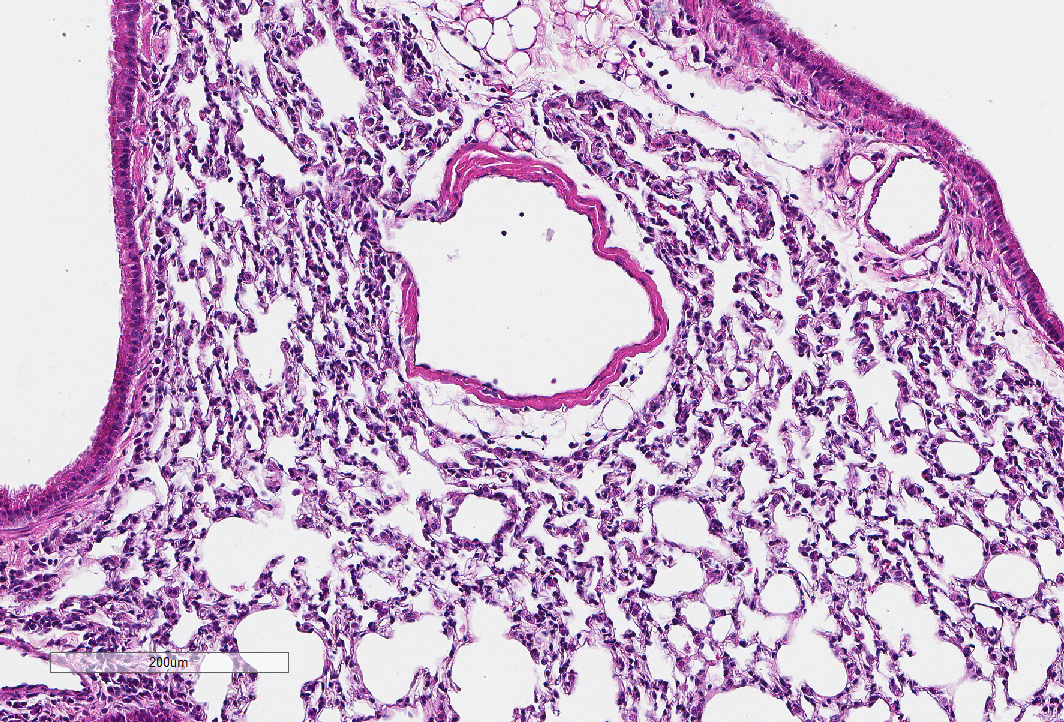

Supplement: Supplementary file 6 — Uncropped images and numerical data for Supplementary Fig. 6. [file 41589_2021_965_MOESM6_ESM.zip › Source_data_for_Supplementary_Figure_6/Uncropped images for Supplemental Figure 6D/Supplementary Figure 6D -r2c3.tif]

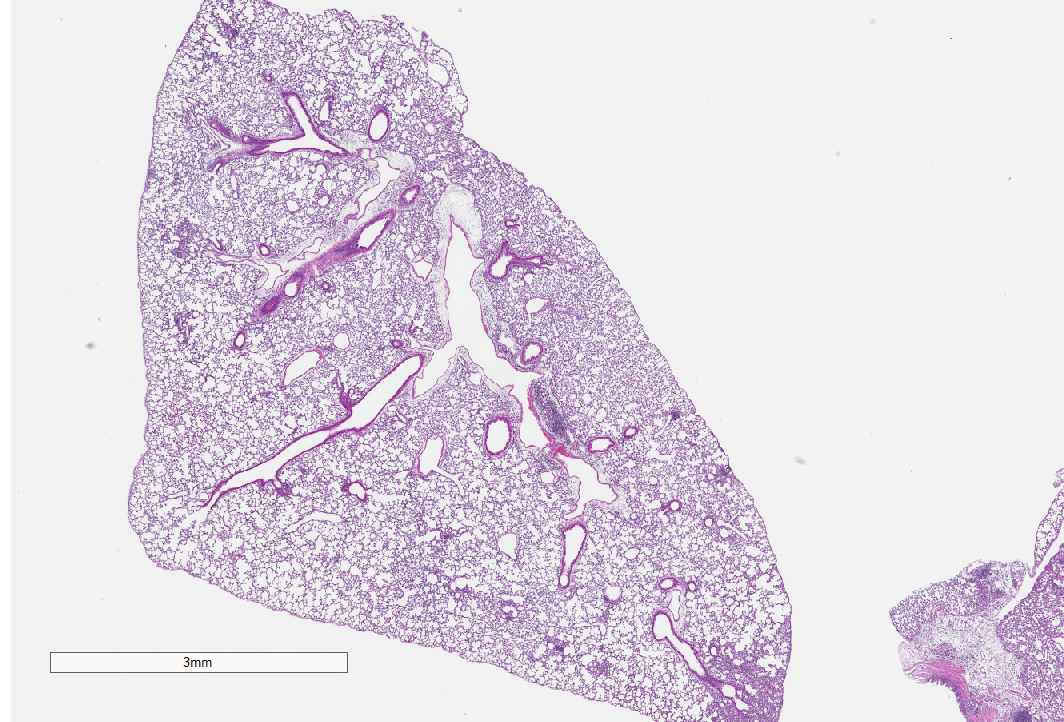

Supplement: Supplementary file 6 — Uncropped images and numerical data for Supplementary Fig. 6. [file 41589_2021_965_MOESM6_ESM.zip › Source_data_for_Supplementary_Figure_6/Uncropped images for Supplemental Figure 6D/Supplementary Figure 6D -r1c4.tif]

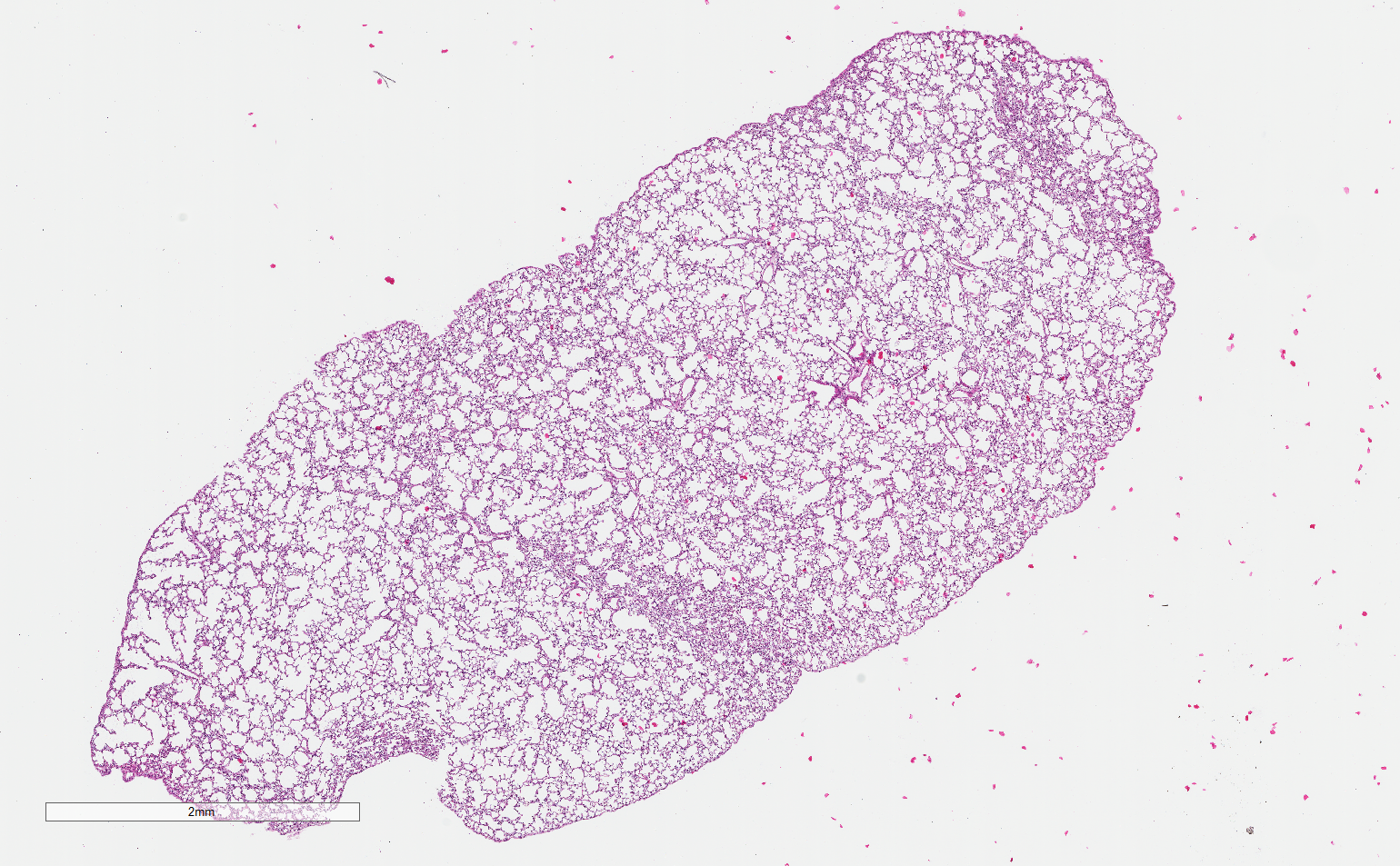

Supplement: Supplementary file 6 — Uncropped images and numerical data for Supplementary Fig. 6. [file 41589_2021_965_MOESM6_ESM.zip › Source_data_for_Supplementary_Figure_6/Uncropped images for Supplemental Figure 6D/Supplementary Figure 6D -r1c1.tif]

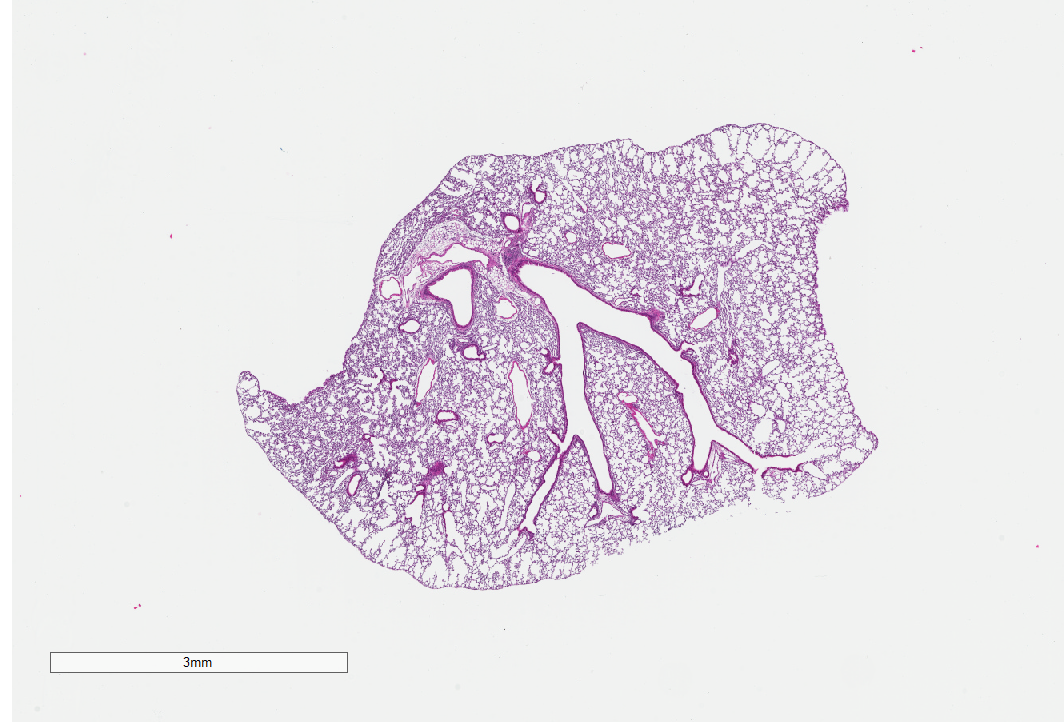

Supplement: Supplementary file 6 — Uncropped images and numerical data for Supplementary Fig. 6. [file 41589_2021_965_MOESM6_ESM.zip › Source_data_for_Supplementary_Figure_6/Uncropped images for Supplemental Figure 6D/Supplementary Figure 6D -r1c3.tif]

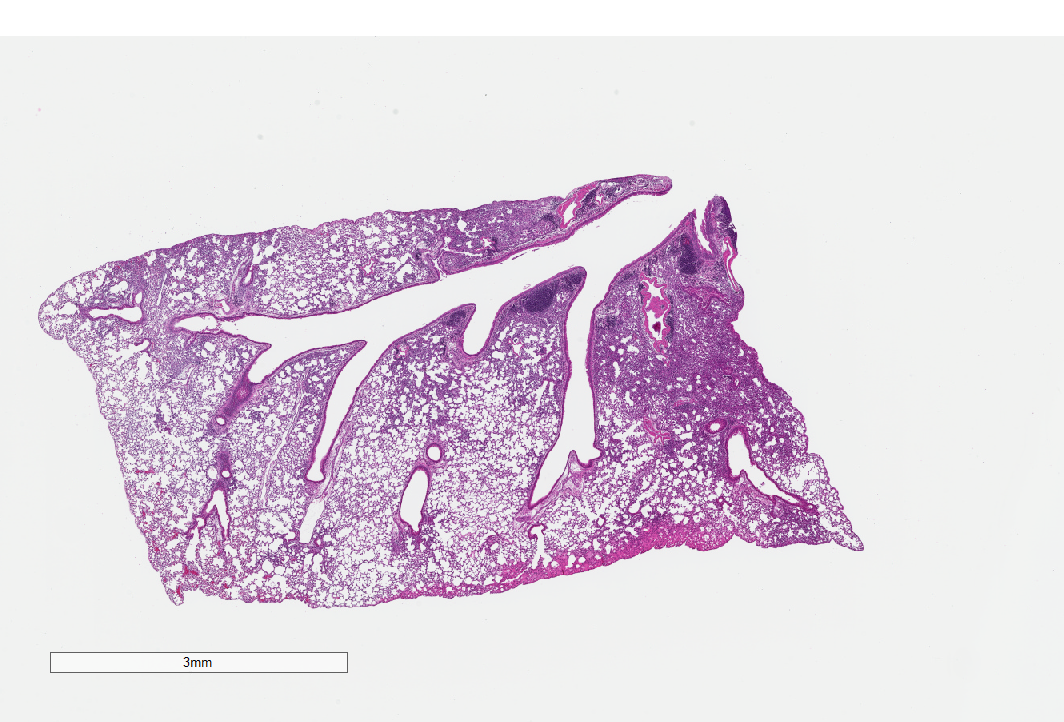

Supplement: Supplementary file 6 — Uncropped images and numerical data for Supplementary Fig. 6. [file 41589_2021_965_MOESM6_ESM.zip › Source_data_for_Supplementary_Figure_6/Uncropped images for Supplemental Figure 6D/Supplementary Figure 6D -r1c2.tif]

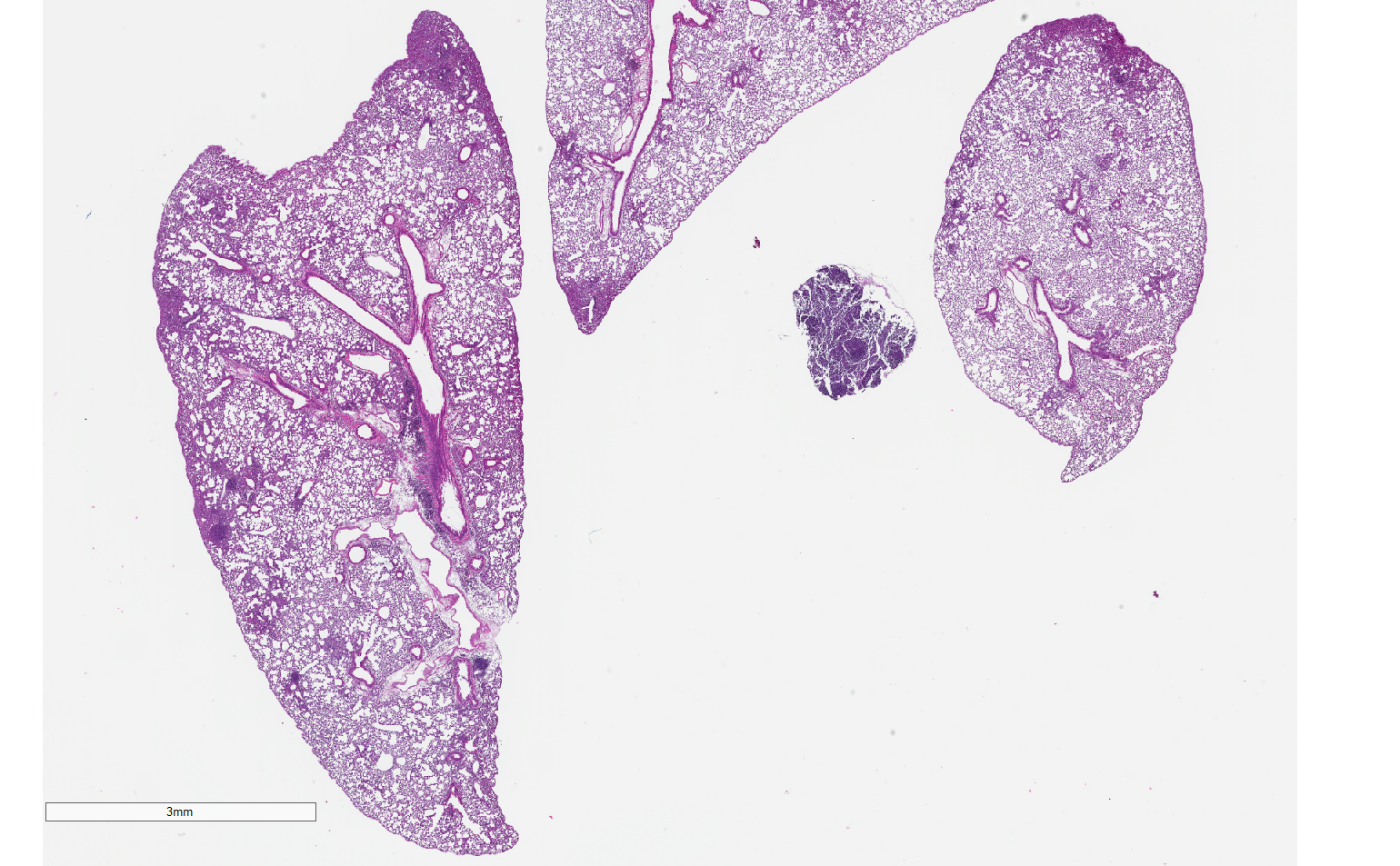

Supplement: Supplementary file 9 — Uncropped images and numerical data for Supplementary Fig. 10. [file 41589_2021_965_MOESM9_ESM.zip › Source_data_for_Supplementary_Figure_10/Uncropped images for Supplemental Figure 10C/Supplementary Figure 10C -r1c4.tif]

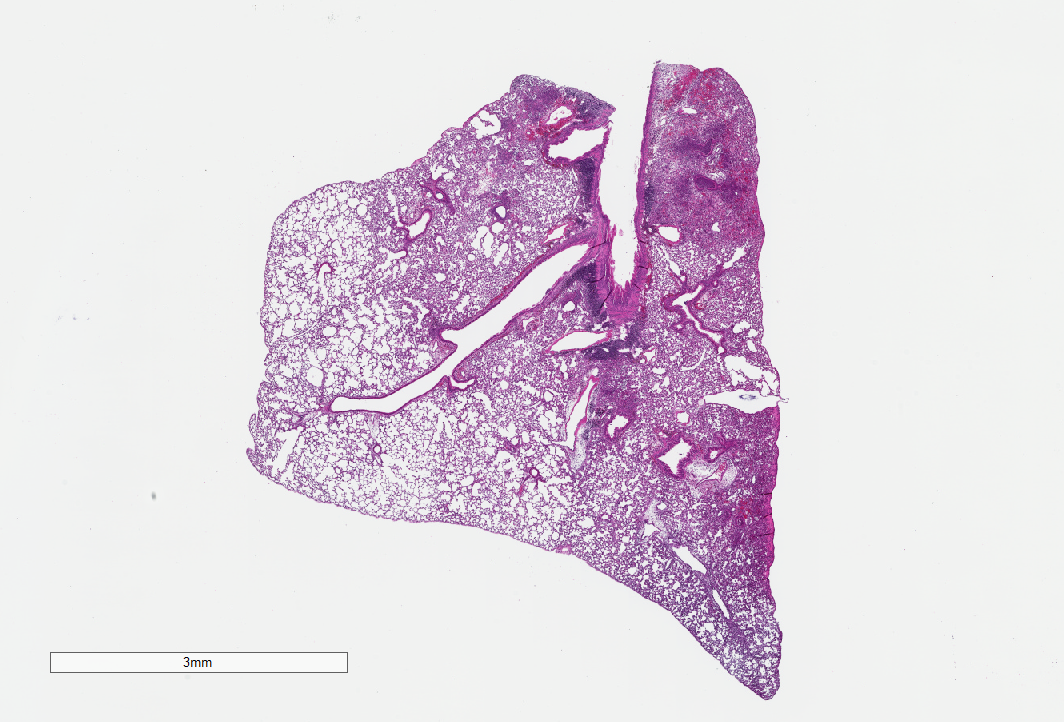

Supplement: Supplementary file 9 — Uncropped images and numerical data for Supplementary Fig. 10. [file 41589_2021_965_MOESM9_ESM.zip › Source_data_for_Supplementary_Figure_10/Uncropped images for Supplemental Figure 10C/Supplementary Figure 10C -r1c2.tif]

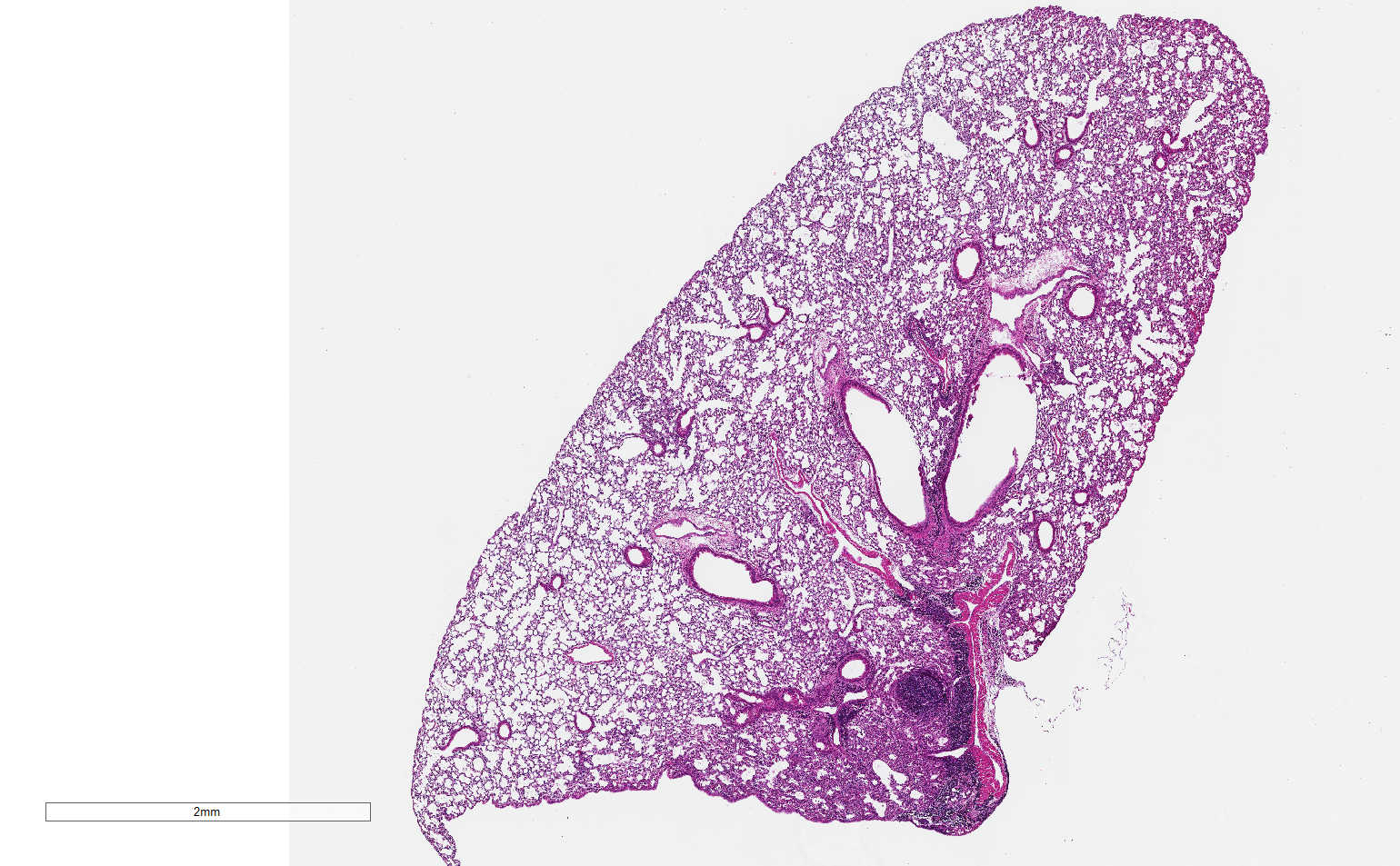

Supplement: Supplementary file 9 — Uncropped images and numerical data for Supplementary Fig. 10. [file 41589_2021_965_MOESM9_ESM.zip › Source_data_for_Supplementary_Figure_10/Uncropped images for Supplemental Figure 10C/Supplementary Figure 10C -r1c3.tif]

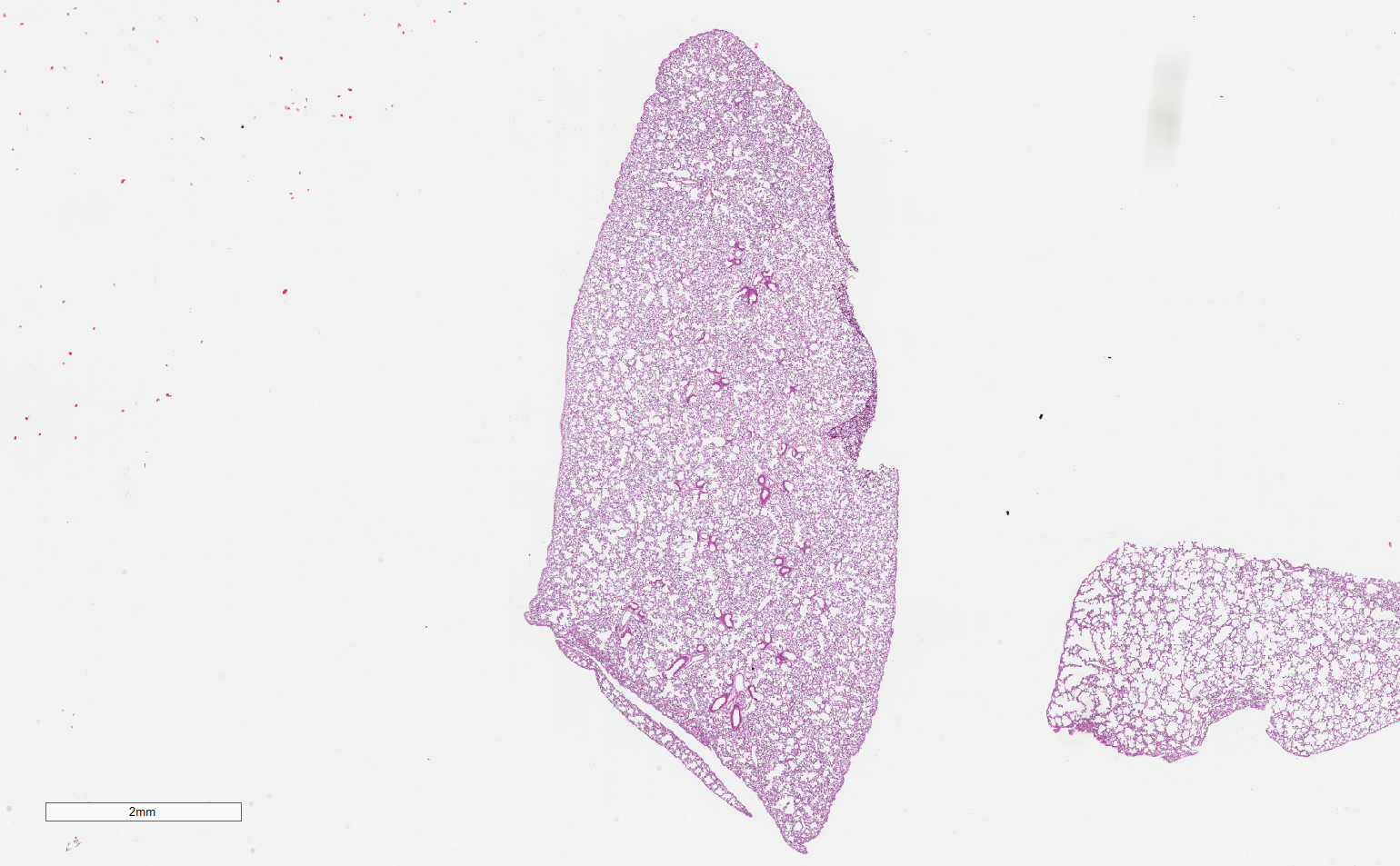

Supplement: Supplementary file 9 — Uncropped images and numerical data for Supplementary Fig. 10. [file 41589_2021_965_MOESM9_ESM.zip › Source_data_for_Supplementary_Figure_10/Uncropped images for Supplemental Figure 10C/Supplementary Figure 10C -r1c1.tif]

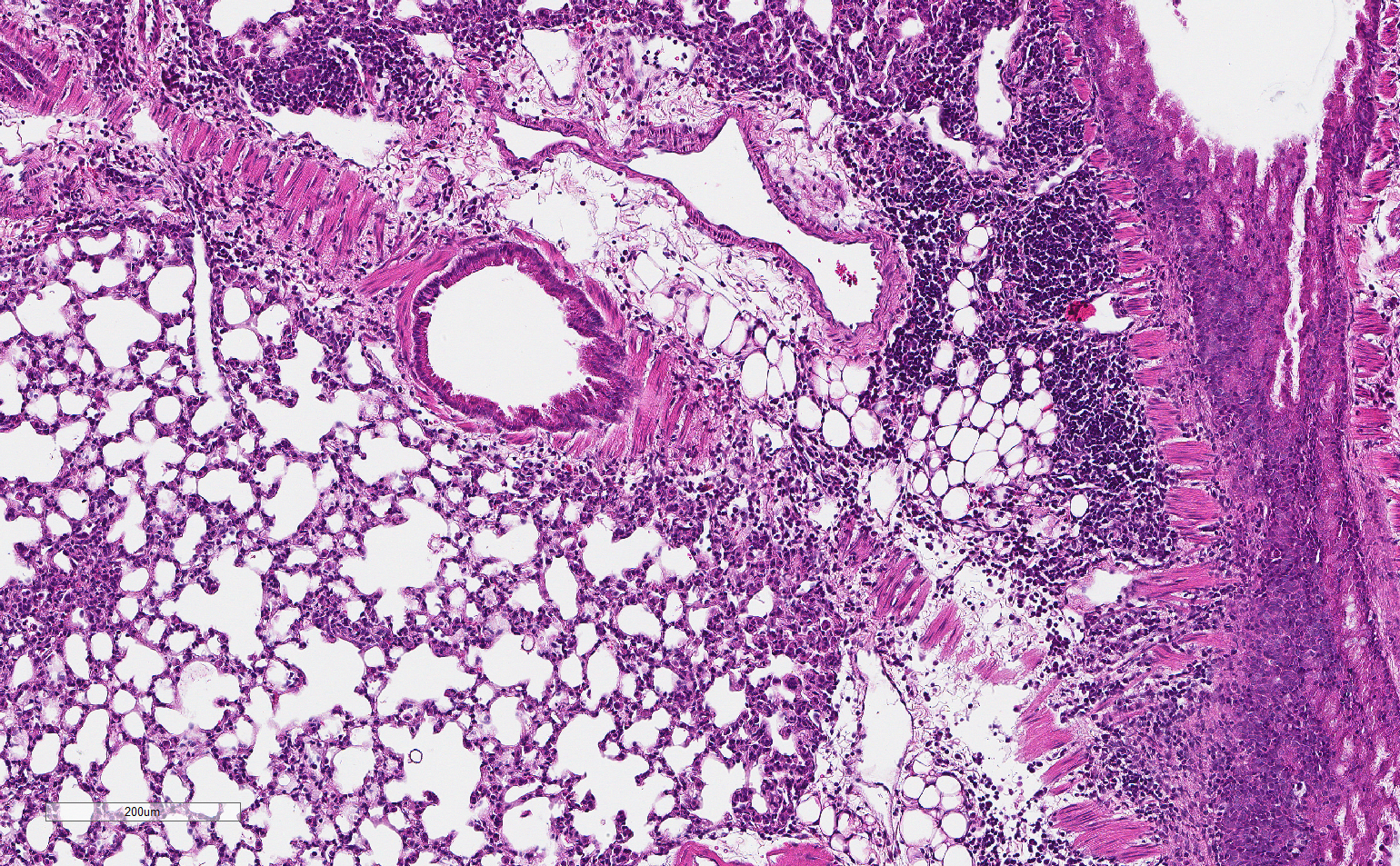

Supplement: Supplementary file 9 — Uncropped images and numerical data for Supplementary Fig. 10. [file 41589_2021_965_MOESM9_ESM.zip › Source_data_for_Supplementary_Figure_10/Uncropped images for Supplemental Figure 10C/Supplementary Figure 10C -r2c4.tif]

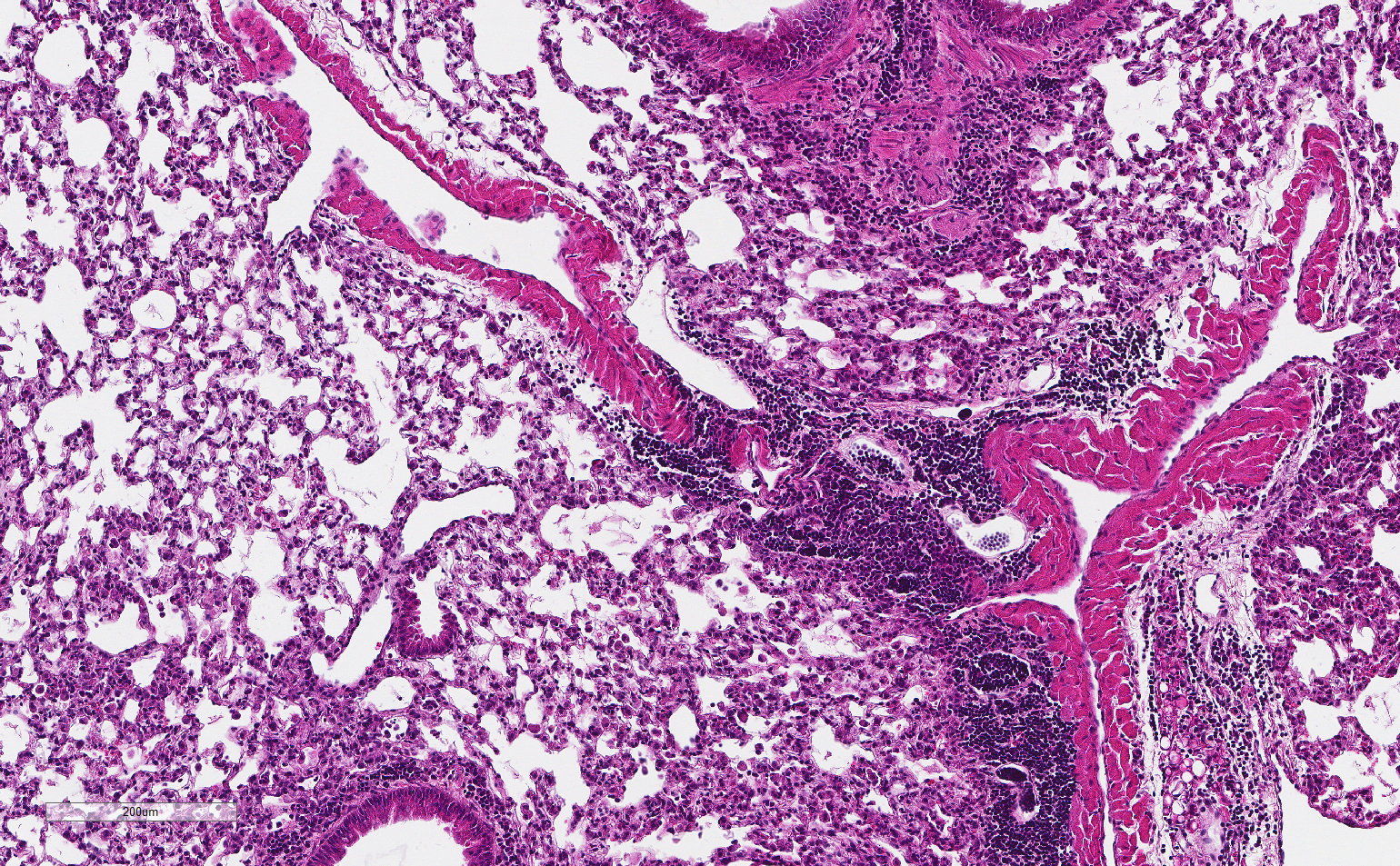

Supplement: Supplementary file 9 — Uncropped images and numerical data for Supplementary Fig. 10. [file 41589_2021_965_MOESM9_ESM.zip › Source_data_for_Supplementary_Figure_10/Uncropped images for Supplemental Figure 10C/Supplementary Figure 10C -r2c3.tif]

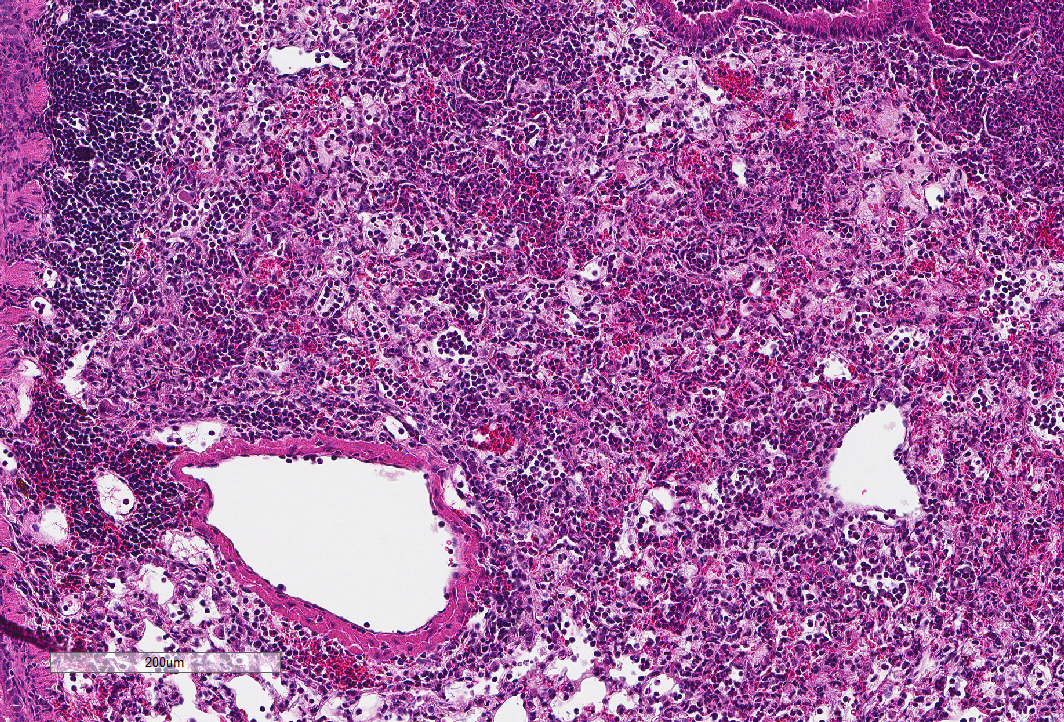

Supplement: Supplementary file 9 — Uncropped images and numerical data for Supplementary Fig. 10. [file 41589_2021_965_MOESM9_ESM.zip › Source_data_for_Supplementary_Figure_10/Uncropped images for Supplemental Figure 10C/Supplementary Figure 10C -r2c2.tif]

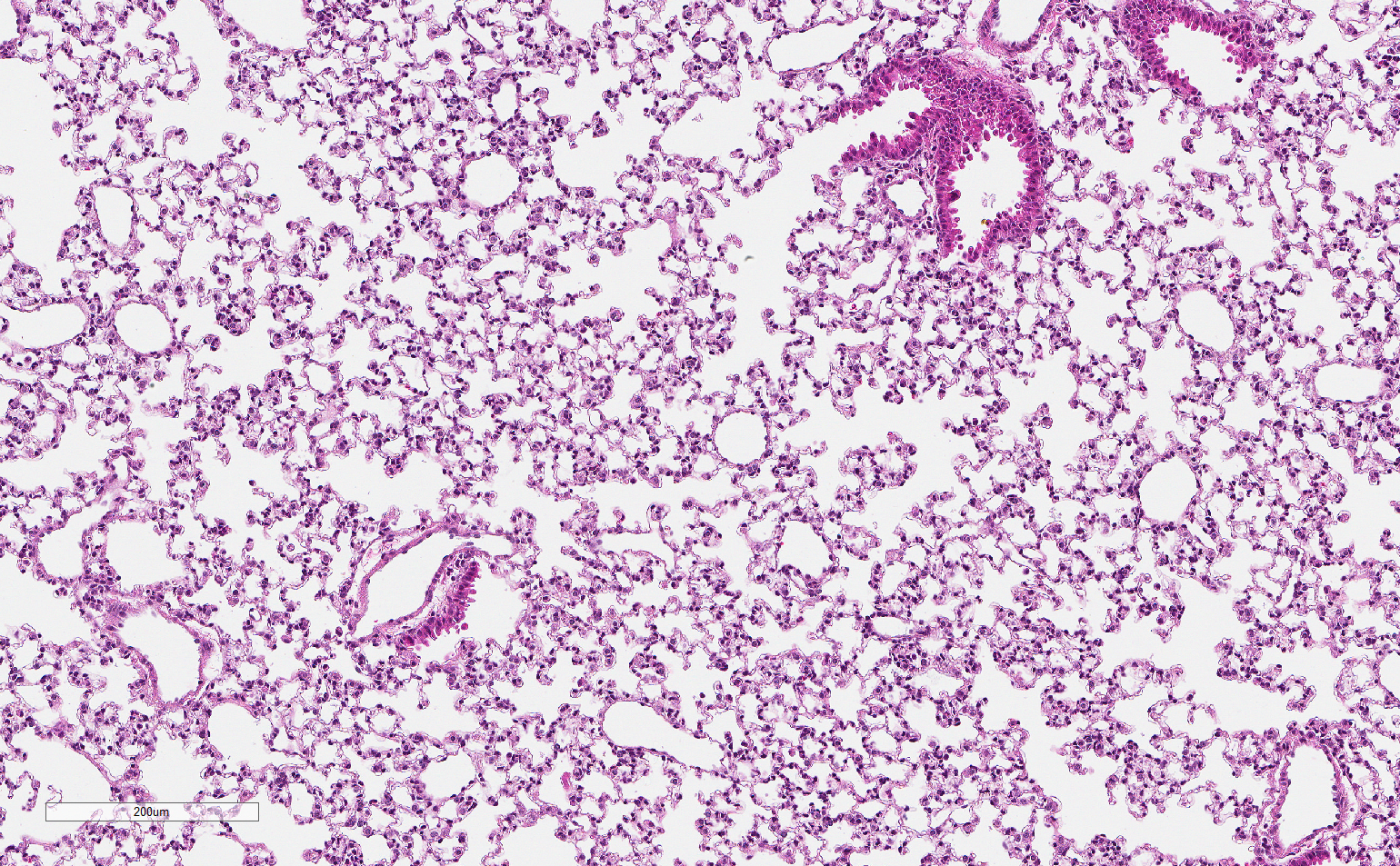

Supplement: Supplementary file 9 — Uncropped images and numerical data for Supplementary Fig. 10. [file 41589_2021_965_MOESM9_ESM.zip › Source_data_for_Supplementary_Figure_10/Uncropped images for Supplemental Figure 10C/Supplementary Figure 10C -r2c1.tif]

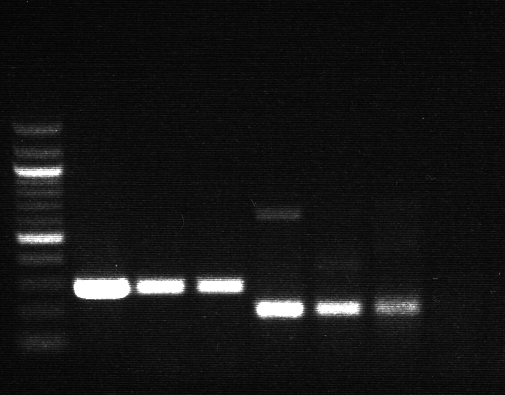

Supplement: Source Data Fig. 3 — Uncropped images and numerical data for Fig. 3. [file 41589_2021_965_MOESM11_ESM.zip › Source_data _for_Figure_3/Uncropped gel for Figure 3A/Figure 3A.tif]
